# Tumor Treating Fields dually activate STING and AIM2 inflammasomes to induce adjuvant immunity in glioblastoma

**DOI:** 10.1172/JCI149258

**Published:** 2022-04-15

**Authors:** Dongjiang Chen, Son B. Le, Tarun E. Hutchinson, Anda-Alexandra Calinescu, Mathew Sebastian, Dan Jin, Tianyi Liu, Ashley Ghiaseddin, Maryam Rahman, David D. Tran

**Affiliations:** 1 Division of Neuro-Oncology and Preston A. Wells, Jr. Center for Brain Tumor Therapy, Lillian S. Wells Department of Neurosurgery and; 2 Medical Scientist Training Program, University of Florida College of Medicine, Gainesville, Florida, USA.

**Keywords:** Oncology, Brain cancer, Cancer immunotherapy, Innate immunity

## Abstract

Tumor Treating Fields (TTFields), an approved therapy for glioblastoma (GBM) and malignant mesothelioma, employ noninvasive application of low-intensity, intermediate-frequency, alternating electric fields to disrupt the mitotic spindle, leading to chromosome missegregation and apoptosis. Emerging evidence suggests that TTFields may also induce inflammation. However, the mechanism underlying this property and whether it can be harnessed therapeutically are unclear. Here, we report that TTFields induced focal disruption of the nuclear envelope, leading to cytosolic release of large micronuclei clusters that intensely recruited and activated 2 major DNA sensors — cyclic GMP-AMP synthase (cGAS) and absent in melanoma 2 (AIM2) — and their cognate cGAS/stimulator of interferon genes (STING) and AIM2/caspase 1 inflammasomes to produce proinflammatory cytokines, type 1 interferons (T1IFNs), and T1IFN-responsive genes. In syngeneic murine GBM models, TTFields-treated GBM cells induced antitumor memory immunity and a cure rate of 42% to 66% in a *STING*- and *AIM2*-dependent manner. Using single-cell and bulk RNA sequencing of peripheral blood mononuclear cells, we detected robust post-TTFields activation of adaptive immunity in patients with GBM via a T1IFN-based trajectory and identified a gene panel signature of TTFields effects on T cell activation and clonal expansion. Collectively, these studies defined a therapeutic strategy using TTFields as cancer immunotherapy in GBM and potentially other solid tumors.

## Graphical abstract

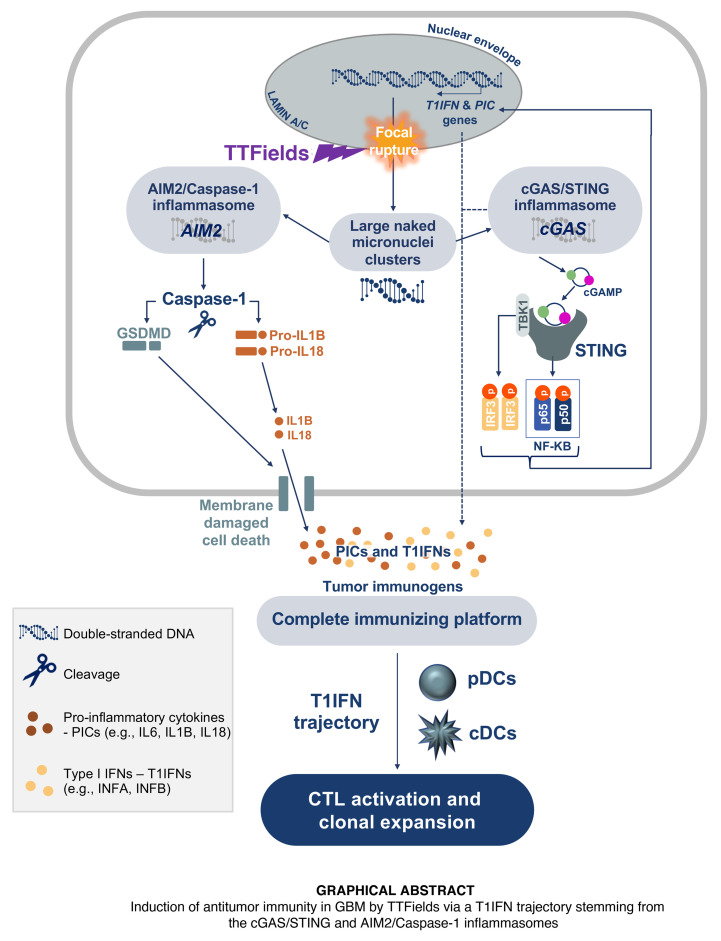

## Introduction

Glioblastoma (GBM) is the most common and lethal brain cancer in adults and one of the least immunogenic tumors (1). Recent work has revealed striking immune dysregulation and functional impairment in patients with GBM. Besides systemic T lymphopenia and anergy and dysfunctional cytokine profiles among others, GBM tumors also possess a profoundly immunosuppressed or cold tumor microenvironment (TME), characterized by scant tumor-infiltrating lymphocytes (TILs) and an abundance of inhibitory cells, including myeloid-derived suppressor cells (MDSCs) and regulatory T cells (Tregs). The cold GBM TME expresses high levels of immune checkpoint proteins (2), and is further complicated by tumor cells’ profound genetic heterogeneity (3). In addition, the blood brain barrier (BBB) prevents exposure of tumor-associated neoantigens to immune cells and vice versa, severely hindering immunotherapeutic efforts (2). Overcoming these hurdles promises a long-lasting, multilayered, immune-mediated tumor control. To “heat up” the cold GBM TME, recent efforts have focused on tumor cell–extrinsic pathways with mixed results, such as dendritic cell–based (DC-based) vaccination, immune checkpoint blockade, rewiring the cytokine milieu, or disrupting BBB integrity to recruit tumor-specific cytotoxic T lymphocytes (CTLs) (4). However, it remains a challenge to leverage a direct, active role of tumor cells in reversing the immunosuppressive state of the GBM TME.

By targeting the motility, alignment, and assembly of macromolecules required for the mitotic spindle structure during metaphase and the contractile ring during anaphase, telophase, and cytokinesis of the cell cycle, Tumor Treating Fields (TTFields) cause chromosome missegregation and breakage and incomplete cytoplasmic separation, respectively, leading to mitotic catastrophe and p53-dependent and -independent apoptosis (5–7). TTFields have also been demonstrated to target the DNA damage repair and breast cancer 1–mediated (BRCA1-mediated) homologous recombination pathways by interfering with DNA fork replication (8–10) and induce endoplasmic reticulum stress during mitosis to trigger adenosine monophosphate–activated protein kinase–dependent autophagosome formation, through increased lipidation of protein light chain 3 α/β-I (LC3A/B-I) to form LC3A/B-II (11). Recent reports also revealed TTFields’ ability to electroporate the plasma membrane of GBM cells, allowing particles up to 20 kDa to pass through (12), and to disrupt tight junction proteins (e.g., claudin 5 and ZO-1) of the BBB in small rodents, leading to increased BBB permeability (13). Whether TTFields affect the integrity of other cell membranes and the role this plays in antitumor activity are undefined. In clinical usage, some TTFields responders, especially with GBM, exhibited transient increased tumor-associated contrast enhancement and edema shortly after treatment initiation, often followed by a delayed, durable, objective radiographic response (14–19), suggesting the possibility of an inflammatory reaction promoted by TTFields in addition to or independent of its antimitotic activity. In murine models of solid tumors, TTFields were shown to stimulate immunogenic cell death (20) and promote immune cell recruitment (21), raising hope that TTFields may provide the needed stimuli to reverse local and systemic immunosuppression in patients with GBM. However, the molecular mechanism is unclear and clinical evidence is lacking.

## Results

### TTFields induce formation of cytosolic micronuclei clusters that recruit cGAS and AIM2.

**Figure 1:**
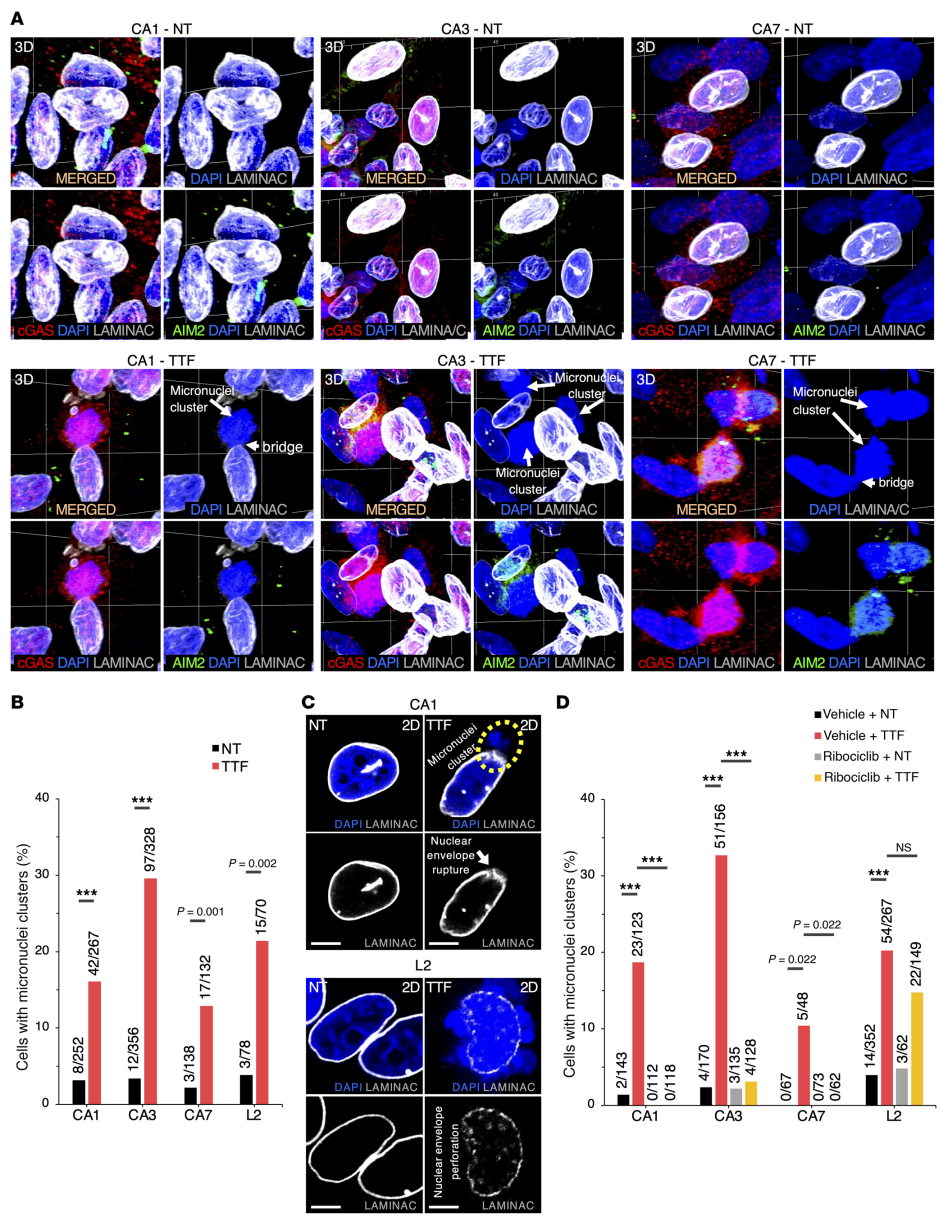
TTFields-induced cytosolic micronuclei clusters recruit cGAS and AIM2 in patient-derived GSCs. (See Supplemental Figures 1–7). (**A**) 3D confocal images showing immunofluorescence staining (IF) for cGAS and AIM2 and counterstained with DAPI for DNA in CA1, CA3, and CA7 GSCs either nontreated (NT) (top) or treated with TTFields at 200 kHz (TTF) (bottom) for 24 hours. Large micronuclei clusters extend directly from the true nuclei through a narrow bridge. Each square is 30 μm^2^; *z* height is 15 μm. (**B**) A bar plot showing percentages of GSCs with cGAS and AIM2-recruited cytosolic large micronuclei clusters and nuclear protrusions over the total cells counted in the experiments in **A**. Fisher’s exact test was used to compare 2 groups within each cell line. ***P < 0.001. (**C**) Representative confocal images showing IF of LAMINAC and DAPI counterstain in CA1 and L2 GSCs either NT or TTF for 24 hours, showing a focal rupture (CA1) and scattered perforations (L2) of the nuclear envelope leading to a large micronuclei cluster (broken yellow oval) and several nuclear protrusions, respectively. Scale bars: 10 μm. (**D**) A bar plot showing percentages of GSCs with cGAS and AIM2-recruited cytosolic large micronuclei clusters over the total cells counted, following pretreatment with either the vehicle or ribociclib (4.5 μM) to induce G_1_ arrest, followed by TTFields treatment for 24 hours, demonstrating that S-phase entry is required for TTFields-induced cytosolic micronuclei clusters. L2 cells are relatively resistant to ribociclib. Fisher’s exact test with adjustments for multiple comparisons was used. ***P < 0.001. NS, not significant. All data are representative of at least 3 independent experiments.

A potential link between TTFields and immune activation is cytosolic micronuclei created by TTFields-induced mitotic disruption (22). We detected isolated small cytosolic micronuclei by DAPI counterstaining, which were independent of TTFields treatment. More importantly, however, in 4 patient-derived GBM cancer stem–like cell (GSC) lines (CA1, CA3, CA7, and L2) and 3 human GBM cell lines (U87MG, LN428, and LN827), we found large clusters of cytosolic micronuclei projecting directly from the true nuclei through focal, narrow bridges at 5- to greater than 50-fold higher frequency consistently across all lines treated for 24 hours with TTFields (200 kHz unless otherwise noted) as compared with nontreated cells (Figure 1, A and B, Supplemental Figure 1, A and B, and Supplemental Figure 2B; supplemental material available online with this article; https://doi.org/10.1172/JCI149258DS1). In solid tumors, cytosolic naked DNA signifies aberrant host DNA metabolism and is recognized by DNA sensors, including cyclic GMP-AMP synthase (cGAS) (23) and absent in melanoma 2 (AIM2) (24, 25), thereby activating their cognate cGAS/stimulator of interferon genes (STING) and AIM2/caspase 1 inflammasomes to trigger danger signals (26). Either or both DNA sensors were recruited to and densely concentrated in all observable TTFields-induced large cytosolic micronuclei clusters in all 7 lines (Figure 1, A and B, Supplemental Figure 1A, Supplemental Figure 2B, and Supplemental Figure 3), indicating that these clusters were unshielded by the nuclear envelope. We also observed a redistribution of cGAS and AIM2 from a scattered pattern to the perinuclear region in some GBM cells, even in those without micronuclei clusters (Supplemental Figure 1C). Notably, large cGAS- and AIM2-recruited cytosolic micronuclei clusters were also observed in the human lung and pancreatic adenocarcinoma cell lines A549 and PANC-1, respectively, after a 24-hour exposure to TTFields at 150 kHz (Supplemental Figure 4, A and C), suggesting that this phenomenon is common in tumor cells and may manifest TTFields’ general effects on the nuclear envelope.

To assess the integrity of the nuclear envelope under TTFields, we determined the distribution of lamin A and C (LAMINAC), 2 major structural proteins lining the nuclear envelope’s interior (27), in the 7 GBM cell lines before and after TTFields. LAMINAC disorganization was observed specifically at sites of cytosolic micronuclei cluster protrusions in TTFields-treated cells, leading to focal rupture and perforations of the nuclear envelope (Figure 1C and Supplemental Figure 5), and thus arguing against these clusters representing chromosome condensation during prometaphase, when nuclear envelope dissolution is uniform and complete rather than focal (28). Moreover, most of the affected cells were not in metaphase, prompting the question of whether cell cycle entry is required for TTFields’ effects on the nuclear envelope and independent of its antimitotic effects through spindle disruption during metaphase (22). To address this question, we pretreated cells for 24 hours prior to and during the 24-hour exposure to TTFields with ribociclib (4.5 μM), a potent inhibitor of cyclin-dependent kinases 4 and 6, to induce G_1_/S arrest (ref. 29, Supplemental Figure 2C, and Supplemental Figure 6). In all GBM lines except for L2, the rates of formation of micronuclei clusters that recruit cGAS and AIM2 consistently decreased by 5- to greater than 50-fold after TTFields in ribociclib-arrested compared with cycling cells, while ribociclib alone did not increase micronuclei clusters (Figure 1D and Supplemental Figures 2 and 3). L2 cells were relatively resistant to ribociclib and, as a result, the frequency of TTFields-induced micronuclei clusters was minimally impacted. These results indicate that S-phase entry is necessary for TTFields-induced nuclear envelope disruption and cytosolic micronuclei cluster formation. In contrast, isolated small micronuclei and fragmented nuclei were independent of TTFields and cell cycle, shielded by a LAMINAC-based envelope, and did not recruit cGAS and AIM2 (Supplemental Figure 7).

Overall, TTFields generate large cytosolic naked micronuclei clusters in GBM and other cancer cell types through focal disruption of the nuclear envelope, thereby recruiting cGAS and AIM2 to create a ripe condition for activation of their cognate inflammasomes.

### TTFields activate the cGAS/STING and AIM2/caspase 1 inflammasomes.

**Figure 2:**
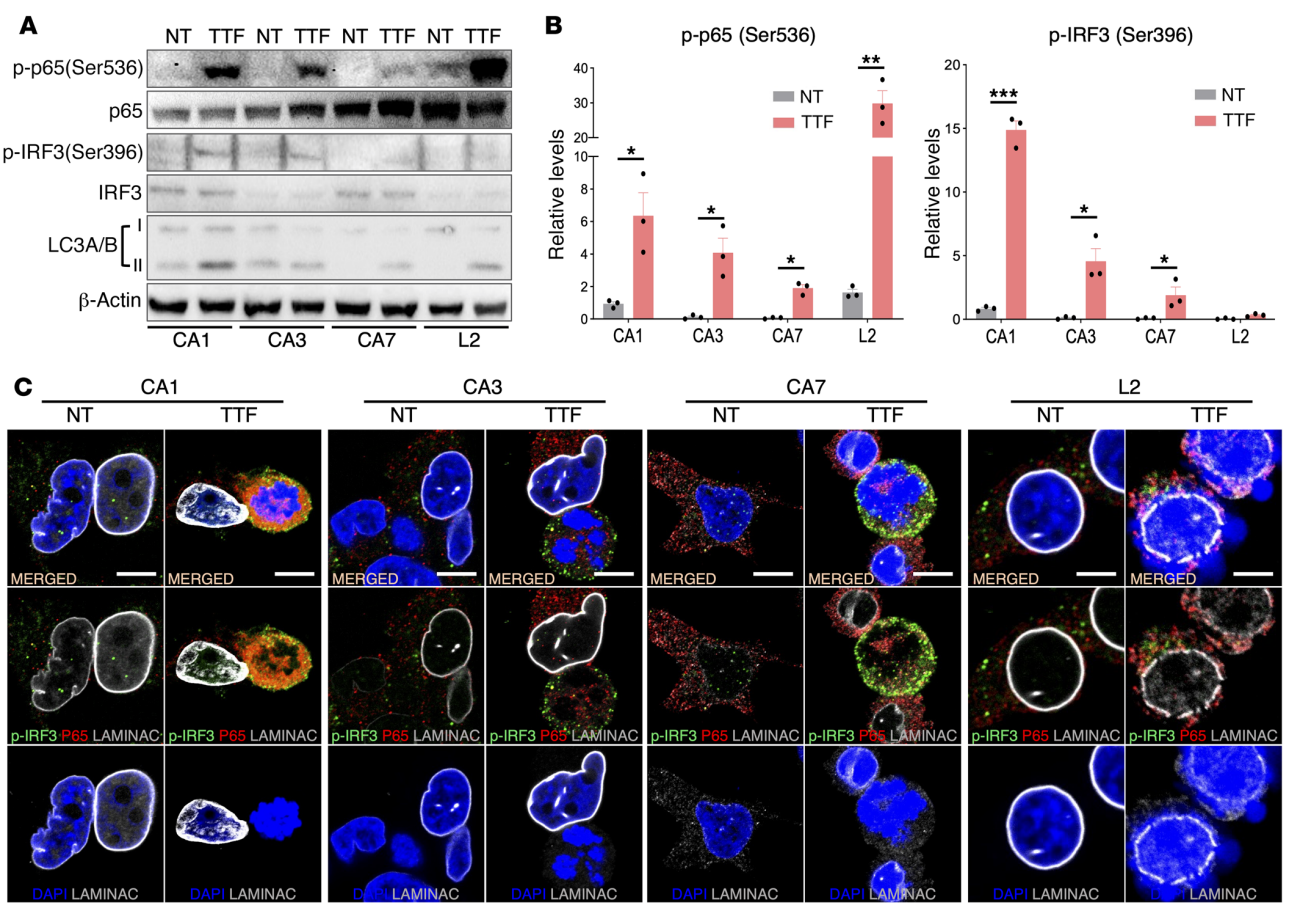
TTFields activate the cGAS/STING inflammasome in GSCs. (See Supplemental Figure 8). (**A** and **B**) The cGAS/STING inflammasome’s components IRF3 and p65 were activated following 24 hours of TTFields, as determined by immunoblotting for p-IRF3 and p-p65 in total lysate (**A**) and quantified by densitometry relative to total IRF3 and p65 levels and normalized to β-actin, with values for the nontreated set to 1 (**B**) in the 4 GSC lines. LC3A/B-I and -II were used to confirm the general TTFields effects. (**C**) Confocal images of IF demonstrating increased concentration and recruitment of p-IRF3 and p65 within cytosolic micronuclei clusters and protrusions after 24-hour treatment with TTFields with DAPI counterstain in the 4 GSC lines. Scale bars: 10 μm. All experiments used triplicate samples and were repeated at least 3 times. Data are represented as mean ± SEM. Analyses were performed using Student’s *t* test with a 2-tailed distribution. *P < 0.05, **P < 0.01, ***P < 0.001.

**Figure 3:**
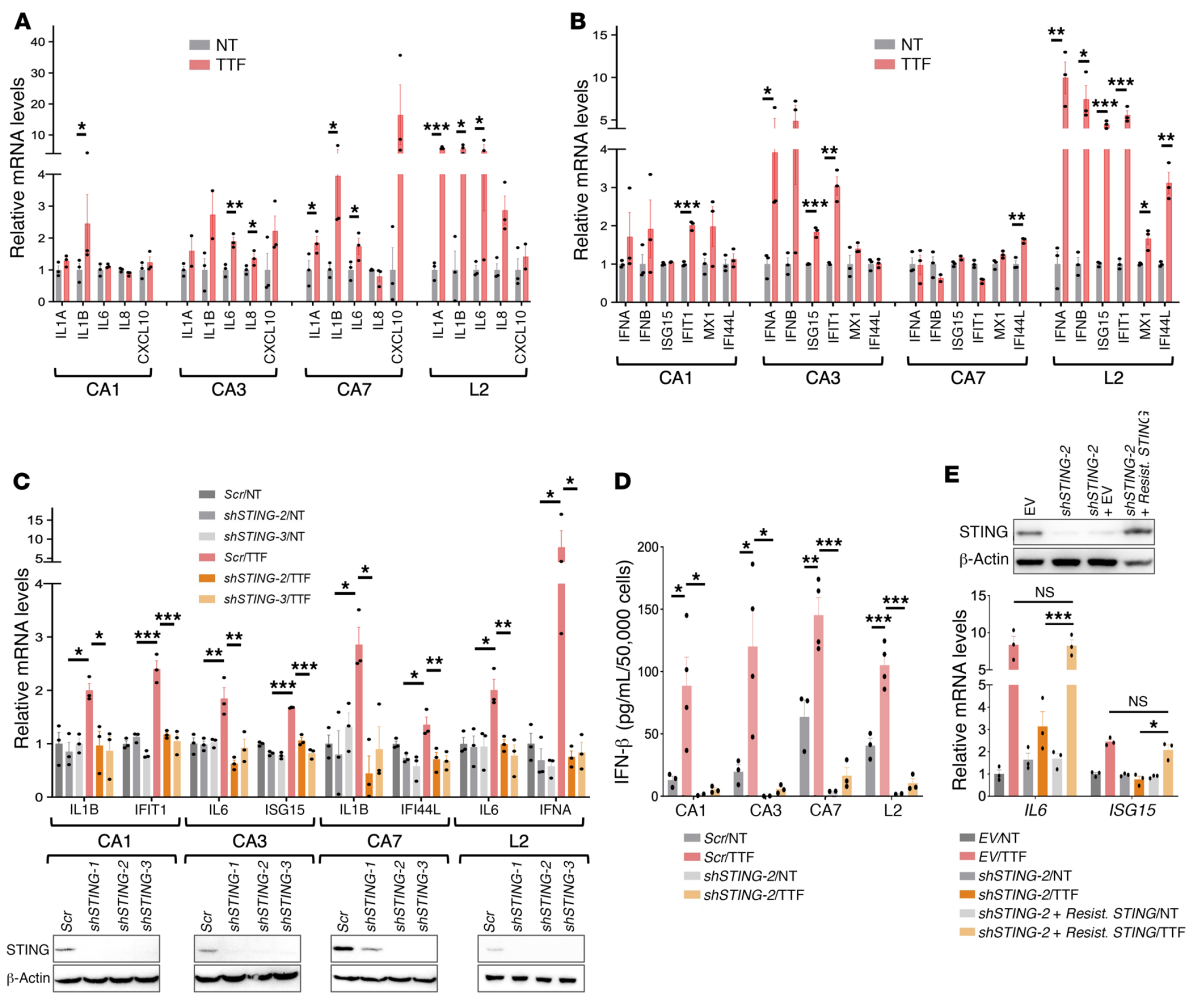
TTFields-activated cGAS/STING inflammasome induces PICs, T1IFNs, and T1IRGs in GSCs. (See Supplemental Figures 9 and 10). (**A** and **B**) Combination bar and dot plots demonstrating relative mRNA upregulation of indicated PICs (**A**) and T1IFNs/T1IRGs (**B**) after 24-hour treatment with TTFields in the 4 GSC lines. (**C** and **D**) Combination bar and dot plots showing that TTFields-induced upregulation of PICs and T1IFNs/T1IRGs was dependent on STING as measured in mRNA expression at 24 hours (**C**) and in IFN-β protein level in total lysate by ELISA at 72 hours (**D**) after TTFields treatment in the presence of *scrambled* (*Scr*) or 1 of the 2 independent *shSTING-1* and *shSTING-2* shRNAs. (**E**) A *shSTING-2*–resistant *STING* construct (*Resist*. *STING*) rescued *shSTING-2*–dependent suppression of TTFields-induced PICs and T1IFNs in CA3 GSCs, thus ruling out off-target effects of *shSTING-2*. All experiments used triplicate samples and were repeated at least 3 times. Data are represented as mean ± SEM. Analyses were performed using Student’s *t* test with a 2-tailed distribution for **A** and **B**, and 1-way ANOVA for **C**–**E**. *P < 0.05, **P < 0.01, ***P < 0.001.

STING, a signaling scaffold downstream of cGAS, recruits and activates TANK-binding serine/threonine kinase 1 (TBK1), which phosphorylates interferon (IFN) regulatory factor 3 at Ser396 (p-IRF3) and the NF-κB factor p65 at Ser536 (p-p65) (23), thereby driving them to the nucleus to upregulate proinflammatory cytokines (PICs), type 1 IFNs (T1IFNs), and T1IFN-responsive genes (T1IRGs). After 24 hours of TTFields, the p-IRF3 level increased in all 7 GBM lines, as did p-p65 in all 4 GSCs (Figure 2, A and B) and LN827 and U87MG cells (Supplemental Figure 8, A and B). To control for the general TTFields effects independently of the cGAS/STING pathway, we measured and consistently detected the conversion of LC3A/B-I to the autophagosome-associated LC3A/B-II in all TTFields-treated GSCs (11). In LN428 cells, despite having higher basal *STING* expression compared with U87MG and LN827 cells, p-p65 decreased while p-IRF3 increased after TTFields, coinciding with rapid STING downregulation (Supplemental Figure 8C). Although the mechanism of STING degradation under TTFields in GBM cells with high basal STING expression is unclear, in all 7 GBM lines, p-IRF3 and p65 were found concentrated in and around all observable TTFields-induced large micronuclei clusters (Figure 2C and Supplemental Figure 8, D–F). This coincided with upregulation of PIC (Figure 3A and Supplemental Figure 9A) and T1IFN/T1IRG mRNAs (Figure 3B and Supplemental Figure 9, B and C) and proteins (e.g., IFN-β; Figure 3D and Supplemental Figure 10D), which were reversed by 2 independent *STING* shRNAs to a similar extent (Figure 3, C and D, and Supplemental Figure 10). To further rule out off-target effects of *STING* shRNAs, we employed an *shSTING-2*–resistant wild-type *STING* construct and fully rescued the *shSTING-2*–dependent *IL6* and *ISG15* reduction in TTFields-treated CA3 GSCs (Figure 3E). Similar responses to TTFields were observed in A549 and PANC-1 cells (Supplemental Figure 4, B and D). Thus, TTFields activate the cGAS/STING inflammasome in GBM and other cancer cells, leading to increased production of PICs and T1IFNs in a *STING*-dependent manner.

**Figure 4:**
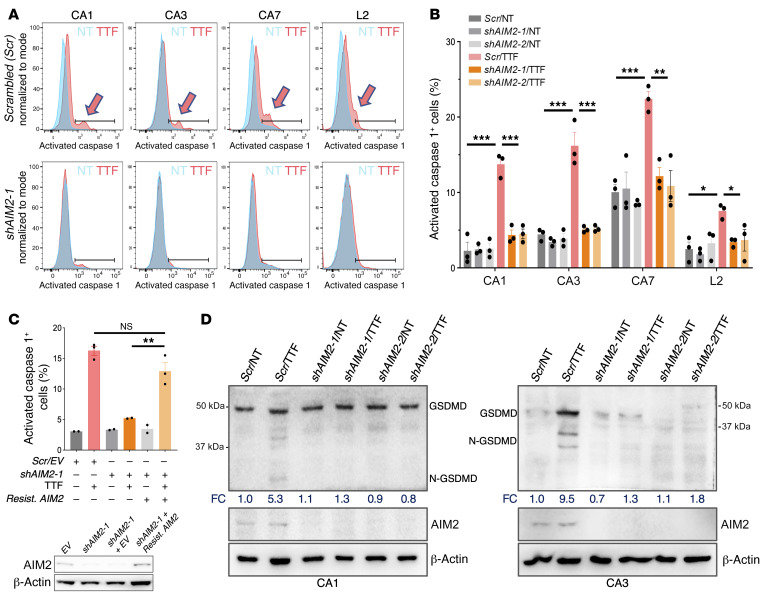
TTFields activate the AIM2/caspase 1 inflammasome in GSCs. (See Supplemental Figure 11). (**A** and **B**) Caspase 1 activation level following 24 hours of TTFields treatment, as measured by FAM-YVAD-FMK in the 4 GSC lines that expressed *scrambled* (*Scr*) or 1 of 2 independent *shAIM2-1* and *shAIM2-2* shRNAs (**A**) and summarized in a bar and dot graph (**B**). (**C**) A *shAIM2-1*–resistant *AIM2* construct (*Resist*. *AIM2*) rescued *shAIM2-1*–dependent suppression of TTFields-induced caspase 1 activation in CA1 GSCs, thus ruling out off-target effects of *shAIM2-1*. (**D**) Radiographs of immunoblotting for GSDMD showing the caspase 1–cleaved product (N-GSDMD) in total lysates from nontreated or TTFields-treated CA1 and CA3 GSCs expressing *Scr* or 1 of the 2 *AIM2* shRNAs. Shown is also fold change (FC) in density of the N-GSDMD relative to the full-length GSDMD and normalized to β-actin, with values for the nontreated *Scr* set to 1. All experiments used triplicate samples and were repeated at least 3 times. Data are represented as mean ± SEM. Analyses were performed using 1-way ANOVA. *P < 0.05; **P < 0.01; ***P < 0.001.

**Figure 5:**
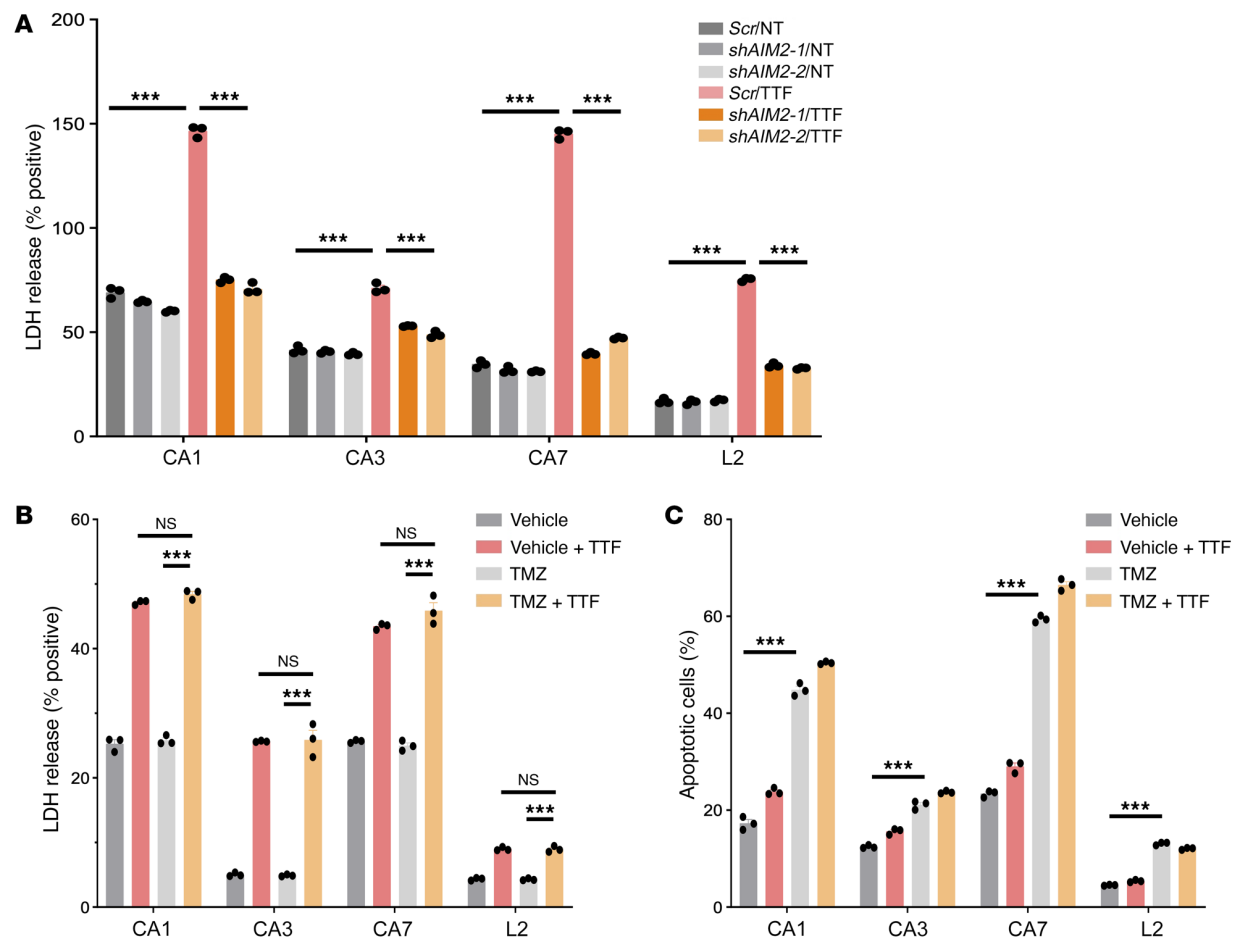
TTFields-activated AIM2/caspase 1 inflammasome induces membrane-damaged cell death in GSCs. (See Supplemental Figure 11). (**A**) A combination bar and dot plot of an LDH release assay showing TTFields-induced plasma membrane disruption in an *AIM2*-dependent manner following 24 hours of TTFields treatment in the presence of *scrambled* (*Scr*) or 1 of 2 independent *AIM2* shRNAs. (**B** and **C**) Combination bar and dot plots of an LDH release assay showing that TTFields-induced membrane-damaged cell death following 24 hours of TTFields treatment is distinct from apoptotic cell death caused by TMZ (150 μM for 24 hours) (**B**) as measured by annexin V membrane binding (**C**). All experiments used triplicate samples and were repeated at least 3 times. Data are represented as mean ± SEM. Analyses were performed using 1-way ANOVA. ***P < 0.001.

Next, to determine if TTFields activate the AIM2-dependent inflammasome in an *AIM2*-dependent manner, we utilized FAM-YVAD-FMK, a fluorescently labeled, specific, irreversible inhibitor of activated caspase 1, a key AIM2 target, to measure caspase 1 activation in response to TTFields in GBM cells with or without AIM2 depletion. In the 4 GSC lines, a new right-shifted peak of activated caspase 1 representing a 3- to 5-fold fractional increase in activated caspase 1–positive cells was consistently identified only in cells containing the scrambled shRNA and treated with TTFields, and not in those depleted of AIM2 using 2 independent *AIM2* shRNAs (Figure 4, A and B). In addition, expression of an *shAIM2-1*–resistant *AIM2* construct fully rescued the *shAIM2-1*–induced caspase 1 phenotype in TTFields-treated CA1 GSCs, thus ruling out off-target effects (Figure 4C). Similar results were observed in the 3 GBM cell lines (Supplemental Figure 11, A and B). Activated caspase 1 cleaves and releases PICs and the membrane pore-forming gasdermin D (GSDMD) (30), an executor of immunogenic programmed necrosis. We detected a 3- to 10-fold increase in the fraction of N-terminal cleavage product of GSDMD in response to TTFields in CA1, CA3 (Figure 4D), U87MG, and LN827 (Supplemental Figure 11C) cells in an *AIM2*-dependent manner. Of note, GSDMD expression was higher in TTFields-treated CA3 and U87MG cells, possibly contributing to a more robust production of the cleaved product observed in these cells. Although the mechanism behind this observation is unclear, GSDMD upregulation has been reported in response to T1IFNs (31, 32). GSDMD was not detectable by immunoblotting in CA7, L2, and LN428 cells under the same condition. Yet in all 7 GBM lines, there was a 2- to 5-fold *AIM2*-dependent increase in extracellular release of cytosolic lactate dehydrogenase (LDH) (24, 25) after 24 hours of TTFields treatment, indicating membrane-damaged cell death (Figure 5A and Supplemental Figure 11D). The increased LDH release associated with TTFields was specific to TTFields’ membrane-damage cell-killing effects and not due to secondary necrosis in late apoptosis (33) that can be induced by TTFields since the rate increases in LDH release after TTFields were disproportionately much higher than those in apoptosis induced by TTFields as measured by annexin V binding, especially in the 4 GSC lines, in which minimal to no increase in apoptosis was observed after TTFields (Figure 3, B and C). Moreover, apoptosis induced by 24-hour treatment with the cytotoxic drug temozolomide (TMZ, at 150 μM) was not associated with an increase in LDH release above those observed in the non-TTFields-treated or TTFields-treated cells (Figure 5, B and C, and Supplemental Figure 11, E and F). However, we cannot rule out a minor contribution from late apoptosis caused by TTFields to LDH release, especially in the GBM cell lines where TTFields-induced apoptosis was present.

In short, large cytosolic micronuclei clusters induced by TTFields recruit cGAS and AIM2 and activate their cognate inflammasomes, leading to upregulation of PICs, T1IFNs, and T1IRGs.

### TTFields-treated GBM cells provide a complete immunizing platform against GBM.

We turned to the 2 C57BL/6J-syngeneic orthotopic GBM models KR158 and GL261, which capture several clinicopathologic features of human GBM and represent a spectrum of poor and moderate immunogenicity and sensitivity to immunotherapy, respectively (34). cGAS/STING and AIM2/caspase 1 inflammasomes were activated by TTFields in luciferase-tagged KR158 cells (KR158-luc) and GL261 (GL261-luc) in a *STING*- and *AIM2*-dependent manner (Supplemental Figure 12), confirming that TTFields-induced activation of cytosolic DNA sensors and their cognate inflammasomes is conserved across cancer cell types and species.

**Figure 6:**
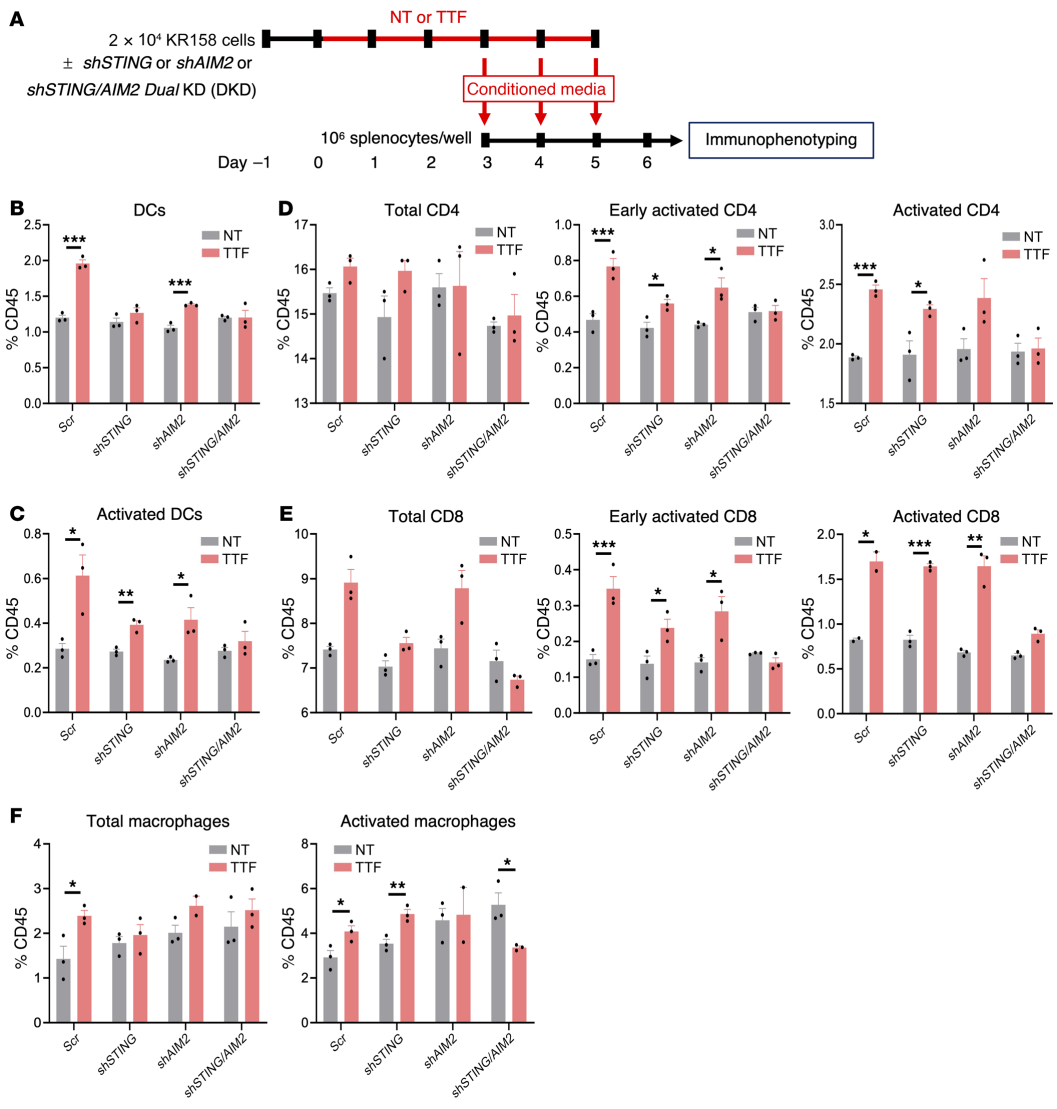
TTFields-induced PICs and T1IFNs stimulate DCs and lymphocytes. (See Supplemental Figure 12). (**A**) Schema of the coculture experiment. (**B**–**F**) Combination bar and dot plots showing immunophenotyping of all CD45^+^ cells in syngeneic splenocytes from C57BL/6J mice (*n =* 3) cocultured with conditioned supernatants obtained from KR158 cells with or without *scrambled* (*Scr*), individual *shSTING* or *shAIM2*, or dual *shSTING/AIM2* shRNAs that were either nontreated or treated with TTFields for 24 hours for the fractions of total DCs (MHCII^+^CD11C^+^) (**B**), activated DCs (CD80^+^CD86^+^) (**C**), total, early activated (CD69^+^) and fully activated (CD44^+^CD62L^–^) CD4^+^ (**D**) and CD8^+^ (**E**) T cells, and total (MHCII^+^CD11B^+^) and activated (F4/80^+^) macrophages (**F**). All experiments used triplicate samples and were repeated at least 3 times. Data are represented as mean ± SEM. Analyses were performed using Student’s *t* test with a 2-tailed distribution. *P < 0.05; **P < 0.01; ***P < 0.001.

To examine the effects of TTFields-induced PICs and T1IFNs on immune cells, we collected conditioned media from KR158-luc cells with or without TTFields treatment and shRNA knockdown of *STING* and *AIM2*, either individually or dually, to culture splenocytes isolated from healthy 6- to 8-week-old C57BL/6J mice for 3 days, and determined the fractions of T cells, DCs, and macrophages (Figure 6A). Total and activated (CD80/CD86^+^) DCs and the early activated (CD69^+^; ref. 35) and fully activated effector (CD44^+^CD62L^–^; ref. 36) fractions of CD4^+^ and CD8^+^ T cells increased with conditioned media from TTFields-treated KR158-luc when either STING or AIM2 was present, compared with media from nontreated cells and TTFields-treated cells with dual *STING/AIM2* depletion (Figure 6, B–E). Similar trends were also observed in total and activated macrophages but to a lesser degree (Figure 6F). Thus, PICs and T1IFNs induced by TTFields require either *STING* or *AIM2* and provide a potential link between TTFields and the adaptive immune system.

**Figure 7:**
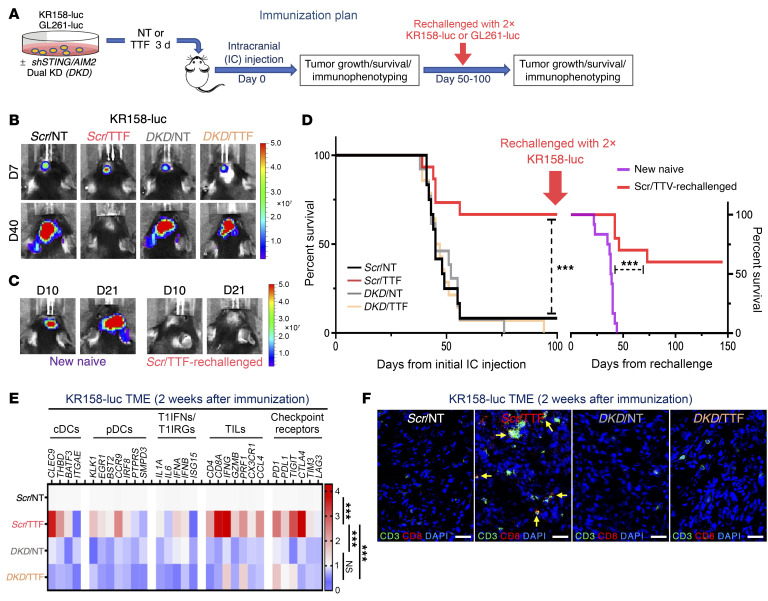
Induction of antitumor immunity in the KR158 syngeneic GBM model by TTFields requires STING and AIM2. (See Supplemental Figures 12–15). (**A**) Schema detailing the immunization and rechallenge protocol testing TTFields-treated murine GBM cells as a complete tumor cell–intrinsic immunizing platform. (**B**–**F**) Antitumor immunity in C57BL/6J mice induced by TTFields-treated KR158-luc cells. Representative photographs showing orthotopic GBM growth by BLI after immunization with *Scr*/NT (*n =* 12), *Scr*/TTF (*n =* 15), *DKD*/NT (*n =* 13), or *DKD*/TTF (*n =* 14) cells (**B**) and after rechallenge with twice the number of parental cells in the surviving *Scr*/TTF cohort (*n =* 10) and a new naive cohort (*n =* 12) (**C**). (**D**) Kaplan-Meier estimates showing survival rates after initial immunization and rechallenge and the immune TME summarized with a heatmap of a 29-immune-gene expression profile by qRT-PCR (*n =* 5 per cohort) (**E**) and representative images of IF for CD8, CD3, and DAPI counterstain (**F**). Scale bars: 50 μm. Log-rank test was used to compare survival rates and 2-way ANOVA to compare immune TME profile differences. ***P < 0.001. NS, not significant.

These results raise the prospect that TTFields-treated GBM cells may be harnessed to induce adaptive immunity against GBM. To test this concept of a tumor cell–intrinsic role in cancer immunization, we treated KR158-luc and GL261-luc cells in vitro first with TTFields for 72 hours based on the peak responses in human GBM cells (Supplemental Figure 9D), before implanting them into the right frontal cerebrum of C57BL/6J mice, thereby supplying both tumor-associated immunogens and adjuvant danger signals while also avoiding the confounding direct effects of TTFields on tumor stromal cells (Figure 7A). Importantly, we confirmed that the upregulation of PICs and T1IRGs in KR158-luc and GL261-luc cells persisted for at least 3 days after TTFields cessation, confirming the rationale for their use as a complete immunizing vehicle (Supplemental Figure 12, E and F). One animal cohort was immunophenotyped and their brains examined histologically 2 to 3 weeks after implantation and the rest monitored for tumor growth by bioluminescence imaging (BLI) and survival. To confirm an antitumor memory response, we rechallenged surviving animals with twice the number of KR158-luc and GL261-luc cells on day 100 and day 50 after immunization, respectively, based on differences in their tumor growth rates.

**Figure 8:**
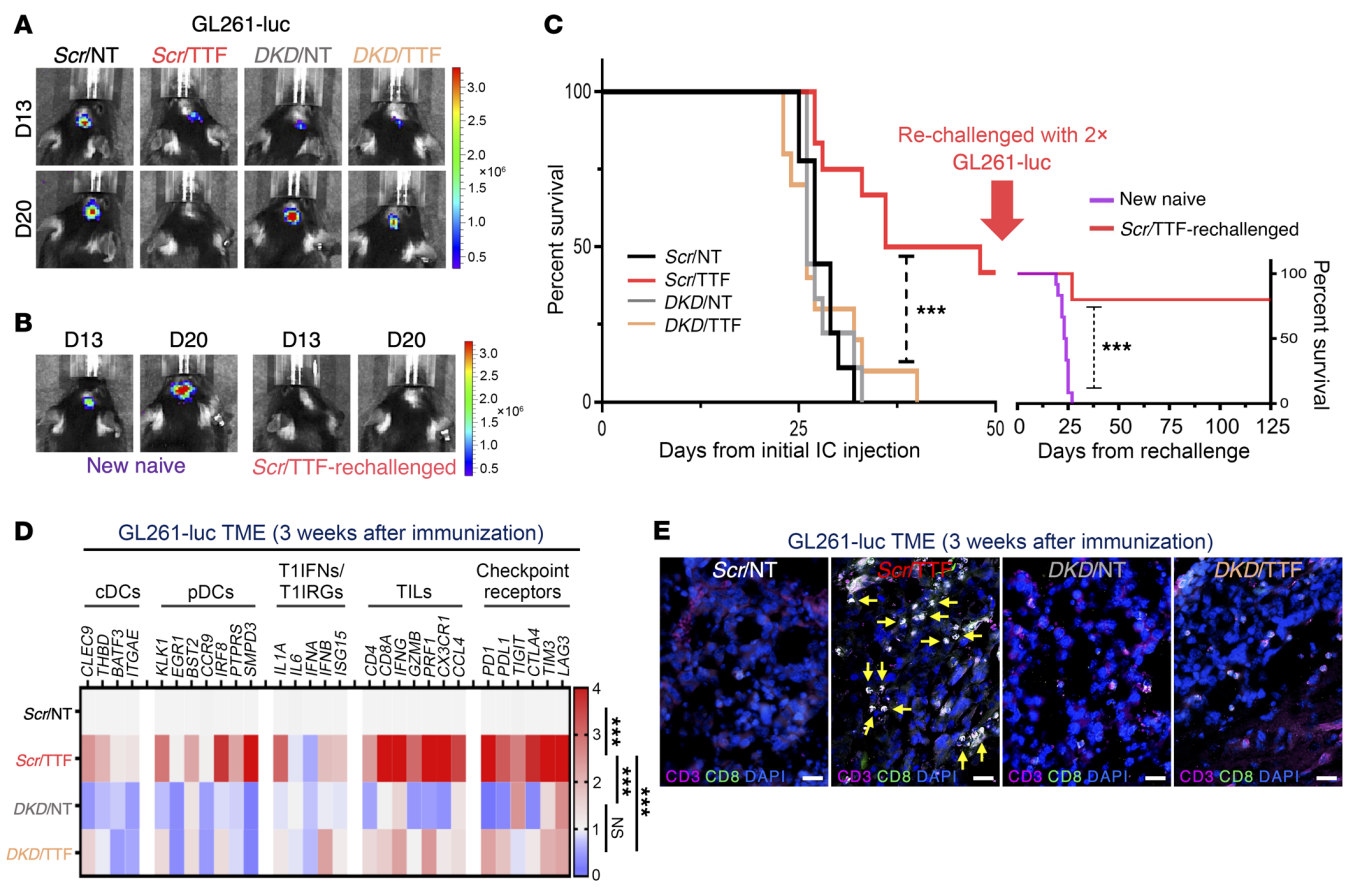
Induction of antitumor immunity in the GL261 syngeneic GBM model by TTFields requires STING and AIM2. (See Supplemental Figures 12–15). Antitumor immunity in C57BL/6J mice induced by TTFields-treated GL261-luc GBM cells. Representative photographs showing orthotopic GBM tumor growth by BLI after immunization with *Scr*/NT (*n =* 9), *Scr*/TTF (*n =* 12), *DKD*/NT (*n =* 9), or *DKD*/TTF (*n =* 10) cells (**A**) and after rechallenge with twice the number of parental cells in the surviving *Scr*/TTF cohort (*n =* 5) and a new naive cohort (*n =* 12) (**B**). (**C**) Kaplan-Meier estimates showing survival rates after initial immunization and rechallenge and the immune TME summarized with a heatmap of a 29-immune gene expression profile by qRT-PCR (*n =* 5 per cohort) (**D**) and representative images of IF for CD8, CD3, and DAPI counterstain (**E**). Scale bar: 50 μm. Log-rank test was used to compare survival rates and 2-way ANOVA to compare immune TME profile differences. ***P < 0.001. NS, not significant.

At day 7 and day 13 after implantation of KR158-luc and GL261-luc cells, respectively, all groups developed comparable BLI signals, confirming that primary tumor establishment was equivalent in all conditions. Subsequently, however, 38 of 39 (97%) and 28 of 28 (100%) animals collectively in the 3 control groups — *scrambled* shRNA/non-TTFields-treated (*Scr*/NT), dual *STING-AIM2* shRNA knockdown/TTFields-treated (*DKD*/TTF), and *DKD*/non-TTFields-treated (*DKD*/NT) — developed progressive brain tumors and succumbed by day 100 (median overall survival [mOS] of 45 days) in the KR158-luc model (Figure 7B and Table 1) and by day 40 (mOS of 27 days) in the GL261-luc model (Figure 8A and Table 2), respectively. In contrast, 10 of 15 (66%) and 5 of 12 (42%) animals immunized with *scrambled* shRNA/TTFields-treated (*Scr*/TTF) KR158-luc and GL261-luc cells had no detectable tumor on day 100 (mOS not reached) (Figure 7D and Table 1) and day 50 (mOS of 47 days) (Figure 8C and Table 2), respectively. When the surviving *Scr*/TTF-immunized mice from both models were rechallenged with twice the number of the corresponding parental GBM cells, 6 of 10 (60%) KR158-luc (Figure 7, C and D, and Table 1) and 4 of 5 (80%) GL261-luc (Figure 8, B and C, and Table 2) mice survived for at least 140 and 125 more days without detectable tumors, respectively, as compared with none of the 12 naive controls receiving either of the same parental cells surviving past 45 and 27 days, respectively. The *Scr*/TTF-immunized mice that succumbed after the rechallenge still exhibited improved mOS compared with the naive controls. Thus, 66% of KR158-luc and 42% of GL261-luc animals developed antitumor immunity and were cured of their GBM tumors in a TTFields-, *STING*-, and *AIM2*-dependent manner. Of these long-term *Scr*/TTF-immunized survivors, 60% and 80% animals, respectively, acquired antitumor memory immunity. An additional 25% and 20%, respectively, of the surviving *Scr*/TTF-immunized mice that were rechallenged derived partial immunity compared with the naive controls. Taken together, these results represent a remarkable feat for TTFields for its robustness and utility in inducing protective immunity in both poorly and moderately immunogenic GBM models.

To determine the immunological basis of these positive clinical observations, we profiled the immune TME 2 and 3 weeks after immunization in the KR158-luc (Figure 7E) and GL261-luc (Figure 8D) models, respectively, for transcripts of 29 key markers encompassing the innate and adaptive immune systems by quantitative reverse transcriptase PCR (qRT-PCR). In support of TTFields treatment turning the “cold” TME of GBM tumors “hot” in a *STING-* and *AIM2*-dependent manner through a T1IFN-based trajectory, we detected specifically in *Scr*/TTF tumors in both GBM models concomitant increases in markers of the PIC/T1IFN pathway, DCs, both classical (cDCs) and especially plasmacytoid (pDCs), a specialized DC subtype that is a direct target and the highest producer among DC subtypes of T1IFNs and key in linking the innate to adaptive immune systems (37), and TIL and CTL recruitment and activation (*IFNG*, granzyme B [*GZMB*], perforin 1 [*PRF1*], *CX3CR1*, and *CCL4*) (38–40). The CTL infiltration specifically in *Scr*/TTF tumors was confirmed by immunostaining of tumor sections for CD3^+^ and CD8^+^ T cells (Figure 7F and Figure 8E). Notably, several immune checkpoint receptors were also upregulated to varying degrees in *Scr*/TTF tumors relative to the 3 controls in both models.

**Figure 9:**
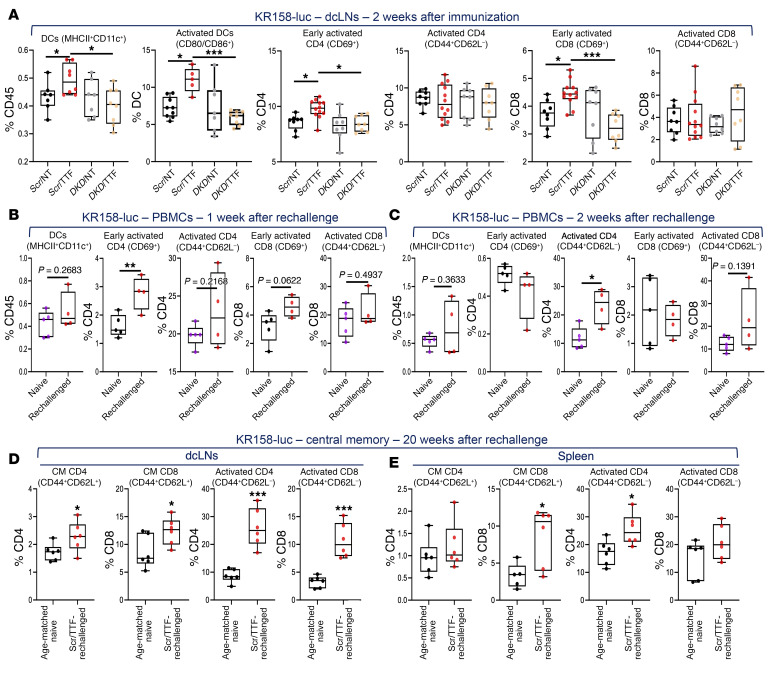
Immunophenotyping of TTFields-induced antitumor immunity in the KR158 GBM model. (See Supplemental Figure 13). (**A**) Combination box-and-whisker and dot plots showing immunophenotyping of C57BL/6J mice immunized with KR158-luc in various conditions in Figure 7 for total DCs and the fractions of activated DCs, early and fully activated CD4^+^ and CD8^+^ T cells in dcLNs 2 weeks after immunization (*n =* 7–12 mice for each cohort). (**B** and **C**) Combination box-and-whisker and dot plots showing immunophenotyping for the fractions of total DCs and early and fully activated CD4^+^ and CD8^+^ T cells in PBMCs of surviving *Scr*/TTF KR158-luc–immunized animals 1 (**B**) and 2 (**C**) weeks after rechallenge with twice the number of parental KR158 cells as compared with a new naive cohort implanted with the same cells (*n =* 5 for naive and *n =* 4 for *Scr*/TTF-rechallenged). (**D** and **E**) Combination box-and-whisker and dot plots showing the fractions of central memory (CM) CD4^+^ and CD8^+^ T cells and their activated (effector) counterparts in dcLNs (**D**) and splenocytes (**E**) in long-term-surviving *Scr*/TTF KR158-luc–immunized animals 20 weeks after rechallenge as compared with age-matched, sex-matched naive mice implanted with the same KR158-luc cells for 2 weeks (*n =* 6 each for naive and *Scr*/TTF-rechallenged). Data are represented as mean ± SEM. The whiskers are the minimum and maximum values, the lower and upper box edges the 25th and 75th percentage values, respectively, and the lines within the boxes the median. Comparisons were performed using 1-way ANOVA for **A** and Student’s *t* test with a 2-tailed distribution for **B**–**E**. *P < 0.05; **P < 0.01; ***P < 0.001.

**Figure 10:**
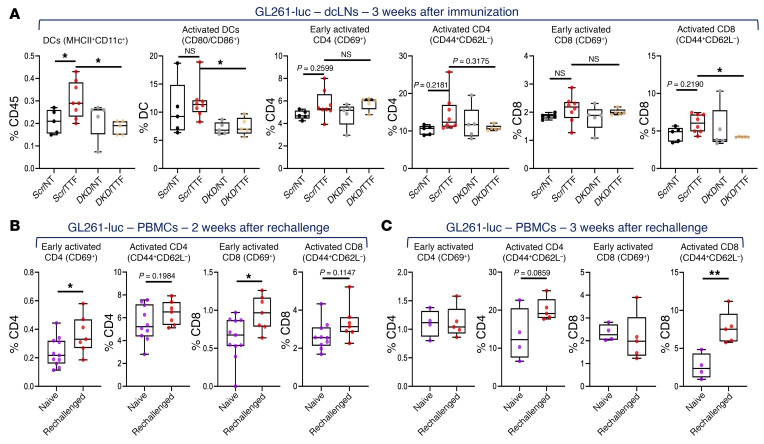
Immunophenotyping of TTFields-induced antitumor immunity in the GL261 GBM model. (See Supplemental Figure 14). (**A**) Combination box-and-whisker and dot plots showing immunophenotyping of C57BL/6J mice immunized with GL261-luc in various conditions in Figure 8 for total DCs and the fractions of activated DCs, early and fully activated CD4^+^ and CD8^+^ T cells in dcLNs 3 weeks after immunization (*n =* 8 for *Scr*/TTF and *n =* 5 for the other 3 cohorts). (**B** and **C**) Combination box-and-whisker and dot plots showing immunophenotyping for the fractions of early and fully activated CD4^+^ and CD8^+^ T cells in PBMCs of surviving *Scr*/TTF GL261-luc–immunized animals 2 (**B**) and 3 (**C**) weeks after rechallenge with twice the number of parental GL261 cells as compared with a new naive cohort implanted with the same cells (for 2 weeks, *n =* 11 for naive and *n =* 7 for *Scr*/TTF-rechallenged; for 3 weeks, *n =* 4 for naive and *n =* 5 for *Scr*/TTF-rechallenged). Data are represented as mean ± SEM. The whiskers are the minimum and maximum values, the lower and upper box edges the 25th and 75th percentage values, respectively, and the lines within the boxes the median. Comparisons were performed using 1-way ANOVA for **A** and Student’s *t* test with a 2-tailed distribution for **B** and **C**. *P < 0.05; **P < 0.01.

Next, we sought to define the cascade of systemic immunological events in these same animals, starting with the ipsilateral deep cervical lymph nodes (dcLNs), thought to directly drain the ipsilateral head and neck (41). Due to the low frequency of peripheral DCs and small sample volumes, we did not distinguish between the different DC subtypes in the subsequent analyses. In both GBM models, compared with animals receiving control cells, the fraction of all DCs in dcLNs increased in mice immunized with *Scr*/TTF cells, which was reversed when *DKD*/TTF cells were implanted (Figure 9A and Figure 10A). *DKD*/NT cells resulted in no difference in DCs in dcLNs compared to *Scr*/NT cells in both models, indicating that STING and AIM2 only became dominant with TTFields treatment. Importantly, of the DCs in dcLNs, the fraction of activated DCs (CD80/CD86^+^) also increased when *Scr*/TTF cells were implanted instead of control cells, which coincided with an increase or a trend of increase in the fractions of activated CD4^+^ and CD8^+^ T cells (either early [CD69^+^] or fully activated [CD44^+^CD62L^–^], or both), even though the total CD4^+^ and CD8^+^ fractions had not increased yet by this time (Figure 9A, Figure 10A, Supplemental Figure 13A, and Supplemental Figure 14A).

To assess for peripheral memory responses to KR158 and GL261 tumors, we performed serial immunophenotyping in splenocytes (both models) and PBMCs (KR158-luc only) within 2 to 3 weeks after primary immunization, although we expected minimal impact at this early time point, and then in PBMCs after rechallenge in both models. In KR158-luc animals, at week 2 after immunization, there was only a weak trend of increase in DCs and no change in lymphocytes in PBMCs, as expected, except that CD8^+^ T cells were higher in *Scr*/TTF mice (Supplemental Figure 13, B and C). Remarkably, however, in splenocytes we uncovered an increase in total and activated DCs and a trend of increase in CD69^+^CD8^+^ T cells in *Scr*/TTF animals, compared with controls at this early time point (Supplemental Figure 13, D–E), attesting to the vigor of TTFields-induced immune stimulation. Upon rechallenge, fractions of DCs and fully activated CD4^+^ and CD8^+^ T cells rapidly expanded at week 1 and rose further at week 2, while CD69^+^CD4^+^ and CD8^+^ T cells increased or trended toward increase only at week 1 in the rechallenged *Scr*/TTF KR158-luc cohort as compared with the vaccine-naive controls (Figure 9, B and C). To confirm the presence of durable central memory (CM), we measured the fractions of CM (CD44^+^CD62L^+^) CD4^+^ and CD8^+^ T cells (36) in the dcLNs and spleens 20 weeks after rechallenge in the 6 long-term-surviving rechallenged *Scr*/TTF KR158-luc mice. For controls, we orthotopically implanted the same number of KR158-luc cells into an age- and sex-matched cohort of 6 naive C57BL/6J mice and analyzed their dcLNs and spleens 2 weeks later. CM and effector (CD44^+^CD62L^–^; ref. 36) T cell fractions were consistently higher in *Scr*/TTF mice than in the naive controls (Figure 9, D and E). In the GL261 model, similar findings in the DC and lymphocyte compartments were observed in splenocytes and PBMCs isolated after immunization and after rechallenge, respectively, except that there was a 1-week delay in both time frames compared with KR158 mice (Figure 10, B and C, and Supplemental Figure 14, B and C). Of note, no differences in MDSCs and macrophages were seen in all cohorts at any time point in both models (Supplemental Figure 13, A and D, and Supplemental Figure 14, A and B).

Lastly, to rule out the possibility that programmed cell death other than DNA sensor–induced immunogenic cell death contributes to the clinical and immunological findings in *Scr*/TTF animals, we again turned to TMZ treatment, either alone or combined with TTFields (Supplemental Figure 15A). While TMZ at 300 μM for 72 hours caused cytotoxicity comparable to TTFields in KR158-luc cells, it had negligible effects on the cGAS/STING and AIM2/caspase 1 inflammasomes and contributed minimal survival advantage to the vehicle- or TTFields-treated controls (Supplemental Figure 15, B–D). Immunologically, TMZ did not increase DC and T cell activation compared to the vehicle controls, nor did its addition affect the adaptive immune activation induced by TTFields (Supplemental Figure 15, E and F).

In summary, TTFields generate large cytosolic micronuclei clusters via focal nuclear envelope disruption in GBM cells, thereby vigorously recruiting and activating the cGAS/STING and AIM2/caspase 1 inflammasomes to provide danger signals as well as immunogens to generate antitumor immunity against GBM tumors.

### Adaptive immune activation by TTFields in GBM patients via a T1IFN-based trajectory.

**Figure 11:**
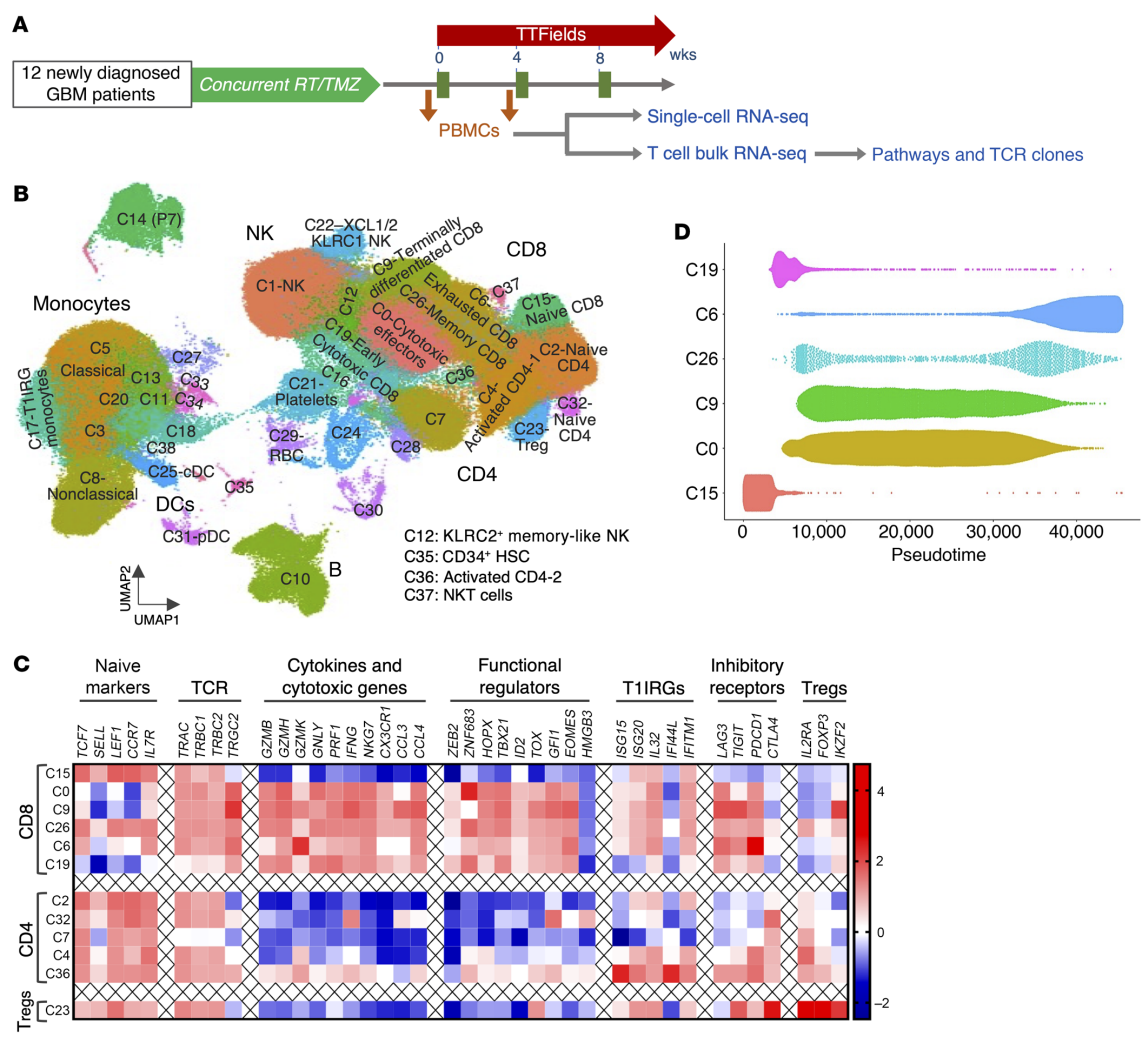
Single-cell and bulk RNA-seq of PBMCs in patients with newly diagnosed GBM treated with TTFields. (**A**) A diagram detailing adjuvant TTFields treatment in 12 patients with newly diagnosed GBM and the 2 analytical plans for PBMCs. (See Supplemental Tables 1–4 and Supplemental Figures 16 and 17). (**B**) A colored cell cluster map at resolution 1 using UMAP with 38 major immune cell types and subtypes of 193,760 PBMCs in 12 GBM patients. (See Supplemental Figures 18 and 19). (**C**) A heatmap of expression levels of the indicated gene set implicated in various T cell differentiation and functions providing the basis for annotations of the indicated major T cell clusters. (**D**) A graph showing pseudotime reconstruction of CD8^+^ T cell differentiation progression based on clusters in **B**.

The compelling observations in the KR158 and GL261 models led us to hypothesize that TTFields similarly activate adaptive immunity in patients with GBM, specifically through a T1IFN- and T1IRG-based trajectory, and that a gene signature linking TTFields to adaptive immunity is identifiable. To that end, we collected PBMCs from 12 adult patients with newly diagnosed GBM at least 3 weeks after they had completed radiation with concurrent TMZ at the following 2 times — within 2 weeks before and about 4 weeks after initiation of TTFields and maintenance TMZ (Figure 11A) — to perform (a) single-cell RNA sequencing (scRNA-seq) to identify the cell types and subtypes responsible for TTFields effects and (b) deep bulk RNA-seq of isolated T cells to identify a gene signature that captures broad effects of TTFields-induced T1IFNs across T cell subtypes. The high sequencing depth also enabled a focused clonal analysis of the most abundant T cell receptor (TCR) clones to provide direct evidence of adaptive immune activation by TTFields. PBMC viability and sequencing output for scRNA-seq and bulk RNA-seq are shown in Supplemental Tables 1–3, respectively. Patients’ basic characteristics are shown in Table 3. TTFields usage levels were high, with a median compliance of 86% of total time over the 4-week period (range 50%–96%). Dexamethasone doses were low at no more than 4 mg daily. Field delivery to tumor regions and transducer array layouts and placement were performed using the approved clinical NovoTAL mapping system based on individual patients’ head geometry and the lesion’s location, size, and shape on brain MRI (42). Gross tumor volume (GTV) was defined as the enhancing tumor including its necrotic core or the resection cavity plus the 3-mm peritumor boundary zone margin (PBZ3). Local minimum field intensity (LMiFI) and local average field intensity (LAFI), defined as the lower and average, respectively, of the 2 field intensities delivered to each point in the brain, and local minimum power density (LMiPD), defined as the product of field intensity and tissue-specific conductivities, were calculated using the finite element method as previously described (43). Models were successfully generated for 9 of 12 patients, showing comparable values of local field intensity and power density delivery to the GTV across all patients (Supplemental Figures 16 and 17 and Supplemental Table 4). Patient 28’s (P28’s) planning brain MRI was of insufficient quality to produce reliable measurements, while P12’s and P22’s recorded log files contained anomalies of unclear etiology.

In total, 193,760 PBMCs were resolved in the 24 samples, using the graph-based clustering technique in the Seurat R package (44, 45) and UMAP (46) for dimension reduction with increasing resolution parameter values (0.1, 0.3, 1, 3, 5, and 10). Resolution 1 was chosen, as it produced reasonably sized clusters, segregating PBMCs into 38 biologically recognized cell subtypes (Figure 11B). Cluster 14 (C14) exclusively in P7 contained an altered monocyte population of unclear significance. To annotate T cell clusters more accurately, we assembled a gene set consisting of cell-type markers and functional regulators, gleaned from the UMAP clusters and literature (refs. 47–50 and Figure 11C). For instance, C15 and C0 contained naive CD8^+^ and cytotoxic effectors based on expression of naive T cell and cytotoxic and cytokine markers, respectively, and differed from terminally differentiated CD8^+^ effectors of C9 in that C0 expressed the cytotoxicity regulator *ZNF683* (51) and lacked the inhibitory factors *TIGIT* and *IKZF2* (52, 53) found in C9 (Figure 11C and Supplemental Figure 18, A and B). C26 was composed of memory CD8^+^ effectors defined by *GZMB* (54), *CCL3* (55), and *CCR7* (56) and diverged from exhausted CD8^+^ effectors in C6 with high *GZMK* (48) and inhibitory receptors (Figure 11C and Supplemental Figure 18, C and D). Gamma/delta CD8^+^ T cells occupied much of the minor cluster of C19 (Figure 11C). A pseudo-timeline of temporal CD8^+^ differentiation of these clusters further validated this annotation scheme (Figure 11D).

**Figure 12:**
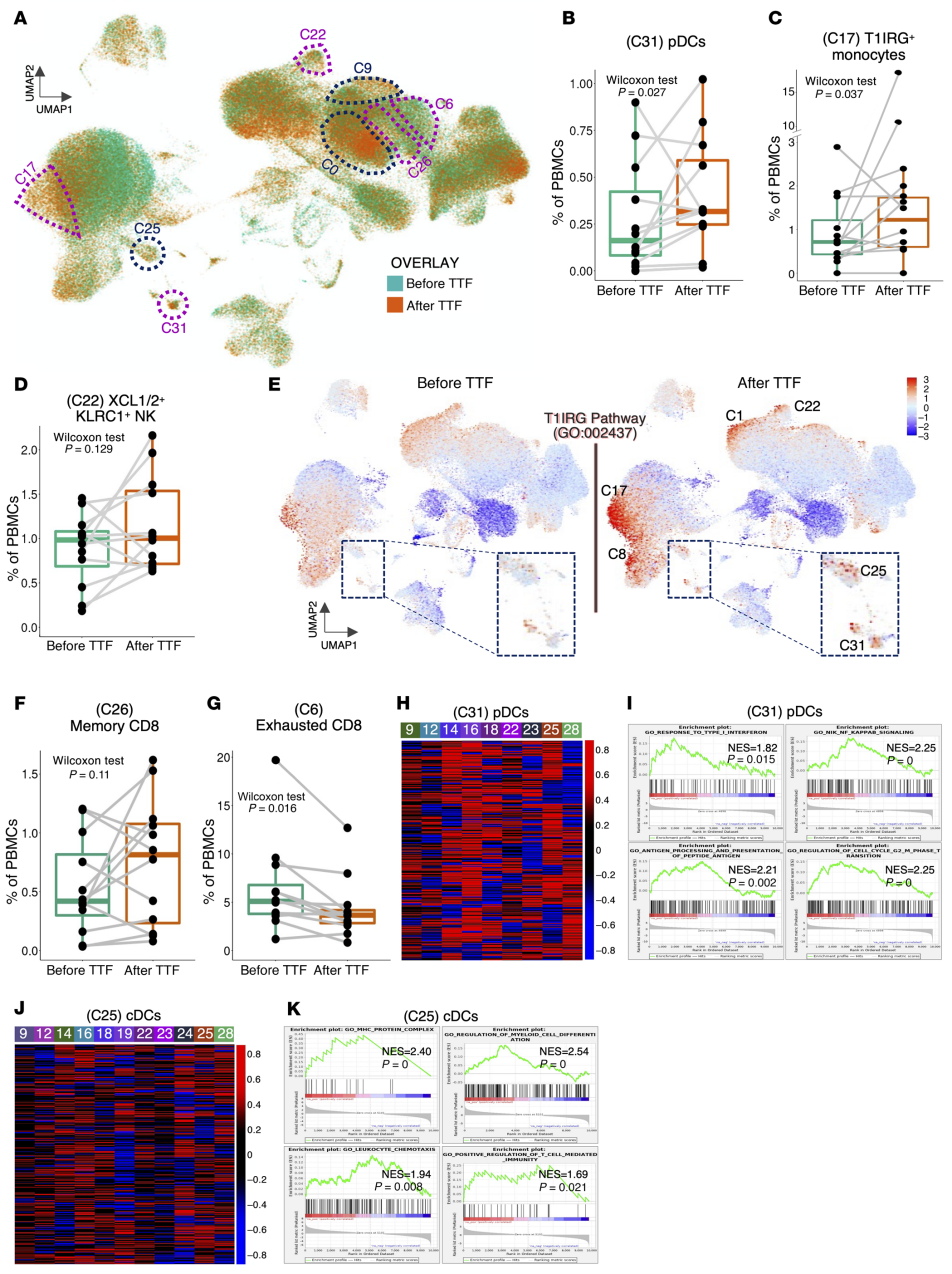
TTFields treatment correlates with immune activation via a T1IRG-based trajectory in GBM patients. (**A**) An overlay of pre-TTFields (pre-TTF, green) and post-TTF (orange) UMAP plots showing post-TTF changes. The purple broken lines denote clusters with both proportional and expression changes and the blue broken lines denote some of the clusters with expression changes only. (**B**–**D**, **F**, and **G**) Combination box-and-whisker and paired dot plots showing the proportions of the indicated clusters as percentages of total PBMCs in pre-TTF and post-TTF PBMCs in all 12 patients. Analysis was performed using Wilcoxon’s test. The whiskers are the minimum and maximum values, the lower and upper box edges the 25th and 75th percentage values, respectively, and the lines within the boxes the median. (See Supplemental Figure 22). (**E**) A heatmap of mean expression levels of the T1IRG pathway GO:0034340 at the single-cell, cluster-agnostic level in pre-TTF and post-TTF PBMCs in all 12 patients. (See Supplemental Figure S20A). (**H** and **J**) Heatmaps of gene expression showing logFC of expression of all genes in post-TTF compared with pre-TTF pDCs (*n =* 9) (**H**) and cDCs (*n =* 11) (**J**) in patients with detectable pre- and post-TTF counts. (See Supplemental Table 5 and Supplemental Figure 21). (**I** and **K**) GSEA of the indicated GO pathways in pDCs (**I**) and cDCs (**K**) in the same pre- and post-TTFields samples in **H** and **J**, respectively. NES, normalized enrichment score.

An overlay of the pre- and post-TTFields UMAP graphs revealed proportional increases in several clusters (Figure 12A). Consistent with TTFields inducing the immune system via a T1IFN-based trajectory and with the findings in the TME of immunized KR158 and GL261 models, we discovered in post-TTFields PBMCs higher proportions of pDCs (C31) (Figure 12B and Supplemental Figure 19A) and a monocyte subtype (C17) expressing T1IRGs (e.g., *IFI44L*, *MX1*, and *ISG15*) (Figure 12C and Supplemental Figure 19B). There was also a trend of increase in the *XCL1/2^+^KLRC1^+^* subtype (C22) of NK cells, another major T1IFN-responsive innate cell type (ref. 57 and Figure 12D, and Supplemental Figure 19C). To confirm that the 3 clusters constituted the backbone of the TTFields-induced T1IFN/T1IRG pathway, we conducted a pre- and post-TTFields global survey at the single-cell level in a cluster-agnostic fashion for the mean expression of the Gene Ontology–annotated GO:0034340, a major T1IRG pathway of 99 genes (58). For a negative control, we used GO:002437, a 73-gene non-T1IRG inflammatory pathway. After TTFields, the T1IRG pathway GO:0034340 formed an upregulated arc in the UMAP graph that specifically spanned these very 3 cell clusters (C31, pDCs; C17, T1IRG^+^ monocytes; and C22, *XCL1/2^+^KLRC1^+^* NK cells) and extended to other innate immune cell types, including nonclassical monocytes (C8), classical NK cells (C1), and cDCs (C25) (Figure 12E), compared with an unchanged scattered pattern with the non-T1IRG pathway GO:002437 (Supplemental Figure 20A). When gene coverage was expanded to all genes or cell-specific pathways using gene set enrichment analysis (GSEA; ref. 59), there was widespread expression upregulation in pDCs in all 9 patients with detectable pre- and post-TTFields pDCs specifically in T1IRG and DC regulatory pathways (Figure 12, H and I, and Supplemental Figure 21A). Moreover, post-TTFields pDCs upregulated the *IFNG* (T2IFN) pathway known to promote DC maturation (ref. 60 and Supplemental Table 5). Although no numerical increase was observed in cDCs, not unlike in the 2 mouse models where the cDC increases were noted mostly in dcLNs rather than in the blood, the cDC cluster (C25) (Supplemental Figure 19D) in 11 PBMCs exhibited pervasive post-TTFields upregulation, including the same pathways as in pDCs (Figure 12, J and K, and Supplemental Figure 21B). Likewise, TTFields treatment led to global upregulation in C17 and C22 (Supplemental Figure 21, C and F) and other immune clusters, albeit with higher interpatient variations (Supplemental Figure 21, D, E, G, and H). Taken together, these results confirmed robust post-TTFields gene upregulation in DCs and innate cells in GBM patients, specifically following a T1IRG-based trajectory.

Next, we asked whether TTFields-induced DC activation led to T cell activation, as noted in the KR158 and GL261 models. While cytotoxic (C0) and terminally differentiated (C9) effectors did not increase in proportion after TTFields, their expression profiles and that of activated CD4^+^ (C4) showed global upregulation to varying degrees across patients (Supplemental Figure 20, B and C, and Supplemental Figure 21, I–K) with specific enrichment in pathways linked to antigen-specific CD8^+^ T cell activation, e.g., antigen-binding, *NFKB* (61), cytokines (62), toll-like receptor 3 (63), and *FAS/FASL* (ref. 64 and Supplemental Figure 22, A and B). As predicted, terminally differentiated effectors (C9) also accentuated senescence and apoptotic pathways. The lack of proportional increase in cytotoxic effectors (C0) might be due to activation-induced cell death in late effectors promoted by *FAS/FASL* (64), as memory T cells emerged by 4 weeks after TTFields. In fact, there was a trend of proportional increase in memory CD8^+^ T cells (C26) (Figure 12F), concurring with a proportional decrease in exhausted effectors (C6) (Figure 12G) with both exhibiting global upregulation across patients (Supplemental Figure 21, L and M). GSEA of memory CD8^+^ T cells (C26) confirmed enrichment of regulatory pathways in memory T cell development, including *mTOR* (65), complement (66), and cell cycle checkpoints (Supplemental Figure 22C), whereas exhausted effectors (C6), besides activation pathways, upregulated those that induce T cell exhaustion-like apoptosis and negative regulation of the Hippo pathway (ref. 67 and Supplemental Figure 22D). In short, TTFields drive T cell activation toward memory development and away from exhaustion.

**Figure 13:**
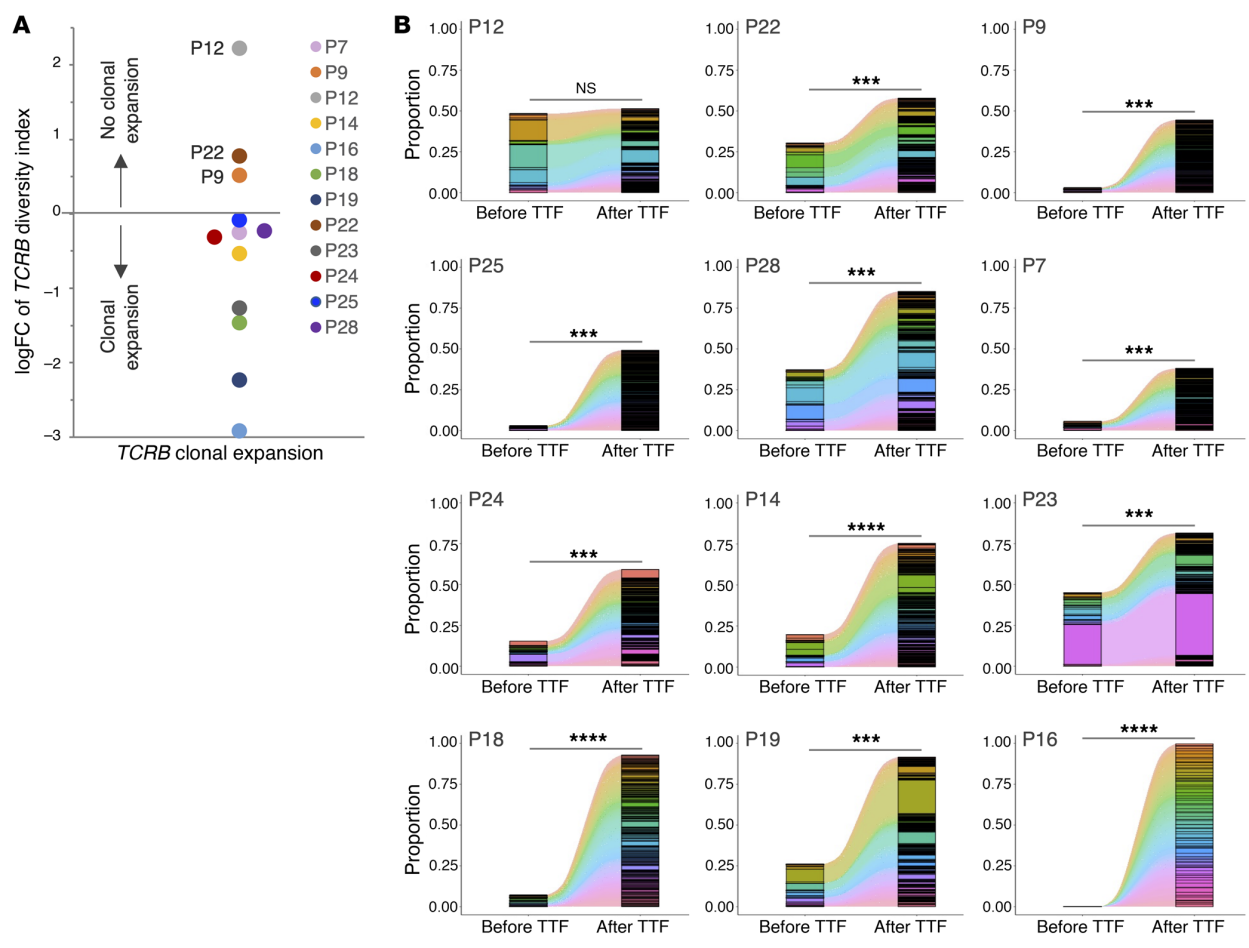
TTFields treatment correlates with *TCRB* clonal expansion in GBM patients. (See Supplemental Table 6 and Figure 23). (**A**) A dot plot of logFC of the Simpson diversity index (DI) of *TCRB* showing *TCRB* clonal expansion after TTFields treatment (negative DI logFC) in 9 of 12 patients. (**B**) 2D area charts of the 200 most abundant *TCRB* clones in post-TTFields T cells as compared with their proportions in pre-TTFields T cells showing clonal expansion in 11 of 12 patients. Student’s *t* test with a 2-tailed distribution was used for comparison. ***P < 0.001, ****P < 0.0001. NS, not significant.

Peripheral TCR clonal expansion, a hallmark of adaptive immunity (68), has been shown in several cancers to have high concordance with the tumor-infiltrating TCR repertoire, especially for the most abundant clones (69). Therefore, we extracted *TCRA*/*B* V(D)J sequences from the deep RNA-seq of T cells isolated from the same 12 PBMCs (Supplemental Table 6) to determine whether TTFields treatment affected TCR diversity, using the Simpson’s diversity index (DI), which is the average proportional abundance of TCR clones based on the weighted arithmetic mean (70). High and low DI values indicate even distribution and expansion, respectively, of TCR clones. Of the 12 patients, 9 exhibited negative log(fold change) (logFC) of *TCRB* DI after TTFields, indicating clonal expansion (Figure 13A). Notably, in all but 1 patient, the top 200 most abundant *TCRB* clones after TTFields, which accounted for 38.1% to 100% (median 67%) of detectable clones, showed substantial expansion compared to pre-TTFields T cells, and inversely correlated with the DI (Figure 13B). Similarly, *TCRA* also underwent post-TTFields clonal expansion in 9 of 12 patients, with the same patients at the 2 extremes of the DI scale (Supplemental Figure 23A), while all 12 patients uniformly expanded the top 200 clones (Supplemental Figure 23B). Thus, TTFields treatment is associated with adaptive immune activation as evidenced by clonal expansion of peripheral T cells.

**Figure 14:**
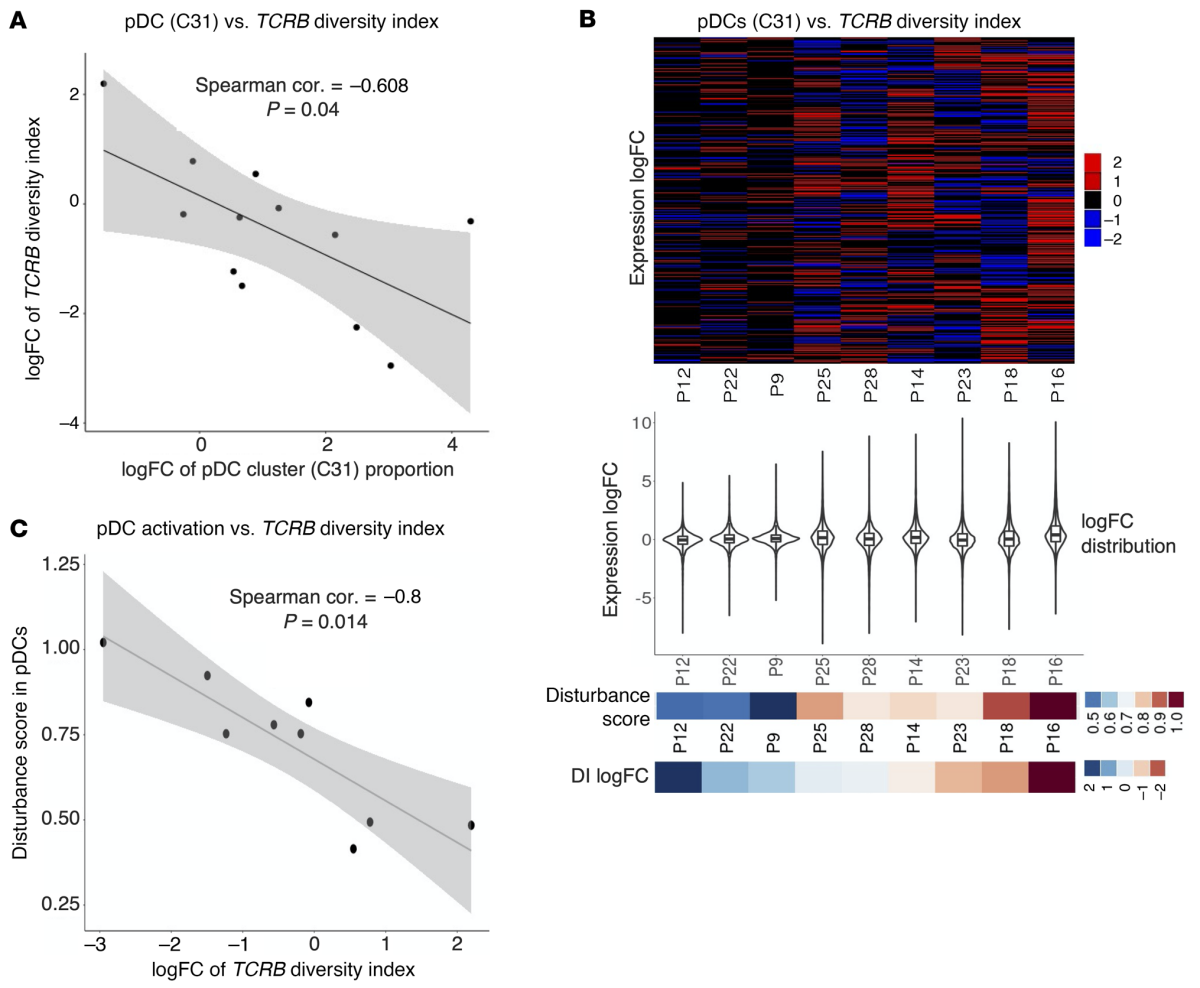
TTFields-induced *TCRB* clonal expansion correlates with pDC activation. (**A**) A scatter plot of logFC of DI versus logFC of proportion of cluster 31 (C31, pDCs) in all 12 patients showing a moderate negative correlation (Spearman’s correlation coefficient *r* = –0.608, *P =* 0.04). (**B** and **C**) Global gene expression disturbance after TTFields in pDCs (C31) strongly correlated with *TCRB* DI logFC in 9 patients who had detectable pre- and post-TTFields pDC counts. (**B**) Top: A heatmap of gene expression logFC between pre- and post-TTFields treatment. Middle: A violin plot of gene expression logFC distribution. Bottom: A heatmap of disturbance score, defined as mean of absolute gene expression logFC versus a heatmap of *TCRB* DI logFC ordered in decreasing DI logFC. (**C**) A scatter plot of *TCRB* DI logFC versus disturbance score showing a strong negative correlation (Spearman’s correlation coefficient *r* = –0.8, *P =* 0.014).

To confirm that the observed TCR clonal expansion reflects a tumor-specific response induced by TTFields rather than nonspecific reactions to the systemic inflammation created by TTFields-induced STING and AIM2 inflammasomes, we measured the strength of correlation between *TCRB* clonal expansion and pDCs. pDC proportion logFC was moderately negatively correlated with *TCRB* DI logFC in the 9 patients with a full pDC data set (Spearman’s coefficient *r* = –0.608, *P* = 0.04) (Figure 14A). To test whether this correlation became stronger at the molecular level of pDC activation measured by gene expression logFC distribution, we turned to the gene expression profiles of pDCs in these 9 patients. The 3 patients with positive DI logFC (P12, P22, and P9) segregated into a distinct group with gene expression logFC more concentrated near 0, i.e., less disturbed, compared with the other 6 patients whose gene expression logFC values were more widely distributed, i.e., globally disturbed (Figure 14B). A strong negative correlation between the disturbance score, defined as mean of absolute gene expression logFC across patients, and the DI logFC was observed (Spearman’s coefficient *r* = –0.8, *P* = 0.014) (Figure 14C), indicating that the TCR clonal expansion was likely a direct result of TTFields inducing adaptive immunity via pDCs.

**Figure 15:**
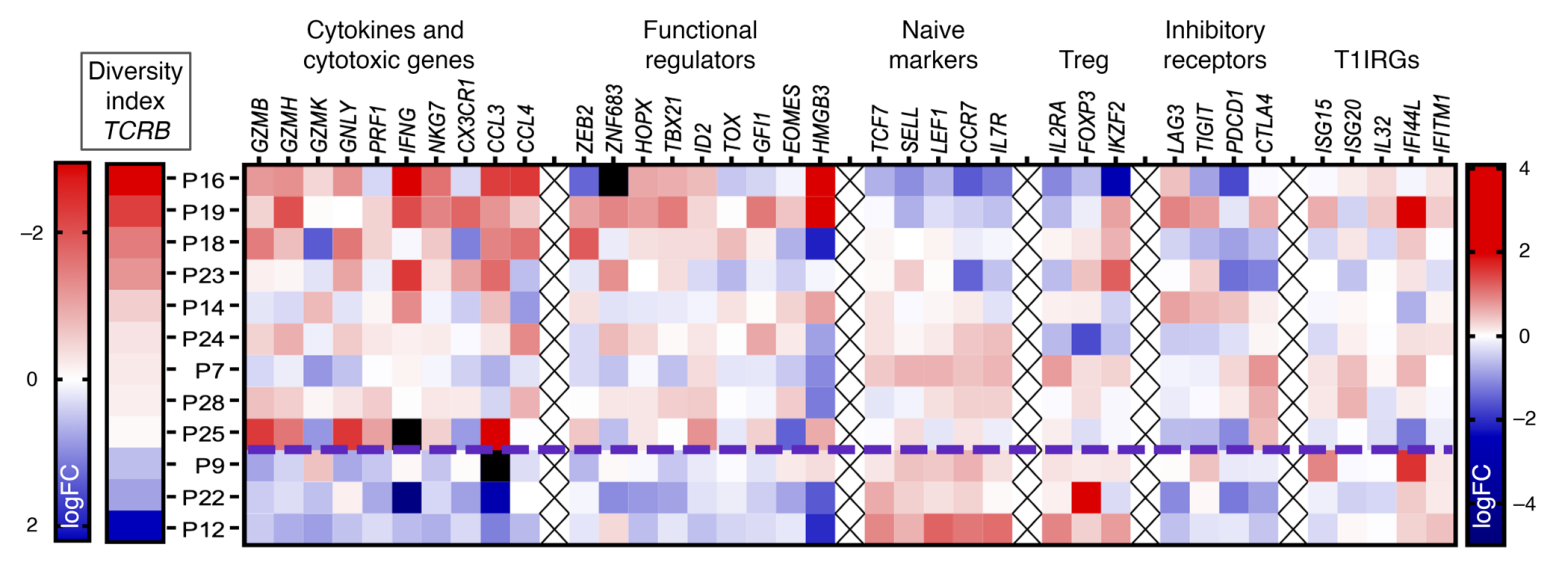
A gene panel signature of adaptive immune induction by TTFields in patients with GBM. A heatmap of gene expression of the same gene set used for T cell cluster annotations in the 12 patients ordered in increasing *TCRB* DI logFC showing a signature of adaptive immune induction by TTFields in patients with GBM.

Lastly, to define a gene panel signature of adaptive immune induction by TTFields, we took advantage of the gene set used to annotate T cell clusters (Figure 11C) to weigh against the *TCRB* DI logFC in all 12 patients (Figure 15). DI logFC was negatively correlated with levels of cytokine, cytotoxic, regulatory, and to a lesser degree, immune checkpoint genes, and positively correlated with naive and Treg markers, suggesting that the lack of *TCRB* clonal expansion in the 3 patients with positive DI logFC may be due in part to increased Treg activity. As expected, no correlation was observed between DI logFC and T1IRGs examined, further arguing against the post-TTFields *TCRA*/*B* clonal expansion being a nonspecific reaction to systemic inflammation.

Collectively, these results demonstrate that TTFields treatment leads to effective activation of adaptive immunity in patients with GBM, following the initial stimulation of immune cells along the T1IFN pathways, including pDCs and cDCs.

## Discussion

With the recent recognition of a critical role for cytosolic DNA sensors’ inflammasomes in stimulating antitumor immunity, the search for and development of pharmaceutical agonists of STING and AIM2 have been an active area of investigation in cancer immunotherapy (71). To that end, our compelling results place TTFields in a unique position as a dual and local activator of both inflammasomes without the systemic side effects of pharmaceutical agonists through its disruption of the nuclear envelope leading to cytosolic release of unprotected DNA, thereby creating a potentially complete tumor cell–intrinsic immunizing platform. For brain tumors, the use of TTFields has the added benefit of bypassing the BBB that can limit CNS delivery of pharmaceuticals. Equally important, this unique mechanism of action of TTFields may be generalizable and could be explored for immunotherapy in other tumors.

Although S-phase entry was necessary for TTFields-induced micronuclei clusters, affected cells were not in M phase, suggesting that TTFields-induced nuclear envelope disruption occurs during S and G_2_ phases. The nuclear envelope expands to accommodate increased DNA content by the end of S phase and, in the process, becomes weakened before total dissolution in prophase (72). This weakening may be accentuated in cancer cells, as their nuclear envelopes are less stiff (73), possibly rendering them more vulnerable to TTFields. To determine the precise timing and nature of TTFields-induced nuclear disruption would require high-resolution microscopy with or without targeted arrest at key cell cycle checkpoints. Regardless of the timing, the intense activation of the 2 inflammasomes in these large cytosolic micronuclei clusters, followed by PIC and T1IFN production, indicates that at least some of these clusters were transcriptionally active with most target genes present in them (Figures 2 and 3 and Supplemental Figures 8–10). However, low levels of nuclear translocation of p-IRF3 and p-p65 remain plausible, especially in cells with perinuclear distribution of the inflammasomes after TTFields, presumably due to nuclear envelope weakening.

Although we cannot rule out a direct destabilizing effect by TTFields on STING, the rapid STING degradation after TTFields observed in cells with high basal *STING* expression (e.g., LN428 and KR158) has been previously noted as a potential mechanism to prevent STING overstimulation (74). In fact, coinciding with the post-TTFields rapid STING degradation, LN428 cells exhibited higher cGAS recruitment to micronuclei clusters compared with U87MG and LN827 cells that have lower basal *STING* expression, and PICs, T1IFNs, and T1IRGs were robustly upregulated in both LN428 and KR158 cells. Our results support the T1IFN trajectory as the main conduit through which TTFields-activated STING-TBK1 complexes activate the innate immune system. Alternatively, TTFields-activated TBK1 may indirectly stimulate innate immunity through suppression of retinoblastoma-binding protein 5, recently shown to drive GSCs to evade innate immune signaling (75).

Since TTFields alone was sufficient to produce antitumor immunity in the 2 GBM models and TMZ cotreatment did not alter this property, we argue that the post-TTFields adaptive immune activation in GBM patients was more likely a direct response to TTFields rather than homeostatic proliferation that might occur after TMZ-induced lymphopenia. The homeostatic rebound was noted to be steeper for dose intense TMZ (100 mg/m^2^ daily for 21 days), which caused more severe lymphodepletion, compared with standard-dose TMZ (150 mg/m^2^ daily for 5 days; refs. 76, 77) employed in this study. In GBM and other solid tumors, homeostatic proliferation was shown to merely reconstitute the prechemotherapy T cell repertoire metrics (78). Notably, the sustained immunosuppressive effects of standard-dose TMZ were well documented in many tumors, including lymphopenia, an exhausted T cell state, and increased MDSCs and Tregs (79, 80), which are entirely opposite to the selective activation and expansion of pDCs, T1IFN-responsive NK and monocyte subtypes, memory T cells, and TCR clones, while restricting exhausted T cells as observed in TTFields-treated patients. However, we cannot rule out the possibility of a contribution by TMZ and/or delayed immunological evolution following radiotherapy and TMZ to the observed phenotypes in this patient cohort. Since TTFields is standard for GBM at many institutions, future studies could focus on comparing immune effects of TTFields plus TMZ to TTFields alone in MGMT-unmethylated GBM, which is resistant to TMZ (1) but not TTFields (81).

In patients, pDCs showed both proportional and activation increases after TTFields while only an activation increase was observed for cDCs in PBMCs, not unlike the 2 murine models, in which numerical increases in total DCs were observed primarily in dcLNs. Due to the difficulty with enumerating various DC subtypes in minute sample quantities from mice, it remained unclear whether pDCs also increased in PBMCs in mice. Nevertheless, we detected higher expression of markers for both cDCs and pDCs in the TME of both models after successful immunization. Overall, the response to TTFields by the DC compartment appeared conserved between the 2 species, attesting to the robust stimulatory signals induced by TTFields. From DCs, the stimulation flowed to adaptive immune effectors in humans with growing variations, likely reflecting interpatient differences in tumor-associated mutation burden and identity, dexamethasone doses, and genetic and epigenetic parameters among others that remain to be determined. Despite this, 9 of 12 patients exhibited TCR clonal expansion as measured by DI, and all but one had expanded the 200 most abundant *TCRA*/*B* clones. Although our method of extracting the *TCRA*/*B* repertoire from the deep bulk RNA-seq of isolated T cells for gene signature identification revealed only a fraction of the TCR diversity compared with the traditional target-specific sequencing method, this fraction likely comprised the most abundant clones that have been shown to have high concordance with tumor-infiltrating T cell clones (69). Without losing relevant information, this method is increasingly utilized for rare clinical samples for obvious practical and cost-saving reasons (82).

Finally, the successful post-TTFields reversal of local and systemic immunosuppression characterized by high infiltration of CTLs and immune checkpoint expression in the TME as well as robust systemic CTL activation, clonal expansion, and immune checkpoint upregulation in GBM patients provides a compelling rationale for combining TTFields with immune checkpoint inhibitors to create a potential therapeutic synergy. The gene signature for TTFields’ CTL effects (Figure 15) can be further refined in subsequent studies to predict and stratify responses in future TTFields-based immunotherapy.

## Methods

### Materials and methods.

Please refer to the supplemental materials for details.

### RNA-seq data deposit.

RNA-seq data generated in this study have been deposited in the NCBI’s Gene Expression Omnibus (GEO GSE19352 and GSE193729).

### Study approval.

Animal work and human subject work were performed accordingly to approved protocols from the IACUC and IRB, respectively, of the University of Florida. Written informed consent was obtained from each human participant before study procedure and analyses were performed.

## Author contributions

DC, SBL, and DDT conceived and performed experiments and wrote the manuscript. DDT performed the clinical study and secured funding. TEH, MS, DJ, AAC, and TL performed experiments. AG and MR performed the clinical study and provided clinical expertise and feedback.

## Supplementary Material

Supplemental data

## Figures and Tables

**Table 3:**
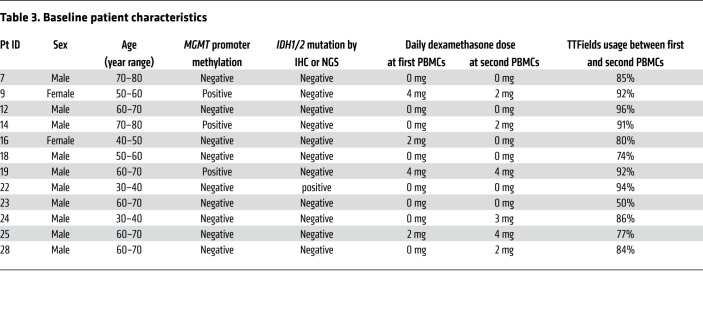
Baseline patient characteristics

**Table 2:**
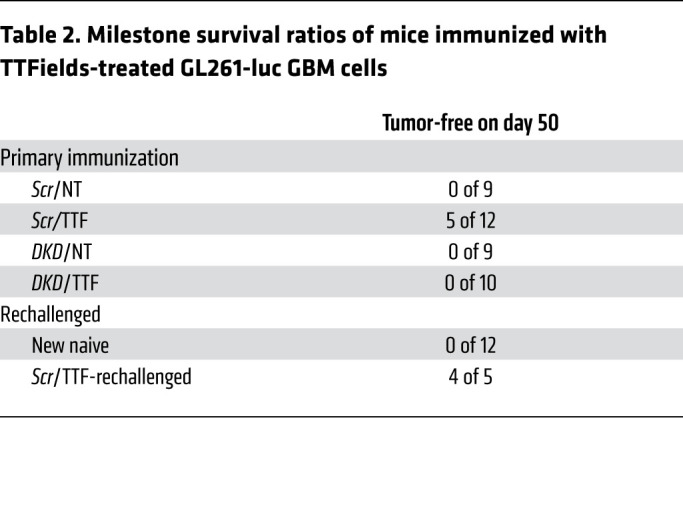
Milestone survival ratios of mice immunized with TTFields-treated GL261-luc GBM cells

**Table 1:**
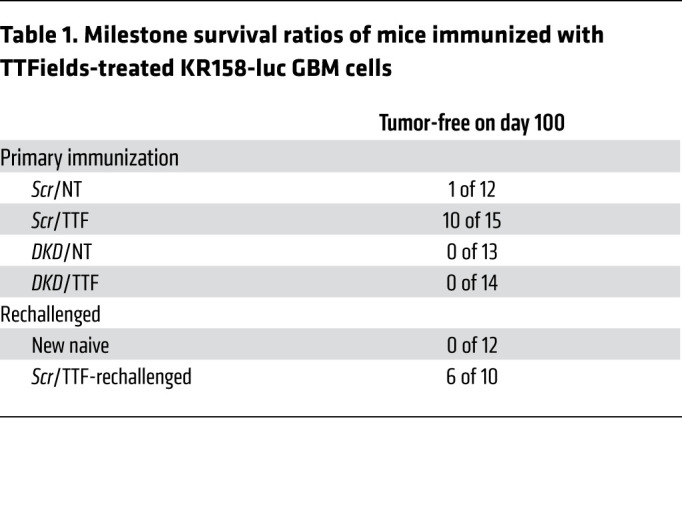
Milestone survival ratios of mice immunized with TTFields-treated KR158-luc GBM cells

## Version 1 02/24/2022

In-Press Preview

## Version 2 04/15/2022

Electronic publication
