# Supplementary material for: Tumor Treating Fields dually activate STING and AIM2 inflammasomes to induce adjuvant immunity in glioblastoma
Source: J Clin Invest. 2022 Apr 15;132(8):e149258. doi: 10.1172/JCI149258 (PMC9012294; doi:10.1172/JCI149258)

# SUPPLEMENTAL MATERIALS

# 149258-JCI-RG-RV-3

## Tumor Treating Fields Dually Activate STING and AIM2 Inflammasomes to Induce Adjuvant Immunity in Glioblastoma

**Chen et. al.**

**JCI**

# SUPPLEMENTAL METHODS

## Antibodies

For immunofluorescence and Western blotting: Primary antibodies - LAMIN A/C (Santa Cruz, Cat#sc-7292 and 376248-AF488), cGAS (Santa Cruz, Cat#sc-515802), STING (CST, Cat#13647), AIM2 (CST, Cat#12948; Proteintech, Cat#14357-1-AP), IRF3 (Santa Cruz, Cat#sc-33641), p-IRF3(CST, 29047S), p65 (Santa Cruz, Cat#sc-8008), p-p65 (Santa Cruz, Cat#sc-136548), GSDMD (SIGMA, Cat#G7422), caspase-1 (Santa Cruz, Cat#sc-514), ActinGreen 488 (Thermo Fisher, Cat#R37110), β-tubulin (Santa Cruz, Cat#sc-5274), and β-actin (Santa Cruz, Cat#sc-47778). Secondary antibodies - goat anti-mouse-A555 (Jackson Immunoresearch, Cat#111-295-003), goat anti-rabbit IgG-A647 (Jackson Immunoresearch, Cat#111-605-003), HRP-conjugated anti-mouse (Santa Cruz, Cat#sc-516102), and HRP-conjugated anti-rabbit (Enzo, Cat#ADI-SAB-300-J). For flow cytometry: Antibodies were purchased from Biolegend and diluted at 1:200 unless otherwise specified: CD45 (clone: 30-F11,Cat#103126, 103108, 103112), MHCII (clone: M5/114.15.2, Invitrogen, Cat#48-5321-32, 1:400), CD4 (clone: RM4-5, Invitrogen, Cat#47-0042-80), CD44 (clone: IM7, Cat#103012, 1:100), ly6g/ly6c (clone: RB6-8C5, Cat#108411), CD8α (clone: 53-6.7, Cat#100721), CD11b (clone: M1/70, Cat#101215), CD80 (clone: 16-10A1, Cat#104733), CD62L (clone: MEL-14, Cat#104405), CD86 (clone: GL-1, Cat#105005, 1:150), CD69 (clone: H1.2F3, Cat#104507), F4/80 (clone: BM8, Cat#123110), and CD11c (clone: N418, Cat#117307, 1:100).

## Cell Culture

The 4 patient-derived GSC lines CA1, CA3, CA7, and L2, previously generated accordingly to rules and regulations by the institutional review board (IRB) and described (1), were grown in stem cell media **(** STEMCELL, Cat#05750 with bFGF, EGF and heparin). HEK 293T (from ATCC) and human GBM cells U87MG (from ATTC) (2), LN428 (3), and LN827 (4), A549, and PANC-1 (from ATTC) were grown in DMEM media supplemented with 10% FBS and 1% pen/strep and the mouse GBM cell line KR158-luc (5) in RIPA 1640 media supplemented with 10% FBS and 1% pen/strep. To produce lentivirus, we used PEI (1µg/µl) at a 2:1 ratio of PEI (µg): total DNA in the pLKO.1 backbone (µg) to transfect HEK 293T cells. PSPAX2 and PMD2.G plasmids were used for viral packaging and enveloping, respectively in advanced DMEM media supplemented with 1.25% FBS, 10mM HEPES, 1X Pyruvate and 10mM Sodium butyrate. TTFields were applied to cancer cell lines using the Inovitro™ system (Novocure, Israel). Cells were treated with TTFields at frequencies of 200 kHz (CA1, CA3, CA7, L2, U87, LN827, LN428, KR158-luc, and GL261-luc) and 150 kHz (A549, PANC-1).

**Immunofluorescence and Confocal Microscopy**

Cells grown on cover slips were fixed with 4% paraformaldehyde for 30 min at 4°C, permeabilized with 0.1% cold Triton X-100 in PBS for 15 min at 4°C and blocked with 5% normal goat serum in PBS for 1 hr. at 4°C and incubated overnight at 4°C with different combinations of primary antibodies (dilution 1:500) against indicated antigens and then for 2 hrs. at room temperature with appropriate fluorochrome-conjugated secondary antibodies (dilution 1:500). Labeled cells were counterstained with DAPI (Thermo Fisher, Cat#D1306) at 1µg/ml for DNA content and actin cytoskeleton by ActinGreen (phalloidin AlexaFluor-488) for cytoplasmic outline, and images captured and analyzed, using a Zeiss 800 inverted confocal microscope. Images were captured at

63X oil immersion objective either at 0.5X or 1X zoom, keeping all the conditions of microscope, exposure and software settings identical for all samples. The Z-stack analysis was performed by obtaining 15 to 20 (1µm thickness each) optical sections for each microscopic field. The Arivis software was used to generate 3D microscopy image and to observe 360° panoramic view of microscopy field. For all other analyses, Zen software was used.

**Quantitative RT-PCR**

QIAGEN RNeasy Mini Kit (Cat#74106) was used to extract RNA from cells/tissues according to the manufacturer's protocol. One µg total RNA was subjected to reverse transcription using iScript cDNA Synthesis Kit (BIO-RAD, Cat#1708891). qPCR was performed using PowerUp SYBR Green Master Mix (Applied Biosystems, Cat#A25741) and on QuantStudio 3 from Applied Biosystems. Primers used are as follow: hISG15 forward (fw) GGTGGACAAATGCGACGAA, reverse (rev) TGCTGCGGCCCTTGTTAT; hCXCL10 fw AAGTGCTGCCGTCATTTTCT, rev CCTATGGCCCTCATTCTCAC; hSTING fw GCCAGCGGCTGTATATTCTC, rev GCTGTAAACCCGATCCTTGA; hIFNα fw GACTCCATCTTGGCTGTGA, rev TGATTTCTGCTCTGACAACCT; hIFNβ fw GAATGGGAGGCTTGAATACTGCCT, rev TAGCAAAGATGTTCTGGAGCATCTC; hGAPDH fw GGCATGGACTGTGGTCATGA, rev ACCACCATGGAGAAGGC; hIL1α fw TGTAAGCTATGGCCCACTCCA, rev AGAGACACAGATTGATCCATGCA; hIL1β fw CTCTCTCCTTTCAGGGCCAA, rev GAGAGGCCTGGCTCAACAAA; hIL6 fw CACCGGGAACGAAAGAGAAG, rev TCATAGCTGGGCTCCTGGAG; hIL8 fw ACATGACTTCCAAGCTGGCC, rev CAGAAATCAGGAAGGCTGCC; hAIM2 fw GCTGCACCAAAAGTCTCTCC, rev ACATCTCCTGCTTGCCTTCT; hIFIT1 fw TGAAGTGGACCCTGAAAACC; hIFIT1 rev

TAAAGCCATCCAGGCGATAG; hMX1 fw GGGAAGGAATGGGAATCAGT; hMX1 rev CCCACAGCCACTCTGGTTAT; hIFI44L fw GATGAGCAACTGGTGTGTCG; hIFI44L rev ACTGACGGTGGCCATAAAAC; mAIM2 fw CATGGAGGTCACCAGTTCCT, rev TTTGTTTTGCTTGGGTTTCC; mIFNβ fw CCCTATGGAGATGACGGAGA, rev CTGTCTGCTGGTGGAGTTCA; mIL6 fw CCGGAGAGGAGACTTCACAG, rev TCCACGATTTCCCAGAGAAC; mISG15 fw AAGAAGCAGATTGCCCAGAA, rev CGCTGCAGTTCTGTACCAC; mIFIT1 fw GCCCAGATCTACCTGGACAA; mIFIT1 rev CCTCACAGTCCATCTCAGCA; mMX1 fw TGTGCAGGCACTATGAGGAG; mMX1 rev ACTCTGGTCCCCAATGACAG; mIFI44L fw GGGGTCTGACGAAAGCAGTA; mIFI44L rev CCCATTGAATCACACAGCAT; mSTING fw GTTTGCCATGTCACAGGATG, rev CAATGAGGCGGCAGTTATTT; mGAPDH fw GGAGCGAGACCCCACTAACA, rev ACATACTCAGCACCGGCCTC. mCCR9 fw TGACTCCACTGCTTCCACAG, rev GTGCCCACAATGAACACAAG; mBATF3 fw TTTCGAAGCTGAAGGAGGAG, rev ACCGAAGCTGCACAAAGTTC; mITGAE fw GTCACTAAGACCGCCTCAGC, rev GGTCTGAGGCAATCAGCTTC; mKLK1 fw TTTGGAGGATGAACCCTCTG, rev TGATGTCAGCAGGCTTTTTG; mIFNG fw ACTGGCAAAAGGATGGTGAC, rev TGAGCTCATTGAATGCTTGG; mPDL1 fw TGCTGCATAATCAGCTACGG, rev GCTGGTCACATTGAGAAGCA; mPD1 fw GGAGCAGAGCTCGTGGTAAC, rev TACCAATGACCATGCCTTGA; mTIM3 fw TTGGAGTGGGAGTCTCTGCT, rev GGCAAGTTGGCCAGTGTAAT; mLAG3 fw TTTCCTGTTACTGCCCCAAG, rev CACTTGGCAATGAGCAAAGA; mTIGIT fw GCCTCACCTTCCAGTCTCTG, rev GCCACTGAGCTTTCTTGGAC; mCD4 fw AGGAAGTGAACCTGGTGGTG, rev CTCCTGCTTCAGGGTCAGTC; mCD8α fw TATGGCTTCATCCCACAACA, rev

GACTGGCACGACAGAACTGA; mCTLA4 fw CAGGTGACCCAACCTTCAGT, rev CAGTCCTTGGATGGTGAGGT; mCLEC9 fw GGAGCATGGTGTGTTGTGAC, rev GTCTTTCCTGCTGCTCCAAG; mTHBD fw CTTGTGCAATAGGAGCACGA, rev GACACAAAAATGCTCGCAGA; mEGR1 fw CCACAACAACAGGGAGACCT, rev ACTGAGTGGCGAAGGCTTTA; mBST2 fw CAATCTACTTCGCCGTCACA, rev TCTTCTCCAGGGACTCCTGA; mIFNα1 fw AGTGAGCTGACCCAGCAGAT, rev GGTGGAGGTCATTGCAGAAT; mIRF8 fw GATCGAACAGATCGACAGCA, rev GCTGGTTCAGCTTTGTCTCC; mPTPRS fw TAACATGCTCAGTGGCAAGC, rev TCCTCCAGATCCATGTCCTC; mSMPD3 fw CACCAACACCTCCATCAGTG, rev GTCAGCCTGTTCTCCAGAGG; mCX3CR1 fw CTTTGGGGGCATATTCTTCA, rev ACGCCCAGACTAATGGTGAC; mPRF1 fw GATGTGAACCCTAGGCCAGA, rev GGTTTTTGTACCAGGCGAAA; mGZMB fw TCGACCCTACATGGCCTTAC, rev TGGGGAATGCATTTTACCAT; mCCL4 fw CCCACTTCCTGCTGTTTCTC, rev GTCTGCCTCTTTTGGTCAGG.

**Western blotting**

Cells were treated on ice for 20 min with RIPA buffer (150mM NaCl, 1% NP-40, 0.5% Sodium deoxycholate, 0.1% SDS, 25mM, PH 7.4 Tris) containing a protease inhibitor cocktail (Roche), followed by centrifugation at 13,000g at 4 °C for 20 min. Supernatants were collected and protein concentration determined using a protein assay dye reagent (Bio-Rad). Equal amounts of proteins were resolved by SDS-PAGE and transferred onto polyvinylidene difluoride (PVDF) membranes. Membranes were blocked with 5% non-fat milk in TBST, then probed with indicated primary

antibodies (1:500) at 4 °C overnight, washed with TBST, and incubated with HRP-conjugated anti-rabbit or anti-mouse secondary antibodies (1:500) at room temperature for 1 hr.

**Flow cytometry**

Single cell suspension was Fc-blocked cells before incubated with indicated fluorochrome-conjugated antibodies for 20 min at 4$^{o}$C in the dark. FACS were performed on a BD FACS Canto II and analyzed by FlowJo_V10. Live cells were separated from debris in an SSC-A (y) versus FSC-A (x) dot plot, doublets excluded with FSC-H (y) versus FSC-A (x) / SSC-H (y) versus SSC-A (x) dot plots. Singlets were analyzed and gated as indicated.

**Caspase-1 activation assay**

Caspase-1 activation assay was performed according to the manufacturer's protocol (FAM-FLICA® Caspase-1 Assay Kit, ImmunoChemistry, Cat#97). Adherent cells were trypsinized and washed twice in wash buffer, resuspended and incubated with FLICA at the dilution of 1:30 at 37oC for 1 hr., washed and analyzed by BD FACS Canto II at the channel of FTIC. Debris and doublets were excluded out from analysis.

**ELISA**

Cell culture media or total cell lysates were analyzed using the DuoSet® ELISA Development Systems (R&D, Cat#DY007). Plates were coated with a primary antibody (R&D, Cat#DY814-05) overnight one day ahead of the assay. Samples and standards were added to primary antibody

coated plates in duplicate, incubated for 2 hrs. at room temperature each with biotinylated antibody and HRP-conjugated streptavidin, followed by 20 min incubation with HRP color reagent, with 3 washes between each step. After the stop solution was added, optical density at 450nm-570nm was measure. Sample quantification was calculated according to the standard curve.

**LDH release assay**

Cell culture media were incubated for 30 min at room temperature with equal volume of the CytoTox 96 Non-Radioactive Cytotoxicity Assay reagent according to the manufacturer's protocol (Promega, Cat#G1780). Absorbance at 490nm wavelength was measured using a Molecular Device SpectraMax i3x microphate reader. The data was presented as LDH release (%) = [(unknown-negative) / (positive-negative)]x100%.

**Co-culture experiment**

Two sets of $2x10^4$ KR158-luc cells stably expressing a scrambled shRNA or shRNA against STING or AIM2 or both were seeded in each 60mm ceramic dish, then left untreated or treated with TTFields at 200 kHz for 3 days. Supernatants were then collected, filtered using 0.45µm filters, and then added to a 12-well plate with each well containing RPMI with 10% FBS and $10^6$ splenocytes freshly harvested from 6-8 weeks old male C57BL/6J mice, and cocultured for 3 days. On days 4 and 5, culture media from the remaining ceramic dishes were collected to replenish the co-culture. On day 6, co-cultured splenocytes ($CD45^+$) were immunophenotyped by flow cytometry.

**Intracranial immunization protocol**

All animal experiments were performed according to regulations and rule of institutional IACUC. KR158-luc and GL261-luc cells stably expressing a scrambled shRNA or shRNA against STING, AIM2 or both were untreated or treated with TTFields at 200 kHz for 3 days. $3x10^5$ and $0.5 x10^5$ TTFields-treated KR158-luc and GL261-luc cells, respectively, suspended in 3µl PBS were implanted slowly (1µl/min) in the posterior frontal lobe of the brain with 6-week-old male (for the KR158-luc model) and an equal number of male and female (for GL261-luc model) syngeneic C57BL/6J mice (Jackson Laboratory), at 2 mm lateral to the right and 3.5 mm deep with bregma as the reference point using an automated mouse stereotaxic apparatus (Stoelting's). Orthotopic tumor growth was monitored by bioluminescence imaging (see below). One cohort was euthanized at 2 weeks after implantation for immunophenotyping, while the rest were allowed to proceed to the survival endpoint. For immunophenotyping, blood, cervical lymph nodes, spleen, bone marrow were collected and digested to single cell suspension, filtered through 40µm filters and subjected to red blood cell lysis using a lysis buffer (BD, Cat#555899), if necessary. Mouse brains were embedded in OCT and stored at -80$^{o}$C until analysis. For the re-challenge experiment, $6x10^5$ and $1x10^5$ parental KR158-luc and GL261-luc cells in 5µl PBS, respectively, were injected intracranially into surviving mice at day 100 post initial injection and age- and sex-matched naïve mice. At 1 and 2 weeks and 2 and 3 weeks after re-challenge with parent KR158-luc and GL261-luc cells, respectively, PBMCs were collected through tail-vein phlebotomy for immunophenotyping. At 20 weeks post re-challenge in the KR158 model, surviving mice were euthanized and dcLNs, blood and spleens were collected for immunophenotyping. For control, a cohort of age- and sex-matched naïve mice were implanted orthotopically with $6x10^5$ parental

KR158-luc cells in 5µl PBS and the same tissues collected 2 weeks later for the same immunophenotyping analysis.

## In Vivo Imaging System (IVIS) Spectrum

To monitor brain tumor growth, animals were imaged using the IVIS system (Xenogen). Mice were anesthetized by isoflurane (5% induction and 2% maintenance). RediJect D-Luciferin Bioluminescent Substrate (PerkinElmer, Cat#UL08RV01) were injected into mice subcutaneously and images taken repeatedly until the bioluminescence signal reached its peak. The data was analyzed using Living Image software (Caliper Life Sciences).

## Single Cell RNA-seq Analysis of PBMCs

### *Sample processing*

All human experiments were performed according to regulations and rule of IRB. Cryopreserved PBMCs from patients were washed with PBS and viability verified by Trypan Blue staining (**Supplementary Table S2**). Single cell suspensions were loaded onto Chromium Single Cell Chip (10x Genomics) according to the manufacturer's instructions at a target capture rate of approximately 10,000 cells/sample. The pooled single-cell RNA-seq libraries were prepared using the Chromium Single Cell 3′ Solution (10x Genomics) according to the manufacturer's instructions. All paired samples of pre-TTFields (pre-TTF) and post-TTFields (post-TTF) treatment for each patient and the resulting libraries were processed in parallel in the same batch. In total, there were 3 batches. All single cell libraries were sequenced with an 8-base i7 sample

index read, including a 28-base read 1 containing cell barcodes and unique molecular identifiers (UMI) and a 150-base read 2 for mRNA insert on Illumina Novaseq. Sample characteristics are summarized in **Supplementary Table S3**.

***Data processing***

The main operations were performed using the Seurat R package (3.2.2) (6, 7), unless otherwise stated. When option parameters for function deviated from the default values, we provided details of the changes accordingly. Most of the changes to the default options were made to accommodate and leverage the large size of the dataset.

*Cell Ranger Aggregation:* Conversion of the raw sequencing data from the bcl to fastq format and the subsequent alignment to the reference genome GRCh38 (GENCODE v.24) and gene count were performed using the *cellranger* software (10x Genomics, version 4.0.0) with the command *cellranger mkfastq, the STAR aligner, and the command cellranger count,* respectively. Results from all libraries and batches were pooled together using the command *cellranger aggr* without normalization for dead cells as it will be handled downstream. The filtered background feature barcode matrix obtained from this step was used as input for sequential analysis.

*Normalization of UMI*: Using the global scaling normalization method, the feature expression for each cell was divided by the total expression, multiplied by the scale factor (10,000), and log transformed using the *Seurat R* function *NormalizeData* with method "Log Normalize".

*Seurat aggregation and correction for batch effect*: As the counts were from three different batches, to align cells and eliminate batch effects for dimension reduction and clustering, we adopted the multi dataset integration strategy as previously described (7). Briefly, "anchors cells" were identified between pairs of datasets and used to normalize multiple datasets from different

batches. Given the size of our datasets (a total of 193,760 cells), we chose a reference-based, reciprocal PCA variant of the method detailed in the Seurat R package (6, 7). First, we split the previously integrated dataset by batches, using the Seurat function *SplitObject.* Next, for each split object, we performed variable feature selection using the function *FindVariableFeatures.* Features for integration were selected using the function *SelectIntegrationFeatures* and PCA performed for each split object on the selected features. The anchor cells were identified by using the function *FindIntegrationAnchors* with the reference chosen as the largest among 3 batches and the reduction option set to 'rpca'. Finally, the whole datasets from 3 batches were reintegrated using the function *IntegrateData* with the identified anchor cells.

*UMAP dimension reduction*: The integrated multiple batch dataset was used as input for UMAP dimension reduction (8). The feature expression was scaled using the Seurat function *ScaleData*, followed by a PCA run using the function *RunPCA* (Seurat) with the total number of principal components (PC) to compute and store option of 100. The UMAP coordinates for single cells were obtained using the *RunUMAP* function (Seurat) with the top 75 PCs as input features (dims = 1:75) with min.dist = 0.75 and the number of training epochs n.epochs = 2000.

*Clustering of cells*: We relied on a graph-based clustering approach implemented in the Seurat package, which embeds cells in a K-nearest neighbor graph with edges drawn between similar cells and partitions nodes in the network into communities. Briefly, a Shared Nearest Neighbor graph was constructed using the *FindNeigbhors* function with an option dimension of reduction input dims = 1:75, error bound nn.eps = 0.5. This function calculates the neighborhood overlap (Jaccard index) between every cell and its k.param nearest neighbors(9). The graph was partitioned into clusters using the *FindClusters* function with different values for resolution parameter. The higher the resolution, the smaller the cluster size. We experimented with resolutions values of 0.1,

1, 3, 5, 10. Resolution 0.3 gave large clusters of all major cell types such as B and T cells without cell subtypes. Resolutions 3, 5 and 10 gave excessively small clusters, which are mostly patient specific making cross-patients generalization difficult. At the end we chose Resolutions 1 to perform downstream analyses as it produced reasonable cluster sizes, partitioning cells into 38 biologically recognized cell subtypes. The interactive 3D UMAP plots were generated for Resolution 1 by using R package plotly v 4.9.3 on the UMAP coordinates generated by Seurat as described previously (10). The differential expressed gene markers for each cluster were found using the *FindAllMarkers* function with the option of only returning positive markers and a minimal fraction of cells with the marker of 0.25. The default Wilcoxon Rank Sum test was used to calculate statistical differences in each cell cluster.

*Analytical plan in dead cell exclusion*: In all above analyses, we did not filter out dead cells before clustering, rather we used cluster-based dead cell exclusion. We experimented with filtering out dead cells before clustering by mitochondrial genes content, the average number of read UMI or average number of UMI per gene and did not find any reasonable threshold for our particular patient dataset. Even using a relaxed threshold for mitochondrial content <15% eliminated more than 40% of cells in some patients, even though when we stained cells using the Trypan Blue method to estimate the dead cell fraction, the dead cell fraction was never more than 10% (**Table S2**). We speculated that the abnormal elevated content of mitochondrial genes in our dataset is due to the significant stresses that these patients were under, including cancer diagnosis, recent radiotherapy and chemotherapy, and TTFields treatment, and steroid treatment, etc. As a result, we opted to analyze all cells without prefiltration for dead cells before clustering. Instead, dead cells were identified after clustering and dead cells formed a smear cluster (Clusters 16, 24, 28, and 30 in Resolution 1 UMAP) in the center of the UMAP map without clear cell-specific identity

and with elevated mitochondrial genes and housekeeping genes. Cells in these clusters were excluded from further analysis.

***TTFields treatment analysis***

*Cluster proportion changes*. The cluster proportion change after TTFields treatment for each cluster was performed using Wilcoxon signed rank test on paired values of log proportion of each patient pre TTFields and post TTFields treatment using wilcox.test with option pair=TRUE in R programming environment (version 4.0.3).

*Correlation between cluster proportion changes and diversity changes*. The log fold changes for each patient's cluster proportions and TCR diversity indices between pre TTFields and post TTFields treatment were calculated. Next, the Spearman correlation test was performed using the logFC of proportion changes and TCR diversity changes as input using the function cor.test, method="spearman", R programming environment (version 4.0.3).

*Gene differential expression analysis between pre TTFields and post TTFields paired samples.* The differential expression analysis was done using the LIMMA/Voom method (LIMMA R package) (11-13). Briefly, the single cell UMI counts matrix for each cluster was transformed to log2-counts per million (logCPM) with estimate for the mean-variance relationship and used to compute appropriate observation-level weights using the *voom* function. The transformed matrix was then fitted to linear model with timepoints and patients as factors using the function *lmfit.* A moderated t-statistics was computed using empirical Bayes moderation of the standard errors towards a global value using the function *eBayes*. Next, estimated coefficients and standard errors for the contrast of pre TTFields and post TTFields timepoints was calculated using the function

*contrasts.fit.* Contrast-specific, moderated t-statistics was then computed using the function *eBayes.* The logFC, t-statistics, p values were exported using the function *topTable.*

*Pathway differential expression analysis.* We used Gene Set Enrichment Analysis (GSEA) as previously described (14) for analysis of immune specific pathways of interest. For each cluster, all genes were ranked using the moderated t-test from the Gene differential expression analysis step above. Then GSEA (preranked, "classic" mode, 10,000 permutations) was performed to calculate enrichment for the pathways of interest, using the command lines and Java implementation of GSEA downloaded from http://software.broadinstitute.org/gsea/index.jsp.

*Heatmaps of logFC of gene expression and pathway activity for each cluster.* For each cluster, the gene counts for each library were calculated by summing up all the associated UMI counts of cells and normalized to transcript per million (tpm) unit by dividing the counts by the length of the genes in kilobases to obtain read per kilobase (RPK). RPK was normalized by dividing to the total RPK values of each library and expressed in millions, and log2 transformed. The log FC of gene expression of each patient between pre TTFields and post TTFields treatment was then calculated by subtracting the respective log tpm values.

For the global pathway activity logFC calculation, pathways and gene membership were downloaded from Gene Ontology http://geneontology.org/, selecting only those related to Biological Process. The activity for each pathway was calculated as an average tpm value of all genes in that pathway, and the log FC of a pathway of each patient between pre TTFields and post TTFields treatment calculated by dividing the pathway activity of post TTFields values by the pre TTFields values, followed by report and visualization by heatmaps.

*Pseudo-time calculation.* A diffusion map object was calculated first using the function *DiffusionMap*of R package *destiny* v 3.4.0 on PCA dimensions. The diffusion time was then derived by function DPT of *destiny* library with the diffusion map object from previous step as input. The pseudo-time for each cell was the rank of diffusion time for that cell.

*Heatmaps of T1IRG pathway scores at the single cell level.* The score was defined as the mean expression (normalized by Seurat function NormalizeData; in brief, feature counts for each cell are divided by the total counts for that cell and multiplied by 10000 and then natural-log transformed using log1p) of genes annotated as belonging to the Gene Ontology "response to type I interferon" GO:0034340. The gene set for GO:0034340 was downloaded from http://www.gsea-msigdb.org, and includes 99 genes: ABCE1, ADAR, BST2, CACTIN, CDC37, CNOT7, DCST1, EGR1, FADD, GBP2, HLA-A, HLA-B, HLA-C, HLA-E, HLA-F, HLA-G, HLA-H, HSP90AB1, IFI27, IFI35, IFI6, IFIT1, IFIT2, IFIT3, IFITM1, IFITM2, IFITM3, IFNA1, IFNA10, IFNA13, IFNA14, IFNA16, IFNA17, IFNA2, IFNA21, IFNA4, IFNA5, IFNA6, IFNA7, IFNA8, IFNAR1, IFNAR2, IFNB1, IKBKE, IP6K2, IRAK1, IRF1, IRF2, IRF3, IRF4, IRF5, IRF6, IRF7, IRF8, IRF9, ISG15, ISG20, JAK1, LSM14A, MAVS, METTL3, MIR21, MMP12, MUL1, MX1, MX2, MYD88, NLRC5, OAS1, OAS2, OAS3, OASL, PSMB8, PTPN1, PTPN11, PTPN2, PTPN6, RNASEL, RSAD2, SAMHD1, SETD2, SHFL, SHMT2, SP100, STAT1, STAT2, TBK1, TREX1, TRIM56, TRIM6, TTLL12, TYK2, UBE2K, USP18, WNT5A, XAF1, YTHDF2, YTHDF3, ZBP1. The gene set for GO:002437, a non-T1IRG inflammatory pathway used as a negative control, includes 73 genes: ADCYAP1, AHCY, AK7, BDKRB2, BTK, C3, CCR7, CD6, CD24A, CD28, CD68, CD81, CNR1, CXCR2, CYSLTR1, ELANE, EPHB6, EXT1, FCER1A, FCER1G, FCGR1, FCGR2B, FCGR3, FUT7, GPR17, GPX1, GPX2, H2-T23, HMGB2, ICAM1, IGH-7, IGH-8, IGHG1, IGHG2A, IGHG2B, IL1F10, IL1RN, IL2RA, IL5RA, IL10, IL12B, IL20RB,

IL25, IL31RA, IL36A, IL36B, IL36G, IL36RN, KARS, LILRB4A, LTA, MIR21A, MIR155, MIR301, NLRP6, NOD2, NOTCH1, NOTCH2, NPY5R, PARK7, PLA2G2D, PNMA1, PSMA1, PSMB4, RASGRP1, RBPJ, SELENOS, SERPINB9, SPN, TNF, TREX1, ZFP35, ZP3.

**Bulk RNA-seq of isolated T lymphocytes and TCR clonotyping**

***Sample preparation***

Untouched T cells were selected from PBMC single cell suspension using human pan T Cell isolation kit according to the manufacturer's instructions (Miltenyi Biotec, Cat#130-096-535). RNA was extracted utilizing QIAGEN RNeasy Midi Kit (Cat#75144) according to the manufacturer's instructions. Bulk RNAseq library was constructed, pooled and sequenced on a NovaSeq 6000 Illumina instrument at University of Florida Interdisciplinary Center for Biotechnology Research Gene Expression & Genotyping/NextGen Sequencing Core.

***Sequencing analysis***

Paired-end reads were trimmed with trimmomatic v/0.36. Alignment and gene counts were generated against the GRCh38.p12 genome assembly using the annotation GeneCode release 28 by STAR v2.6.0b with default options and quantmode=GeneCounts (**Table S2**). The heatmaps of logFC of gene expression and pathway activity were made similarly to the described procedure in sing cell analysis above.

***TCR Clonotyping***

To extract the T Cell receptor clones from bulk RNA-seq data, the pair end reads from bulk non-targeted RNA-seq were supplied to MiXCR v.3.0.13, an universal tool for analyzing T- and B-

cell receptor repertoire sequencing data (https://milaboratory.com/software/mixcr/) (15, 16), using the command *analyze shotgun* with the option of starting-material rna, only-productive. This command performed complicated pipeline, including alignment of raw sequencing reads, assembly of overlapping fragmented reads, inputting good TCR alignments, assembly of aligned sequences into clonotypes and exporting the resulting clonotypes into a tab-delimited file. For each sample, the Inverse Simpson Index was calculated using the vdjtools v 1.2.1 (https://github.com/mikessh/vdjtools) with the command *CalcDiversityStats* and input of clonotypes from the previous MixCR step. The clonal change plot was created using the Immunoarch R package v0.6.5 (https://cloud.r-project.org/web/packages/immunarch/index.html) with the function *trackClonotypes*, option col=”a.a”, to collapse all clones that share the same amino acid sequences.

**Field intensity and power density modeling**

Patient models were created as previously described (17). Briefly, contours of various regions in the tumor were created on an MRI sequence in a semiautomatic manner, and the full patient model created using a healthy head model that served as a deformable template. The primary ROI consisted of the enhancing tumor including the necrotic core or the resection cavity plus a 3mm boundary zone. The transducer array layouts assigned to the patients and the average electric current delivered to each patient (calculated from log files containing usage data downloaded directly from the memory bank of the TTFields device) were obtained from patient records. To simulate delivery of TTFields, virtual transducer arrays were automatically placed on the models according to the assigned layouts generated by NovoTAL. Standard electric properties were assigned to the various tissue types and materials in the model according to values previously

described (17). Boundary conditions were set so that the total current delivered to the patient was equal to the average current per channel delivered to the patient during the first month of treatment excluding readings of zero current. LMiFI (local minimum field intensity) and LAFI (local average field intensity), defined as the lower and average, respectively, of the 2 field intensities delivered to each point in the brain, and LMiPD (local minimum power density) within the models were calculated using the Sim4Life v6.2 Ohmic-electroquasistatic solver (ZMT Zurich, Zurich, Switzerland). LMiPD and LMiFI of the primary ROI were also calculated.

**Statistical Analyses**

GraphPad Prism 8 software was used for statistical analysis. For the cytosolic micronuclei cluster analysis with or without ribociclib, Fisher Exact Test was used to conduct an overall test comparing all 4 groups within each cell line and subsequent pairwise comparisons of interest with adjustment for multiple comparisons. We used corrections proposed by Holm (18), a less conservative approach. All Student's t-tests were two-sided and $P$ values $\leq 0.05$ (with 95% confidence interval) considered statistically significant for each of the specific statistical comparisons ($*P < 0.05$, $**P < 0.01$, $***P < 0.001$, $****P < 0.0001$). One-way ANOVA was used to adjust for multiple comparisons where appropriate, except for the TME gene set expression comparisons, for which two-way ANOVA was applied. Data with continuous outcomes are represented as mean $\pm$ S.E.M. For scRNA-seq, the comparison was based on annotated clusters comparing before and after TTFields treatment for each patient using the paired samples Wilcoxon test in R language. Spearman correlation coefficient was used to assess both linear and non-linear correlative relationships between pDC proportions and activity with TCR clonal expansion.

**References for the Methods section:**

1. Hoang-Minh LB, Siebzehnrubl FA, Yang C, Suzuki-Hatano S, Dajac K, Loche T, et al. Infiltrative and drug-resistant slow-cycling cells support metabolic heterogeneity in glioblastoma. Embo J. 2018;37(23):e98772.

2. Allen M, Bjerke M, Edlund H, Nelander S, Westermark B. Origin of the U87MG glioma cell line: Good news and bad news. Science Translational Medicine. 2016;8(354):354re3-re3.

3. Van Meir EG, Kikuchi T, Tada M, Li H, Diserens AC, Wojcik BE, et al. Analysis of the p53 gene and its expression in human glioblastoma cells. Cancer Res. 1994;54(3):649-52.

4. Ishii N, Maier D, Merlo A, Tada M, Sawamura Y, Diserens A-C, et al. Frequent Co-Alterations of TP53, p16/CDKN2A, p14ARF, PTEN Tumor Suppressor Genes in Human Glioma Cell Lines. Brain Pathol. 1999;9(3):469-79.

5. Chang E, Patel CB, Pohling C, Young C, Song J, Flores TA, et al. Tumor treating fields increases membrane permeability in glioblastoma cells. Cell Death Discovery. 2018;4(1):113.

6. Butler A, Hoffman P, Smibert P, Papalexi E, Satija R. Integrating single-cell transcriptomic data across different conditions, technologies, and species. Nat Biotechnol. 2018;36(5):411-20.

7. Stuart T, Butler A, Hoffman P, Hafemeister C, Papalexi E, Mauck WM, III, et al. Comprehensive Integration of Single-Cell Data. Cell. 2019;177(7):1888-902.e21.

8. Becht E, McInnes L, Healy J, Dutertre C-A, Kwok IWH, Ng LG, et al. Dimensionality reduction for visualizing single-cell data using UMAP. Nat Biotechnol. 2019;37(1):38-44.

9. Hegde S, Krisnawan VE, Herzog BH, Zuo C, Breden MA, Knolhoff BL, et al. Dendritic Cell Paucity Leads to Dysfunctional Immune Surveillance in Pancreatic Cancer. Cancer Cell. 2020;37(3):289-307.e9.

10. Carson Sievert CP, Toby Hocking, Scott Chamberlain, Karthik Ram, Marianne Corvellec, Pedro Despouy, Salim Brüggemann, and Plotly Technologies Inc. Interactive Web-Based Data Visualization with R, plotly, and shiny.: Chapman and Hall/CRC; 2020.

11. Phipson B, Lee S, Majewski IJ, Alexander WS, Smyth GK. Robust hyperparameter estimation protects against hypervariable genes and improves power to detect differential expression. Ann Appl Stat. 2016;10(2):946-63.

12. Ritchie ME, Phipson B, Wu D, Hu Y, Law CW, Shi W, et al. limma powers differential expression analyses for RNA-sequencing and microarray studies. Nucleic Acids Res. 2015;43(7):e47-e.

13. Genovese G, Carugo A, Tepper J, Robinson FS, Li L, Svelto M, et al. Synthetic vulnerabilities of mesenchymal subpopulations in pancreatic cancer. Nature. 2017;542(7641):362-6.

14. Subramanian A, Tamayo P, Mootha VK, Mukherjee S, Ebert BL, Gillette MA, et al. Gene set enrichment analysis: A knowledge-based approach for interpreting genome-wide expression profiles. Proceedings of the National Academy of Sciences. 2005;102(43):15545-50.

15. Bolotin DA, Poslavsky S, Mitrophanov I, Shugay M, Mamedov IZ, Putintseva EV, et al. MiXCR: software for comprehensive adaptive immunity profiling. Nature Methods. 2015;12(5):380-1.

16. Bolotin DA, Poslavsky S, Davydov AN, Frenkel FE, Fanchi L, Zolotareva OI, et al. Antigen receptor repertoire profiling from RNA-seq data. Nat Biotechnol. 2017;35(10):908-11.

17. Ballo MT, Urman N, Lavy-Shahaf G, Grewal J, Bomzon Z, Toms S. Correlation of tumor treating fields dosimetry to survival outcomes in newly diagnosed glioblastoma: a large-scale numerical simulation-based analysis of data from the phase 3 EF-14 randomized trial. Int J Radiat Oncol Biol Phys. 2019;104(5):1106–13.

18. Holm S. A Simple Sequentially Rejective Multiple Test Procedure. Scandinavian Journal of Statistics. 1979;6(2):65-70.

**Supplemental Table S1, supporting to FIGURE 11: Viability of cryopreserved PBMCs (% Live cell)**

| Patient | Pre TTF | Post TTF |
|---|---|---|
| P7 | 87 | 95 |
| P9 | 90 | 93 |
| P12 | 92 | 96 |
| P14 | 97 | 96 |
| P16 | 96 | 96 |
| P18 | 94 | 95 |
| P19 | 96 | 95 |
| P22 | 88 | 96 |
| P23 | 89 | 69 |
| P24 | 90 | 90 |
| P25 | 95 | 98 |
| P28 | 96 | 94 |

## Supplemental Table S2, supporting FIGURE 11: Parameters and quality control of single cell RNA-seq of PBMCs in 12 GBM patients

| Patient | P7 | | P9 | | P12 | | P14 | | P16 | | P18 | | P19 | | P22 | | P23 | | P24 | | P25 | | P28 | |
|---|---|---|---|---|---|---|---|---|---|---|---|---|---|---|---|---|---|---|---|---|---|---|---|---|
| TTF | Pre-TTF | Post-TTF | Pre-TTF | Post-TTF | Pre-TTF | Post-TTF | Pre-TTF | Post-TTF | Pre-TTF | Post-TTF | Pre-TTF | Post-TTF | Pre-TTF | Post-TTF | Pre-TTF | Post-TTF | Pre-TTF | Post-TTF | Pre-TTF | Post-TTF | Pre-TTF | Post-TTF | Pre-TTF | Post-TTF |
| Estimated Number of Cells | 8634 | 8213 | 5816 | 5003 | 8940 | 6395 | 8966 | 11414 | 12766 | 13031 | 6173 | 7058 | 4816 | 5046 | 5274 | 7171 | 6774 | 5542 | 8105 | 10869 | 11530 | 8003 | 11302 | 6919 |
| Mean Reads per Cell | 50849 | 47544 | 91050 | 87233 | 46880 | 66568 | 45847 | 34677 | 31671 | 30619 | 64666 | 51994 | 90634 | 84888 | 75065 | 47248 | 60568 | 83286 | 56752 | 40230 | 38888 | 53261 | 37767 | 58227 |
| Median Genes per Cell | 1573 | 1465 | 2042 | 2080 | 1069 | 1102 | 1154 | 1015 | 759 | 805 | 1138 | 1019 | 1015 | 1067 | 985 | 942 | 1208 | 1132 | 1506 | 888 | 1313 | 1389 | 1054 | 1018 |
| Number of Reads | 4.4E+08 | 3.9E+08 | 5.3E+08 | 4.4E+08 | 4.2E+08 | 4.3E+08 | 4.1E+08 | 4.0E+08 | 4.0E+08 | 4.0E+08 | 4.0E+08 | 3.7E+08 | 4.4E+08 | 4.3E+08 | 4.0E+08 | 3.4E+08 | 4.1E+08 | 4.6E+08 | 4.6E+08 | 4.4E+08 | 4.5E+08 | 4.3E+08 | 4.3E+08 | 4.0E+08 |
| Valid Barcodes | 96.60% | 96.90% | 97.20% | 97.20% | 98.20% | 98.30% | 98.10% | 98.20% | 98.20% | 98.20% | 98.30% | 98.20% | 98.30% | 98.40% | 98.30% | 98.30% | 98.10% | 98.10% | 98.10% | 97.90% | 97.50% | 97.90% | 97.90% | 97.60% |
| Sequencing Saturation | 68.50% | 69.70% | 79.30% | 78.50% | 82.60% | 87.40% | 79.00% | 73.90% | 78.30% | 79.00% | 87.30% | 85.70% | 92.30% | 90.70% | 89.90% | 86.60% | 81.80% | 86.80% | 76.80% | 80.90% | 72.00% | 76.80% | 76.80% | 84.50% |
| Q30 Bases in Barcode | 93.70% | 93.70% | 93.70% | 93.70% | 93.90% | 93.70% | 93.80% | 93.80% | 93.80% | 93.80% | 93.90% | 93.80% | 93.80% | 93.90% | 94.00% | 93.70% | 94.00% | 94.00% | 94.00% | 93.90% | 94.00% | 94.00% | 94.10% | 93.90% |
| Q30 Bases in RNA Read | 88.20% | 88.40% | 88.70% | 88.70% | 89.30% | 89.00% | 90.30% | 89.80% | 90.30% | 90.40% | 89.10% | 88.90% | 89.20% | 89.20% | 89.70% | 88.50% | 90.30% | 90.20% | 90.50% | 90.50% | 90.20% | 86.80% | 90.20% | 90.30% |
| Q30 Bases in UMI | 92.60% | 92.70% | 92.50% | 92.50% | 93.60% | 93.40% | 93.40% | 93.40% | 93.60% | 93.40% | 93.60% | 93.60% | 93.50% | 93.60% | 93.60% | 93.50% | 93.60% | 93.70% | 93.60% | 93.60% | 93.60% | 93.60% | 93.70% | 93.70% |
| Reads Mapped to Genome | 95.00% | 95.40% | 92.00% | 94.40% | 94.60% | 94.30% | 95.30% | 94.60% | 95.00% | 95.80% | 94.30% | 94.30% | 94.20% | 94.70% | 95.00% | 94.50% | 94.00% | 93.80% | 94.30% | 94.30% | 94.50% | 89.30% | 94.40% | 94.10% |
| Reads Mapped Confidently to Genome | 92.40% | 93.20% | 84.40% | 88.40% | 92.60% | 92.30% | 92.80% | 92.30% | 92.50% | 93.50% | 92.30% | 92.40% | 92.30% | 92.90% | 93.10% | 92.90% | 91.60% | 91.10% | 91.80% | 91.50% | 92.40% | 87.30% | 91.70% | 91.60% |
| Reads Mapped Confidently to Intergenic Regions | 5.50% | 5.20% | 6.70% | 5.80% | 4.80% | 4.90% | 5.20% | 5.70% | 5.20% | 5.40% | 4.80% | 4.30% | 4.70% | 5.50% | 4.90% | 4.50% | 5.90% | 7.10% | 5.10% | 5.60% | 5.20% | 5.20% | 6.00% | 6.60% |
| Reads Mapped Confidently to Intronic Regions | 33.20% | 31.40% | 30.10% | 34.50% | 36.30% | 35.10% | 39.90% | 38.30% | 41.20% | 39.60% | 33.00% | 32.70% | 36.20% | 31.90% | 35.40% | 30.90% | 42.00% | 41.90% | 41.40% | 42.70% | 44.00% | 43.80% | 44.70% | 43.80% |
| Reads Mapped Confidently to Exonic Regions | 53.60% | 56.50% | 47.70% | 48.20% | 51.60% | 52.30% | 47.70% | 48.30% | 46.20% | 48.40% | 54.50% | 55.40% | 51.30% | 55.50% | 52.80% | 57.40% | 43.70% | 42.10% | 45.30% | 43.20% | 43.20% | 38.20% | 41.00% | 41.20% |
| Reads Mapped Confidently to Transcriptome | 49.60% | 52.70% | 43.60% | 44.00% | 47.70% | 48.50% | 43.80% | 44.30% | 42.70% | 44.60% | 50.60% | 51.40% | 47.90% | 51.90% | 49.10% | 53.40% | 39.60% | 38.00% | 41.30% | 39.10% | 39.00% | 34.30% | 37.00% | 37.10% |
| Reads Mapped Antisense to Gene | 1.30% | 1.00% | 1.20% | 1.30% | 1.20% | 1.10% | 1.20% | 1.10% | 0.90% | 1.00% | 1.00% | 1.00% | 1.00% | 0.90% | 1.10% | 0.90% | 1.40% | 1.40% | 1.40% | 1.50% | 1.70% | 1.60% | 1.50% | 1.60% |
| Fraction Reads in Cells | 96.00% | 94.10% | 91.80% | 93.70% | 92.30% | 90.60% | 83.70% | 82.40% | 85.00% | 88.00% | 91.90% | 91.70% | 89.50% | 86.40% | 92.20% | 93.70% | 93.50% | 87.30% | 93.70% | 91.60% | 94.10% | 96.00% | 93.20% | 89.40% |
| Total Genes Detected | 21360 | 20887 | 20075 | 19816 | 20512 | 19805 | 20069 | 20453 | 19193 | 20270 | 19637 | 19631 | 18631 | 19193 | 19370 | 19661 | 20610 | 20326 | 21060 | 20421 | 21330 | 20780 | 21238 | 20291 |
| Median UMI Counts per Cell | 4979 | 4740 | 6510 | 6636 | 2717 | 2911 | 2675 | 2422 | 1837 | 1908 | 2916 | 2587 | 2301 | 2696 | 2574 | 2434 | 3462 | 3101 | 4065 | 1994 | 2971 | 3186 | 2435 | 2317 |

## Suplemental Table S3, supporting FIGURES 13-15: Parameters and quality control of bulk RNA-seq of enriched peripheral T lymphocytes in 12 patients with newly diagnosed GBM

| Patient | P7 | | P9 | | P12 | | P14 | | P16 | | P18 | | P19 | | P22 | | P23 | | P24 | | P25 | | P28 | |
|---|---|---|---|---|---|---|---|---|---|---|---|---|---|---|---|---|---|---|---|---|---|---|---|---|
| TTF | Pre-TTF | Post-TTF | Pre-TTF | Post-TTF | Pre-TTF | Post-TTF | Pre-TTF | Post-TTF | Pre-TTF | Post-TTF | Pre-TTF | Post-TTF | Pre-TTF | Post-TTF | Pre-TTF | Post-TTF | Pre-TTF | Post-TTF | Pre-TTF | Post-TTF | Pre-TTF | Post-TTF | Pre-TTF | Post-TTF |
| Mapping speed, Million of reads per hour | 8.60 | 4.76 | 4.71 | 8.56 | 8.42 | 8.28 | 7.94 | 7.35 | 8.41 | 6.19 | 7.58 | 3.69 | 8.74 | 5.16 | 8.19 | 7.78 | 2.76 | 5.21 | 4.85 | 7.95 | 6.02 | 8.30 | 3.10 | 8.50 |
| Number of input reads | 9.E+07 | 7.E+07 | 7.E+07 | 7.E+07 | 9.E+07 | 6.E+07 | 1.E+08 | 6.E+07 | 7.E+07 | 8.E+07 | 7.E+07 | 6.E+07 | 8.E+07 | 6.E+07 | 8.E+07 | 7.E+07 | 8.E+07 | 9.E+07 | 1.E+08 | 1.E+08 | 9.E+07 | 9.E+07 | 9.E+07 | 1.E+08 |
| Average input read length | 295 | 296 | 296 | 296 | 296 | 296 | 296 | 295 | 296 | 295 | 296 | 296 | 296 | 295 | 295 | 296 | 296 | 295 | 295 | 296 | 296 | 296 | 296 | 296 |
| **UNIQUE READS:** | | | | | | | | | | | | | | | | | | | | | | | | |
| Uniquely mapped reads number | 8.3E+07 | 7.2E+07 | 6.7E+07 | 6.7E+07 | 8.1E+07 | 5.4E+07 | 9.3E+07 | 5.5E+07 | 7.0E+07 | 6.9E+07 | 7.1E+07 | 5.6E+07 | 7.4E+07 | 5.9E+07 | 7.8E+07 | 7.0E+07 | 7.5E+07 | 9.0E+07 | 1.1E+08 | 9.5E+07 | 9.0E+07 | 9.1E+07 | 8.6E+07 | 9.3E+07 |
| Uniquely mapped reads % | 93.34% | 95.81% | 96.04% | 96.16% | 92.63% | 91.93% | 94.96% | 94.10% | 95.65% | 88.93% | 94.86% | 95.19% | 96.18% | 96.21% | 96.18% | 95.89% | 95.52% | 96.26% | 96.10% | 95.88% | 96.54% | 96.44% | 96.03% | 96.14% |
| Average mapped length | 291.75 | 292.43 | 292.18 | 292.82 | 291.40 | 291.60 | 290.84 | 287.15 | 291.92 | 276.17 | 289.64 | 290.68 | 291.96 | 291.16 | 292.09 | 292.29 | 290.34 | 292.74 | 291.82 | 292.80 | 293.44 | 293.85 | 292.62 | 292.90 |
| Number of splices: Total | 7.4E+07 | 6.7E+07 | 6.2E+07 | 6.7E+07 | 8.0E+07 | 5.7E+07 | 9.7E+07 | 5.1E+07 | 6.7E+07 | 6.0E+07 | 7.1E+07 | 4.7E+07 | 6.8E+07 | 5.1E+07 | 8.5E+07 | 8.4E+07 | 7.9E+07 | 8.5E+07 | 1.1E+08 | 1.0E+08 | 8.3E+07 | 9.3E+07 | 9.2E+07 | 9.4E+07 |
| Number of splices: Annotated (sjdb) | 7.3E+07 | 6.6E+07 | 6.1E+07 | 6.6E+07 | 7.9E+07 | 5.6E+07 | 9.5E+07 | 5.0E+07 | 6.6E+07 | 5.9E+07 | 7.0E+07 | 4.6E+07 | 6.7E+07 | 5.0E+07 | 8.4E+07 | 8.3E+07 | 7.8E+07 | 8.3E+07 | 1.1E+08 | 1.0E+08 | 8.2E+07 | 9.2E+07 | 9.1E+07 | 9.3E+07 |
| Number of splices: GT/AG | 7.3E+07 | 6.6E+07 | 6.1E+07 | 6.6E+07 | 8.0E+07 | 5.7E+07 | 9.6E+07 | 5.1E+07 | 6.7E+07 | 5.9E+07 | 7.0E+07 | 4.6E+07 | 6.7E+07 | 5.1E+07 | 8.4E+07 | 8.3E+07 | 7.9E+07 | 8.4E+07 | 1.1E+08 | 1.0E+08 | 8.2E+07 | 9.3E+07 | 9.1E+07 | 9.3E+07 |
| Number of splices: GC/AG | 5.0E+05 | 4.5E+05 | 4.2E+05 | 4.5E+05 | 4.9E+05 | 3.8E+05 | 6.7E+05 | 3.6E+05 | 4.6E+05 | 3.1E+05 | 4.7E+05 | 3.3E+05 | 4.7E+05 | 3.5E+05 | 5.5E+05 | 5.2E+05 | 5.0E+05 | 5.6E+05 | 7.4E+05 | 6.7E+05 | 5.6E+05 | 6.3E+05 | 5.9E+05 | 6.2E+05 |
| Number of splices: AT/AC | 5.7E+04 | 5.5E+04 | 5.3E+04 | 5.6E+04 | 6.2E+04 | 4.3E+04 | 7.3E+04 | 3.9E+04 | 5.0E+04 | 1.4E+04 | 5.8E+04 | 3.7E+04 | 6.0E+04 | 4.3E+04 | 7.5E+04 | 6.2E+04 | 5.7E+04 | 6.6E+04 | 9.8E+04 | 9.0E+04 | 7.1E+04 | 7.0E+04 | 8.1E+04 | 8.2E+04 |
| Number of splices: Non-canonical | 6.9E+04 | 5.8E+04 | 5.7E+04 | 6.3E+04 | 8.2E+04 | 5.8E+04 | 9.7E+04 | 5.5E+04 | 6.5E+04 | 5.7E+04 | 7.3E+04 | 5.0E+04 | 7.4E+04 | 5.5E+04 | 8.3E+04 | 7.6E+04 | 7.2E+04 | 7.9E+04 | 1.1E+05 | 1.0E+05 | 7.1E+04 | 8.3E+04 | 1.1E+05 | 1.0E+05 |
| Mismatch rate per base, % | 0.27% | 0.25% | 0.26% | 0.25% | 0.26% | 0.25% | 0.23% | 0.28% | 0.25% | 0.39% | 0.28% | 0.28% | 0.27% | 0.29% | 0.25% | 0.25% | 0.26% | 0.25% | 0.28% | 0.25% | 0.24% | 0.22% | 0.27% | 0.25% |
| Deletion rate per base | 0.01% | 0.01% | 0.01% | 0.01% | 0.01% | 0.01% | 0.01% | 0.01% | 0.01% | 0.04% | 0.01% | 0.01% | 0.01% | 0.01% | 0.01% | 0.01% | 0.01% | 0.01% | 0.01% | 0.01% | 0.01% | 0.01% | 0.01% | 0.01% |
| Deletion average length | 1.86 | 1.89 | 1.92 | 1.92 | 1.84 | 1.88 | 1.81 | 1.85 | 1.88 | 1.45 | 1.95 | 1.99 | 1.95 | 1.93 | 1.83 | 1.92 | 1.83 | 1.84 | 1.89 | 1.92 | 1.93 | 1.90 | 2.03 | 2.03 |
| Insertion rate per base | 0.01% | 0.01% | 0.01% | 0.01% | 0.01% | 0.01% | 0.01% | 0.01% | 0.01% | 0.01% | 0.01% | 0.01% | 0.01% | 0.01% | 0.01% | 0.01% | 0.01% | 0.01% | 0.01% | 0.01% | 0.01% | 0.01% | 0.01% | 0.01% |
| Insertion average length | 1.70 | 1.80 | 1.78 | 1.78 | 1.60 | 1.63 | 1.73 | 1.75 | 1.76 | 1.85 | 1.72 | 1.71 | 1.79 | 1.78 | 1.74 | 1.81 | 1.79 | 1.72 | 1.72 | 1.73 | 1.70 | 1.71 | 1.67 | 1.67 |
| **MULTI-MAPPING READS:** | | | | | | | | | | | | | | | | | | | | | | | | |
| Number of reads mapped to multiple loci | 5.9E+06 | 3.1E+06 | 2.7E+06 | 2.6E+06 | 6.5E+06 | 4.7E+06 | 4.9E+06 | 3.4E+06 | 3.1E+06 | 8.6E+06 | 3.8E+06 | 2.8E+06 | 2.9E+06 | 2.3E+06 | 3.1E+06 | 3.0E+06 | 3.5E+06 | 3.4E+06 | 4.2E+06 | 4.1E+06 | 3.2E+06 | 3.3E+06 | 3.5E+06 | 3.7E+06 |
| % of reads mapped to multiple loci | 6.60% | 4.13% | 3.92% | 3.80% | 7.34% | 8.03% | 4.99% | 5.85% | 4.31% | 11.01% | 5.09% | 4.75% | 3.77% | 3.74% | 3.79% | 4.08% | 4.43% | 3.69% | 3.87% | 4.08% | 3.42% | 3.52% | 3.93% | 3.82% |
| Number of reads mapped to too many loci | 15605 | 12488 | 10755 | 7850 | 12358 | 6867 | 14113 | 15464 | 10342 | 36498 | 16980 | 13686 | 11279 | 9085 | 7437 | 6652 | 15039 | 14596 | 11546 | 10260 | 12102 | 11215 | 8368 | 10525 |
| % of reads mapped to too many loci | 0.02% | 0.02% | 0.02% | 0.01% | 0.01% | 0.01% | 0.01% | 0.03% | 0.01% | 0.05% | 0.02% | 0.02% | 0.01% | 0.01% | 0.01% | 0.01% | 0.02% | 0.02% | 0.01% | 0.01% | 0.01% | 0.01% | 0.01% | 0.01% |
| **UNMAPPED READS:** | | | | | | | | | | | | | | | | | | | | | | | | |
| % of reads unmapped: too many mismatches | 0.00% | 0.00% | 0.00% | 0.00% | 0.00% | 0.00% | 0.00% | 0.00% | 0.00% | 0.00% | 0.00% | 0.00% | 0.00% | 0.00% | 0.00% | 0.00% | 0.00% | 0.00% | 0.00% | 0.00% | 0.00% | 0.00% | 0.00% | 0.00% |
| % of reads unmapped: too short | 0.00% | 0.00% | 0.00% | 0.00% | 0.00% | 0.00% | 0.00% | 0.00% | 0.00% | 0.00% | 0.00% | 0.00% | 0.00% | 0.00% | 0.00% | 0.00% | 0.00% | 0.00% | 0.00% | 0.00% | 0.00% | 0.00% | 0.00% | 0.00% |
| % of reads unmapped: other | 0.04% | 0.04% | 0.03% | 0.02% | 0.02% | 0.03% | 0.04% | 0.02% | 0.03% | 0.02% | 0.03% | 0.04% | 0.03% | 0.03% | 0.02% | 0.03% | 0.04% | 0.04% | 0.02% | 0.02% | 0.03% | 0.03% | 0.03% | 0.03% |
| **CHIMERIC READS:** | | | | | | | | | | | | | | | | | | | | | | | | |
| Number of chimeric reads | 1.8E+06 | 1.6E+06 | 1.4E+06 | 1.3E+06 | 1.5E+06 | 1.5E+06 | 1.7E+06 | 1.5E+06 | 1.5E+06 | 4.9E+05 | 1.5E+06 | 1.0E+06 | 1.6E+06 | 1.4E+06 | 1.9E+06 | 1.3E+06 | 1.3E+06 | 1.6E+06 | 2.4E+06 | 1.8E+06 | 1.3E+06 | 1.2E+06 | 1.8E+06 | 2.3E+06 |
| % of chimeric reads | 1.99% | 2.11% | 2.02% | 1.93% | 1.68% | 2.53% | 1.69% | 2.51% | 2.00% | 0.63% | 1.98% | 1.75% | 2.11% | 2.31% | 2.32% | 1.74% | 1.63% | 1.74% | 2.21% | 1.85% | 1.35% | 1.27% | 1.99% | 2.43% |

**Supplemental Table S4, supporting FIGURE 11: Summary of TTFields array layouts, current intensities, LMiPD and LAFI**

| Patient | NovoTAL Layout | Post-TTF AP current [mA peak-peak] | Post-TTF LR current [mA peak-peak] | Pre-TTF LMiPD [mW/cm$^3$] | Pre-TTF LAFI [V/cm] | Post-TTF LMiPD [mW/cm$^3$] | Post-TTF LAFI [V/cm] |
|---|---|---|---|---|---|---|---|
| 7 | A | 1140.02 | 1439.919 | 1.80 | 1.33 | 0.61 | 0.88 |
| 9 | A | 947.3333 | 1188.929 | 1.91 | 1.35 | 0.51 | 0.72 |
| 14 | A | 1089.777 | 1225.921 | 3.17 | 1.68 | 1.07 | 0.97 |
| 16 | A | 994.575 | 1106.182 | 1.62 | 1.84 | 0.48 | 0.95 |
| 18 | A | 1192.873 | 1209.197 | 1.20 | 0.97 | 0.44 | 0.58 |
| 19 | A | 1236.936 | 1448.42 | 2.73 | 1.55 | 1.17 | 1.04 |
| 23 | C | 1258.897 | 1516.484 | 2.59 | 1.55 | 1.06 | 1.10 |
| 24 | A | 1025.661 | 1442.975 | 2.73 | 1.62 | 0.80 | 1.01 |
| 25 | A | 1093.704 | 1332.822 | 1.26 | 1.42 | 0.38 | 0.89 |

Values represent field and current delivery to the gross tumor volume (GTV) including the enhancing tumor plus the necrotic core or resection cavity and a 3mm peritumor boundary zone margin (PBZ3)

**Supplemental Table S5, supporting FIGURE 12H-K: Top T1IRG and T2IRG pathways upregulated after TTFields in pDCs (C31)**

| PATHWAY NAME PER GENE ONTOLOGY (GO) | SIZE | NES | NOM p-val |
|---|---|---|---|
| GO_REGULATION_OF_RESPONSE_TO_INTERFERON_GAMMA | 21.00 | 1.86 | 0.02 |
| GO_CELLULAR_RESPONSE_TO_INTERFERON_ALPHA | 8.00 | 1.83 | 0.02 |
| GO_RESPONSE_TO_TYPE_I_INTERFERON | 73.00 | 1.81 | 0.01 |
| GO_RESPONSE_TO_INTERFERON_GAMMA | 139.00 | 1.75 | 0.02 |
| GO_INTERFERON_GAMMA_MEDIATED_SIGNALING_PATHWAY | 73.00 | 1.40 | 0.11 |
| GO_POSITIVE_REGULATION_OF_TYPE_I_INTERFERON_MEDIATED_SIGNALING_PATHWAY | 10.00 | 1.32 | 0.16 |
| GO_NEGATIVE_REGULATION_OF_RESPONSE_TO_INTERFERON_GAMMA | 5.00 | 1.28 | 0.18 |
| GO_RESPONSE_TO_INTERFERON_ALPHA | 17.00 | 1.28 | 0.18 |
| GO_REGULATION_OF_TYPE_I_INTERFERON_MEDIATED_SIGNALING_PATHWAY | 27.00 | 1.20 | 0.25 |
| GO_TYPE_I_INTERFERON_PRODUCTION | 103.00 | 1.00 | 0.42 |
| GO_NEGATIVE_REGULATION_OF_TYPE_I_INTERFERON_MEDIATED_SIGNALING_PATHWAY | 10.00 | 0.94 | 0.51 |

**Supplemental Table S6, supporting FIGURE 13A and Supplemental Figure S23A:** Simpson diversity index of TCRA/B V(D)J in peripheral T cells

| | TCRA Simpson Diversity Index | | | TCRB Simpson Diversity Index | | |
|---|---|---|---|---|---|---|
| **Patient** | **Pre TTF** | **Post TTF** | **logFC** | **Pre TTF** | **Post TTF** | **logFC** |
| **P7** | 371.6 | 323.7 | -0.2 | 774.0 | 645.0 | -0.26 |
| **P9** | 316.3 | 341.8 | 0.11 | 398.0 | 468.0 | 0.51 |
| **P12** | 19.7 | 179.5 | 3.19 | 23.0 | 108.0 | 2.22 |
| **P14** | 86.9 | 36.4 | -1.25 | 155.0 | 107.0 | -0.54 |
| **P16** | 268.6 | 21.4 | -3.65 | 443.0 | 59.0 | -2.92 |
| **P18** | 255.7 | 88.6 | -1.53 | 381.0 | 138.0 | -1.47 |
| **P19** | 37.0 | 14.5 | -1.35 | 95.0 | 20.0 | -2.24 |
| **P22** | 174.8 | 157.4 | -0.15 | 81.0 | 139.0 | 0.77 |
| **P23** | 15.8 | 8.9 | -0.83 | 16.0 | 7.0 | -1.27 |
| **P24** | 132.8 | 166.6 | 0.33 | 217.0 | 174.0 | -0.32 |
| **P25** | 380.4 | 279.4 | -0.45 | 535.0 | 504.0 | -0.09 |
| **P28** | 94.4 | 61.2 | -0.63 | 54.0 | 46.0 | -0.23 |

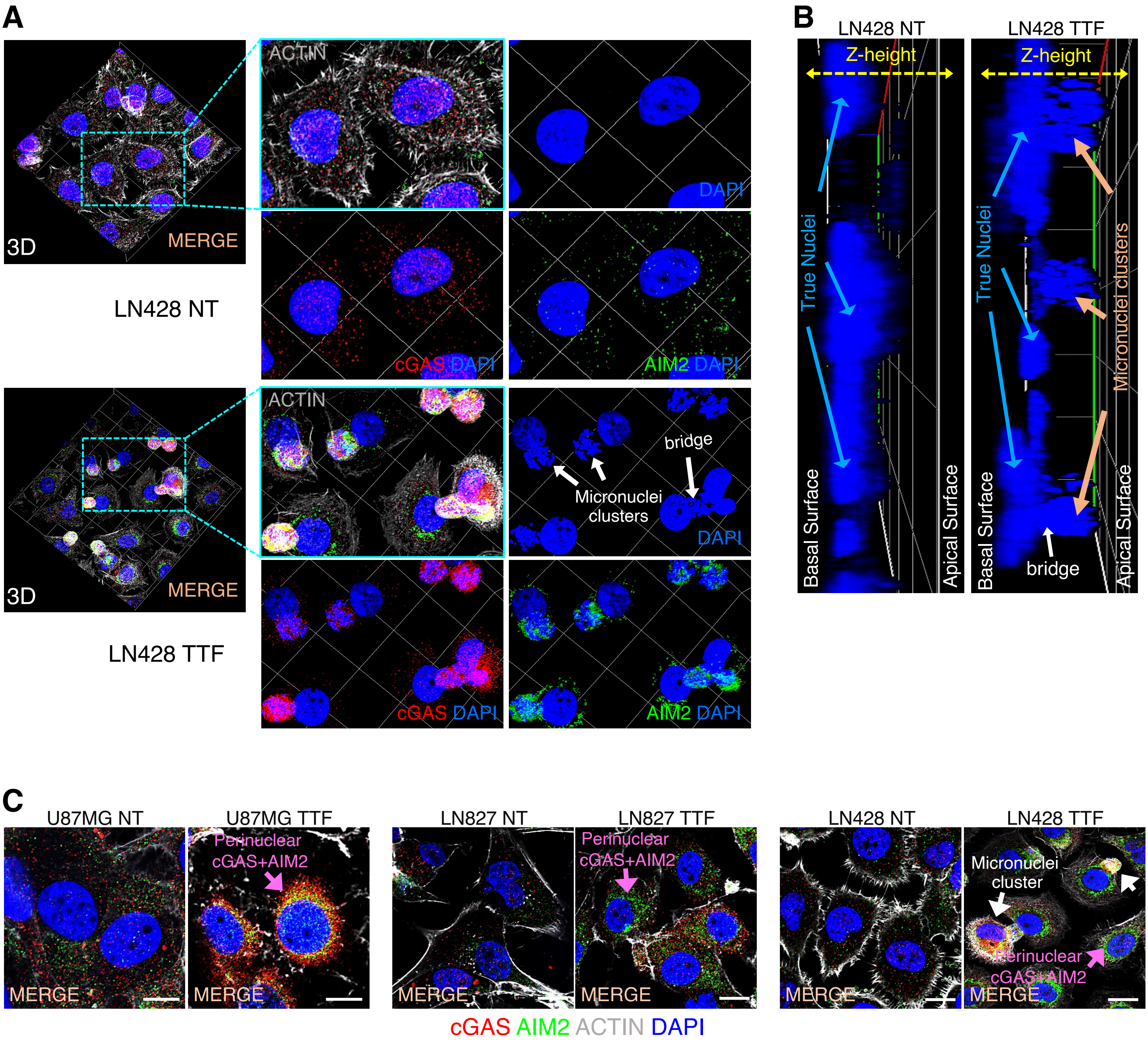


**Figure S1, supporting FIG. 1: TTFields-induced cytosolic micronuclei clusters recruit cGAS and AIM2 in GBM cell lines**.

**A)** 3D confocal images showing immunofluorescence staining for cGAS and AIM2 and counter-stained with Bactin for cytoplasmic outline and DAPI for DNA in LN428 GBM cells either non-treated (NT) (top) or treated with TTFields (TTF) (bottom) for 24 hrs. Each square is 30μm$^2$; Z-height is 15μm**.**

**B)** Side view of the Z axis of DAPI counterstain of LN428 GBM cells before and after TTFields showing cytosolic micronuclei clusters protrude directly from the true nuclei through a narrow bridge. Z-height is 15μm.

**C)** Confocal images of immunofluorescence staining (IF) of cGAS and AIM2 with Bactin for cytoplasmic outline and DAPI for nuclear counter-staining in the 3 GBM cell lines either non-treated (NT) or treated with TTFields (TTF) for 24 hrs. showing perinuclear redistribution of cGAS and AIM2 in TTFields-treated cells independent of micronuclei clusters. Scale bar: 10μm.

All data are representatives of at least 3 independent experiments.

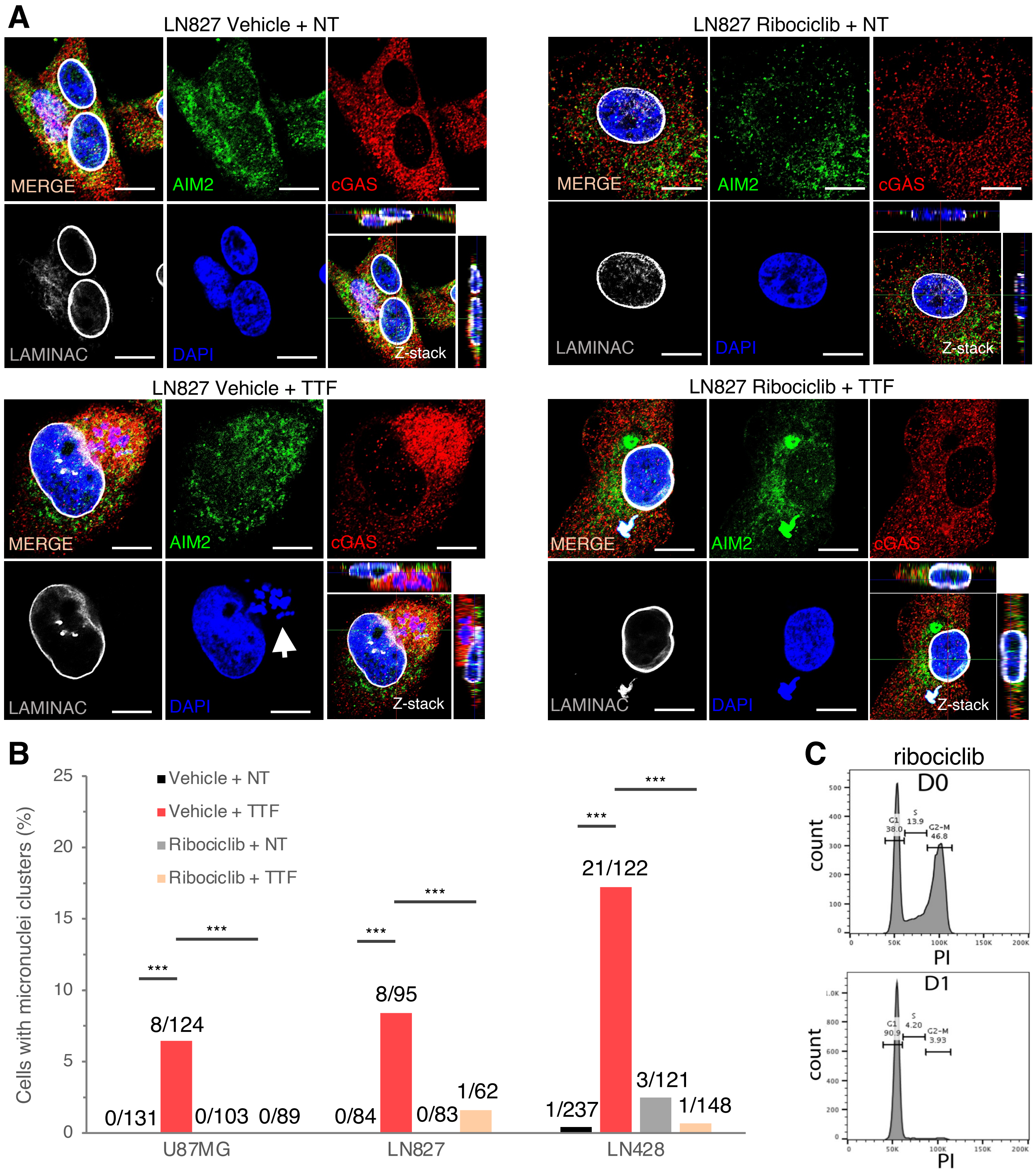


**Figure S2, supporting FIG. 1D: Cell cycle entry is required for TTFields-induced cytosolic micronuclei cluster formation in human GBM cells.**

**A**) Confocal images with Z stack showing IF for cGAS, AIM2, and LAMINAC with DAPI counter-stain in LN827 GBM cells pretreated with either vehicle or ribociclib (4.5 μM) to induce $G_1$ arrest, followed by treatment with TTFields for 24 hrs., demonstrating that S phase entry is required for TTFields-induced cytosolic micronuclei clusters. Scale bar: 10μm.

**B**) A bar plot showing quantification as percentages of the experiments in Figures **S5A** above and **S6**. The ratio above each bar represents the number of cells with clusters over the total cells counted. Fisher Exact Test was used to conduct an overall test comparing all 4 groups within each cell line and subsequent pairwise comparisons of interest with adjustment for multiple comparisons. ***, $P$ <0.001.

**C**) Representative histograms showing DNA content analysis by PI staining of LN827 cells treated with ribociclib (4.5μM) for 0 and 24 hrs. demonstrating effective $G_1$/S arrest.

All data are representatives of at least 3 independent experiments.

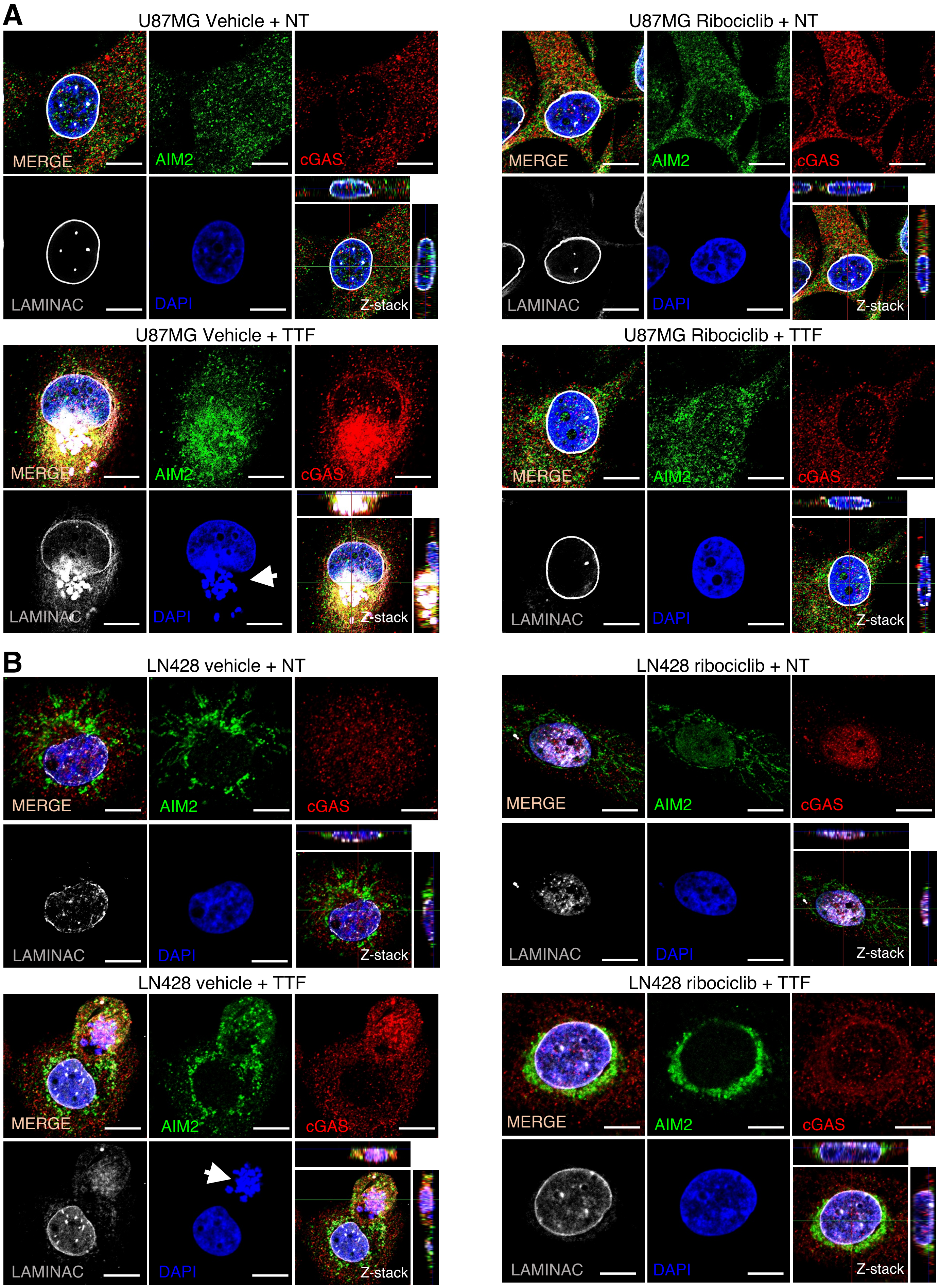


**Figure S3, supporting FIG. 1D and Fig. S2: Cell cycle entry is required for TTFields-induced cytosolic micronuclei cluster formation in U87MG and LN428 GBM cells.** Confocal images showing IF of cGAS, AIM2, and LAMINAC with DAPI counterstain in U87MG (**A**) and LN428 (**B**) cells pretreated with vehicle or ribociclib (4.5µM) to induce $G_1$ arrest, followed with or without TTFields for 24 hrs. demonstrate that S phase entry is required for TTFields-induced cytosolic micronuclei clusters. Scale bar: 10µm.

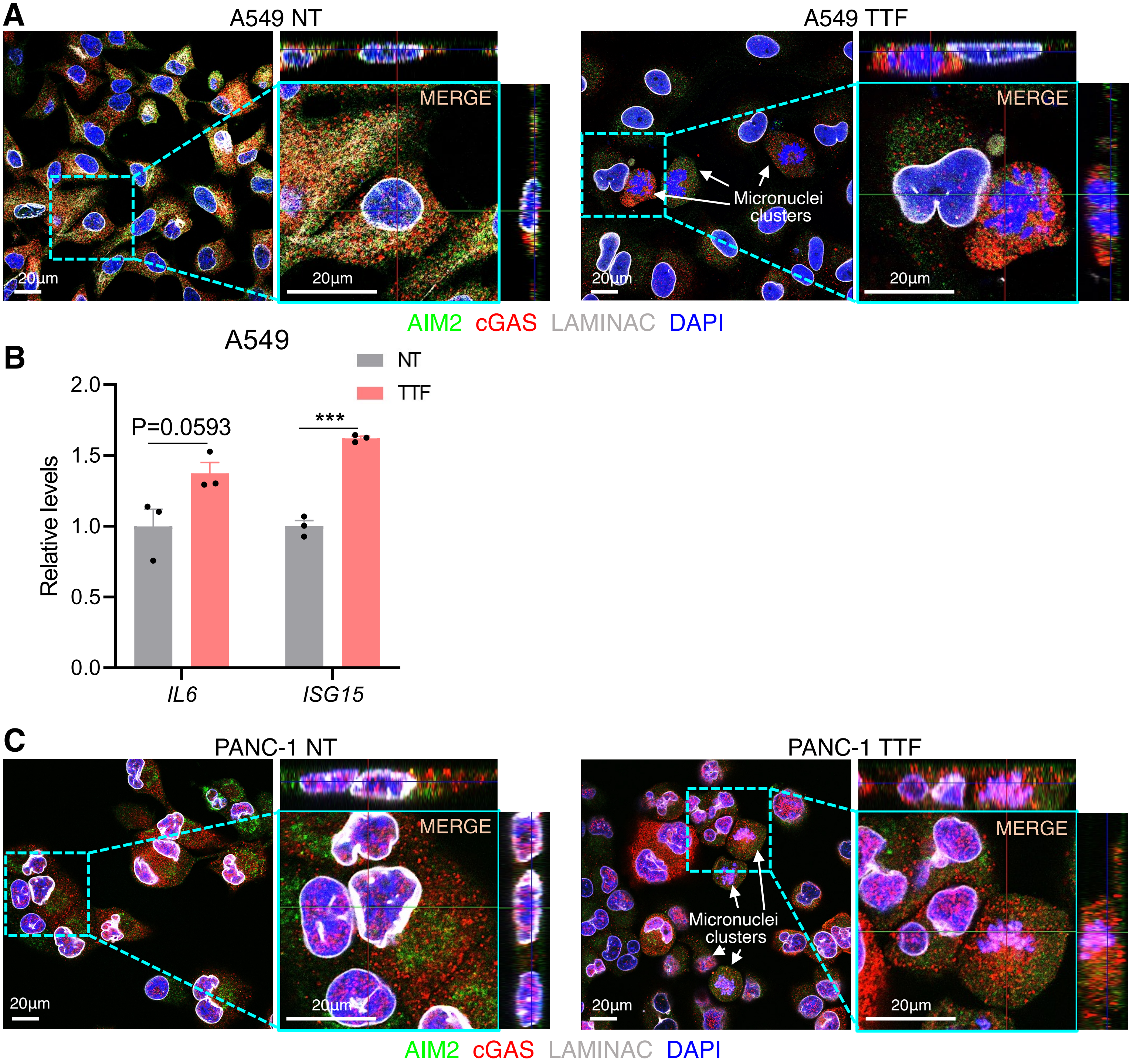


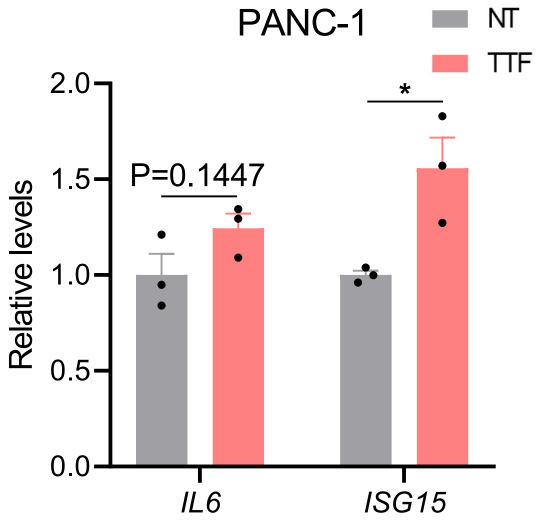


**Figure S4, supporting FIG. 1: TTFields similarly activate the cGAS/STING and AIM2/Caspase-1 inflammasomes in other solid tumor cells.**

Confocal images with Z stack showing that treatment with TTFields at 150 kHz for 24 hrs. resulted in large cytosolic micronuclei clusters that recruited both cGAS and AIM2 in the lung adenocarcinoma cell line A549 (**A**) and the pancreatic adenocarcinoma cell line PANC-1 (**C**), as determined by IF for cGAS, AIM2, and LAMINAC with DAPI counter-staining. Both the PIC *IL6* and the T1IRG *ISG15* were regulated in response to TTFields in these cell lines (**B** and **D**).

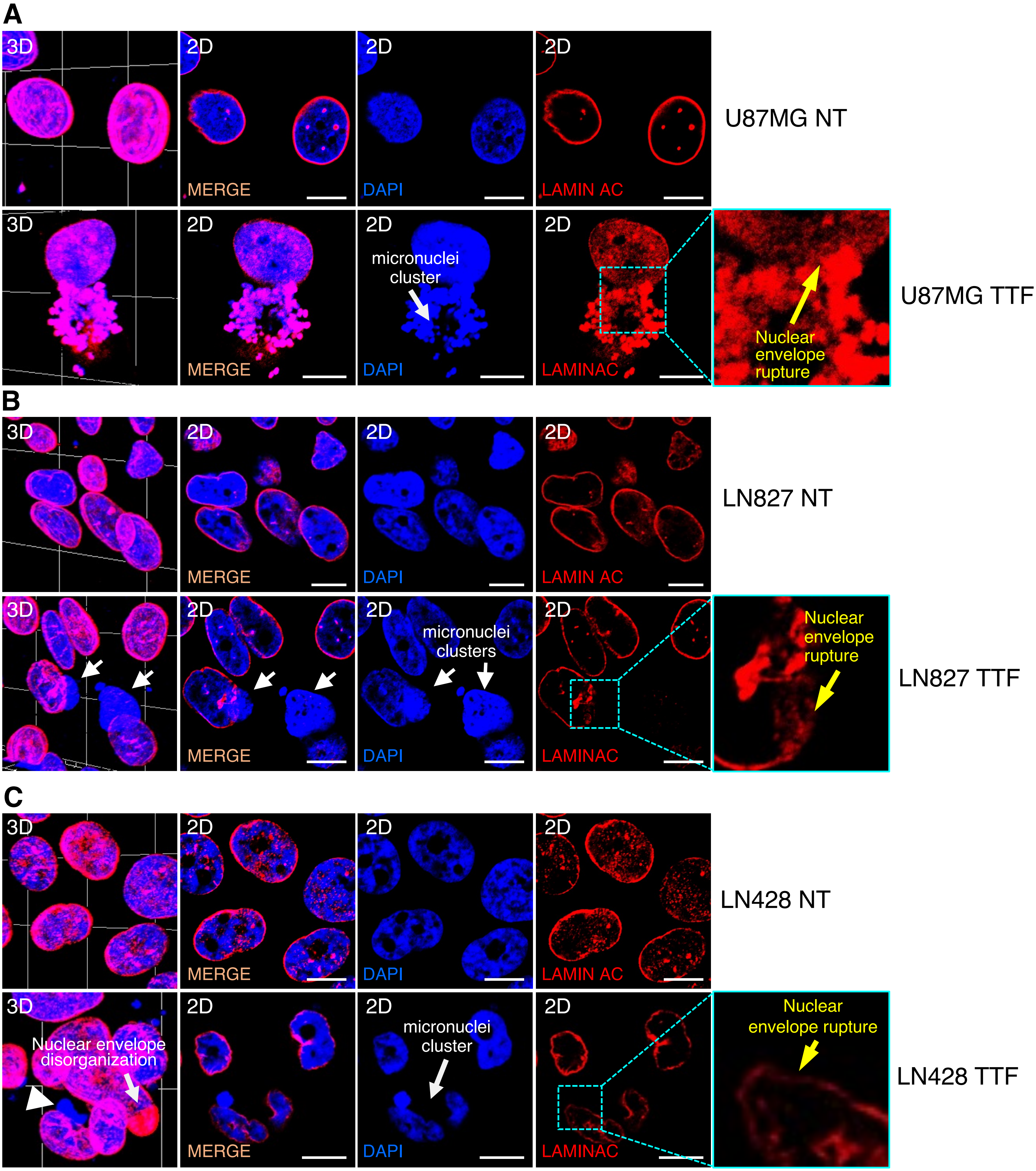


**Figure S5, supporting FIG. 1C: TTFields induce focal rupture in the nuclear envelope in GBM cell lines, through which nuclear content protrudes to form large cytosolic naked micronuclei clusters.**

Confocal images with 3D reconstruction showing IF of LAMINAC and DAPI for nuclear counter-staining in U87MG (**A**), LN827 (**B**) and LN428 (**C**) GBM cells either non-treated (NT) or treated with TTFields (TTF) for 24 hrs. Scale bar: 10µm. For the 3D images, each square is 30$\mu m^2$; Z-height is 15µm.

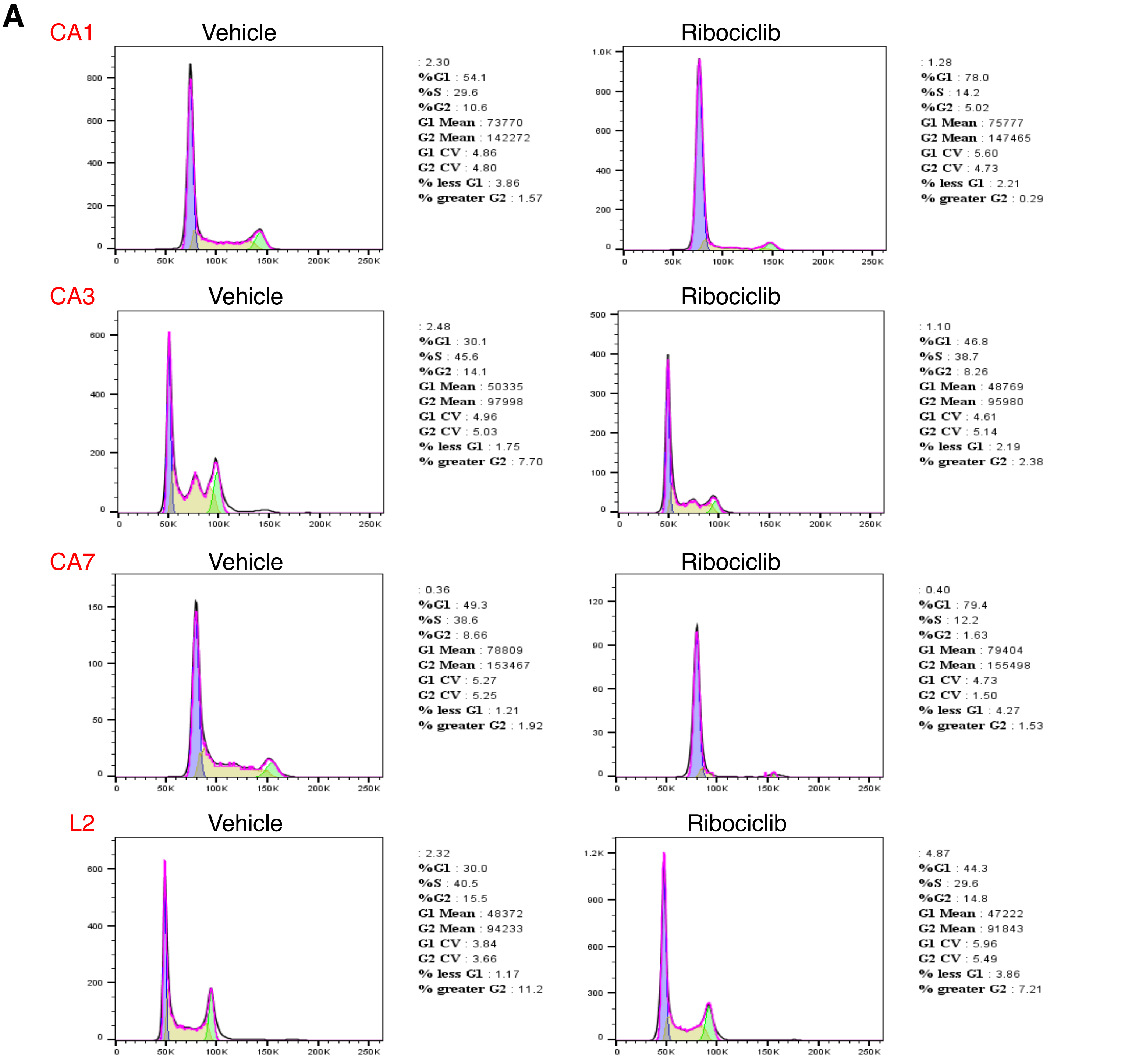


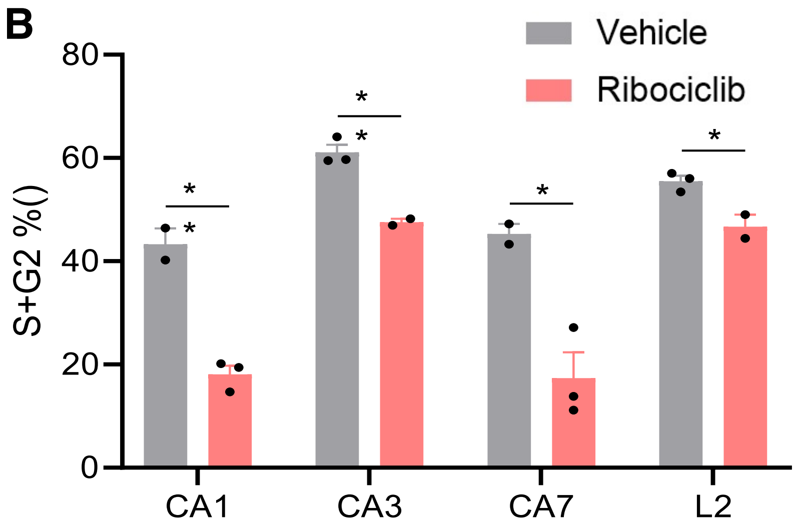


**Figure S6, supporting FIG. 1D: G1/S arrest in human GSCs using the CDK4/6 inhibitor ribociclib.**

(**A**) Representative histograms of 3 independent experiments showing DNA content analysis by propidium iodide (PI) staining of CA1, CA3, CA7 and L2 GSC lines treated with ribociclib (4.5μM) or the vehicle for 24 hrs, demonstrating effective $G_1$/S arrest in CA1 and CA7, moderate arrest in CA3, and minimal arrest in L2 cells as quantified in percentages of cells in S+G2 phases (**B**).

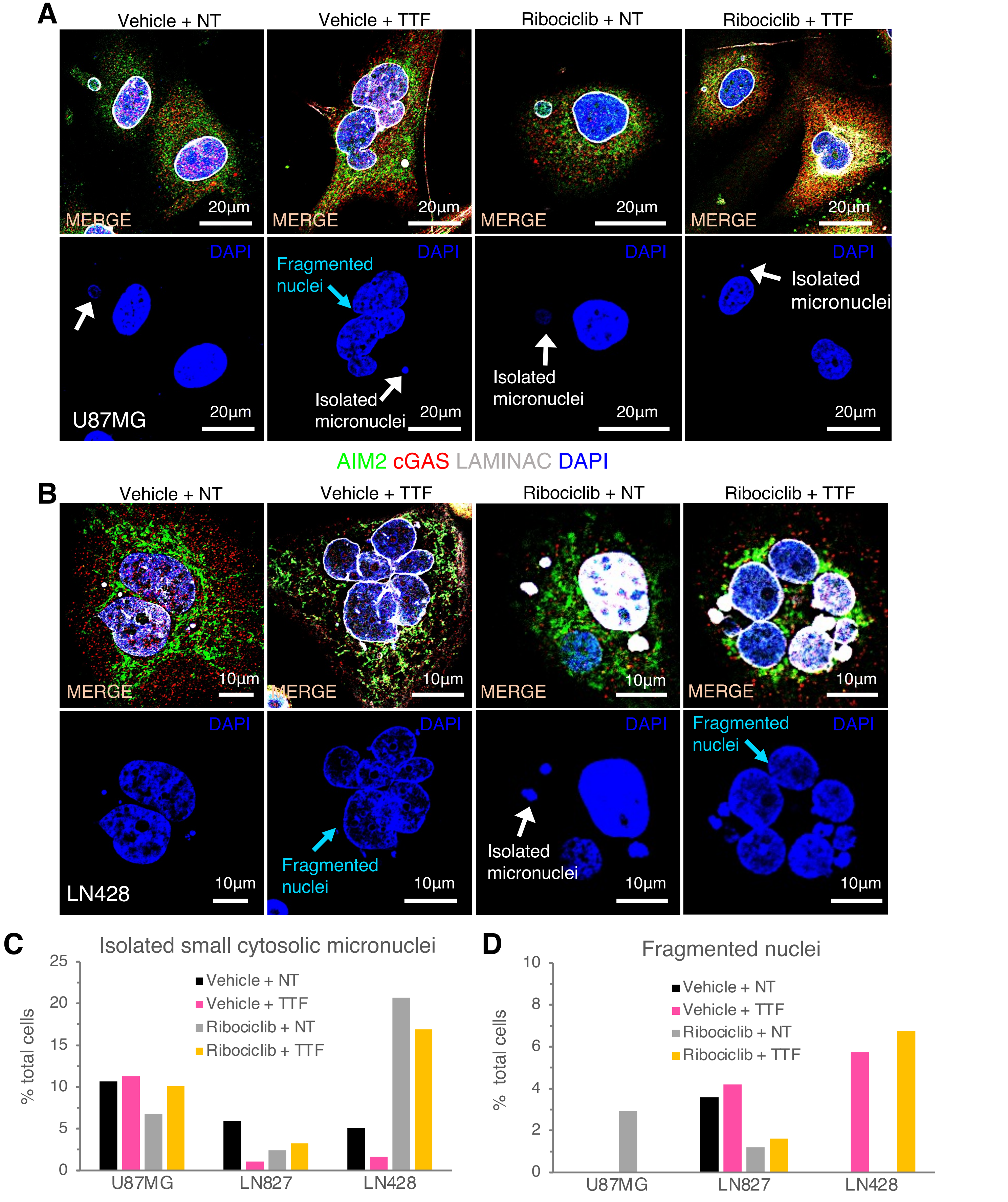


**Figure S7, supporting FIG. 1 and Figs. S2-3: Isolated cytosolic micronuclei and fragmented nuclei form independently of TTFields and cell cycle, protected by a nuclear membrane, and do not recruit cGAS and AIM2.** **A-B**) Confocal images showing IF for cGAS, AIM2, and LAMINAC with DAPI counterstain in U87MG (**A**) and LN428 (**B**) cells pretreated with the vehicle or ribociclib (4.5µM) to induce $G_1$ arrest, then treated with TTFields for 24 hrs. **C-D**) Bar plots showing percentages of cells with isolated free-standing cytosolic micronuclei (**C**) and fragmented nuclei (**D**) in the 3 GBM cell lines treated with various conditions in (**A-B**). These entities occurred independently of TTFields and cell cycle, were protected by a LAMINAC-based nuclear envelope and did not recruit cGAS and AIM2.

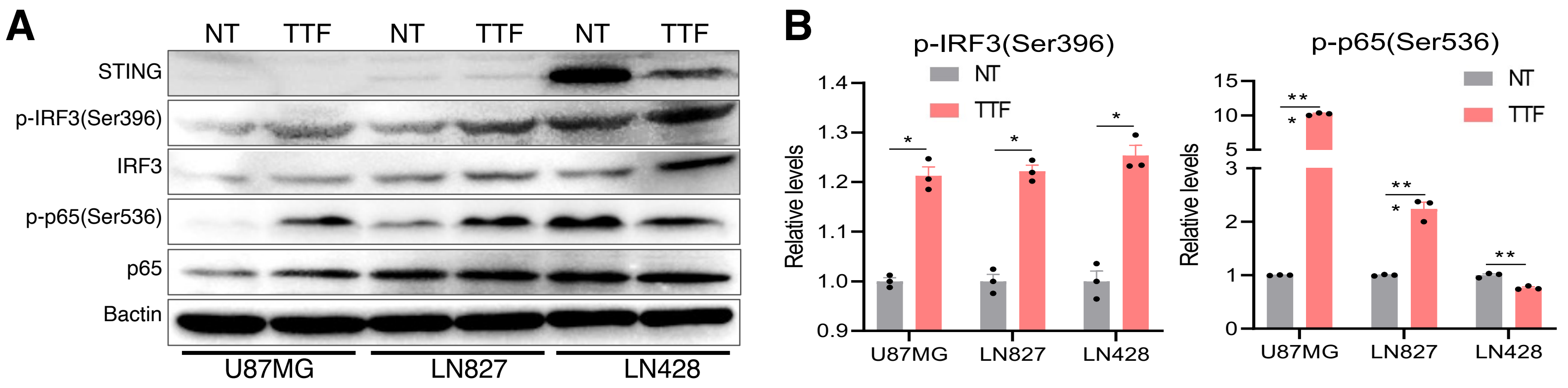


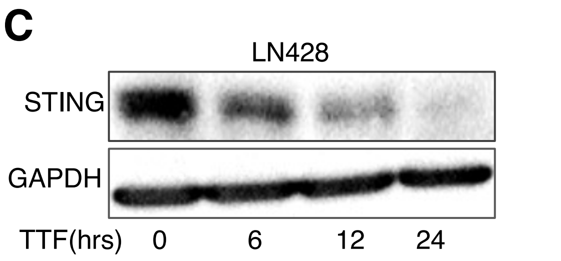


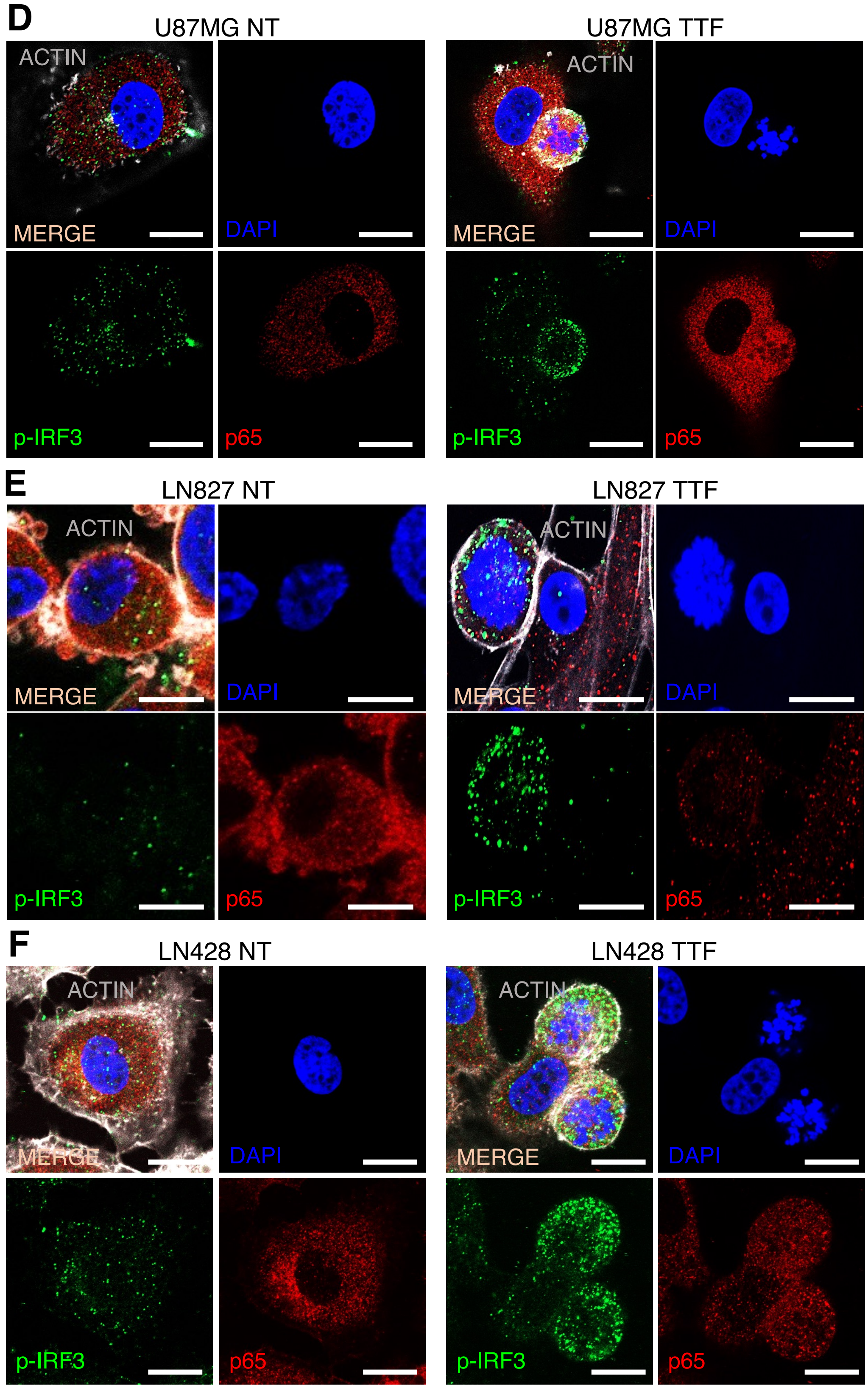


**Figure S8, supporting FIG. 2: The cGAS/STING inflammasome is activated by TTFields in GBM cell lines.**

**A-B)** The cGAS/STING inflammasome's components IRF3 and p65 were activated following 24 hrs of TTFields treatment, as determined by immunoblotting for p-IRF3 and p-p65 in total lysate (**A**) and quantified by densitometry relative to total IRF3 and p65 levels and normalized against B–actin with values for the non-treated condition set at 1 (**B**) in the 3 GBM cell lines.

**C**) Radiograph showing immunoblotting for STING in LN428 GBM cells that was rapidly degraded within 6 hrs of TTFields exposure.

(**D-F**) Confocal IF images demonstrating increased concentration and recruitment of p-IRF3 and p65 within cytosolic micronuclei clusters in U87MG (**D**), LN827 (**E**), and LN428 (**F**) GBM after 24hrs treatment with TTFields. Scale bar: 20µm.

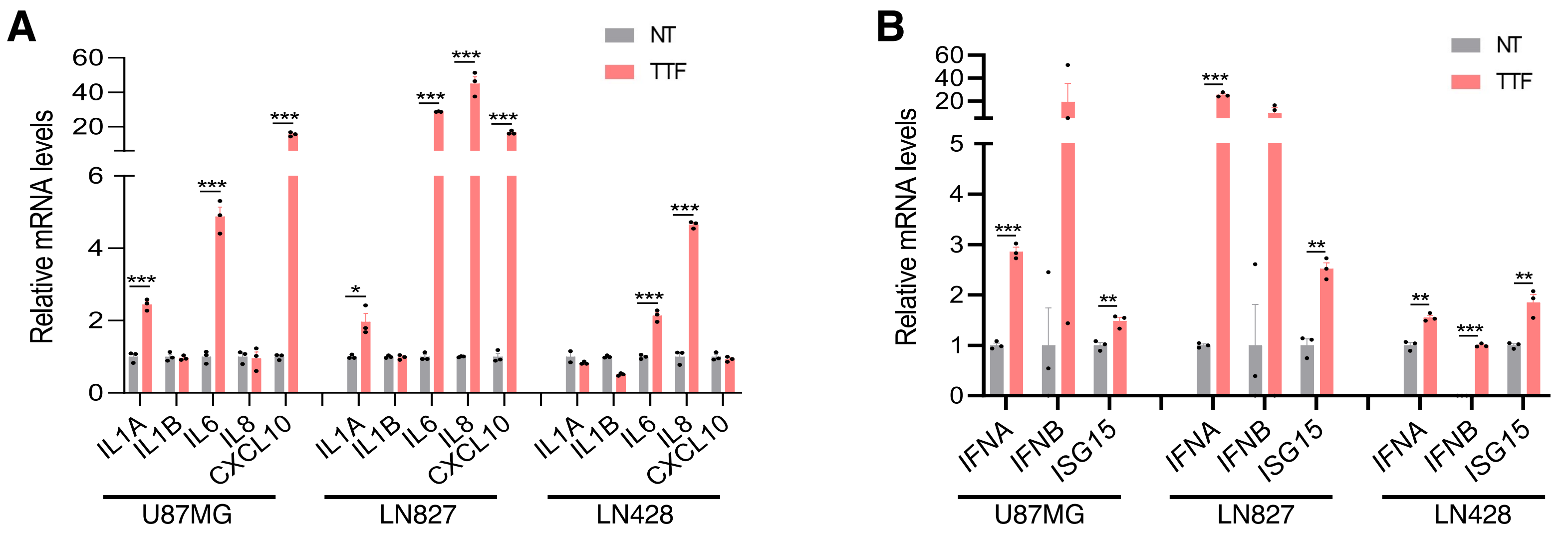


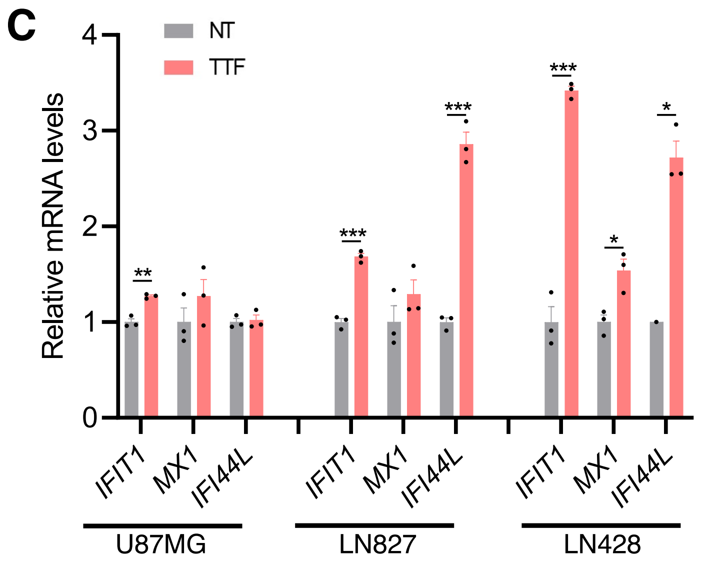


**Figure S9, supporting FIG. 3A-B: TTFields-activated cGAS/STING inflammasome induces PICs, T1IFNs and T1IRGs in GBM cells.**

**A-B**) Combo bar and dot plots demonstrating relative mRNA upregulation of several PICs (**B**) and T1IFNs/T1IRGs (**C**) in the 3 GBM cell lines in response to 24 hr treatment with TTFields.

**C**) A combo bar and dot plot showing relative mRNA upregulation of several additional T1IRGs in the 3 GBM cell lines in response to 24 hrs treatment with TTFields.

**D**) Kinetics of mRNA upregulation of the PIC *IL6* and the T1IRG *ISG15* in response to TTFields in the 3 GBM cell lines showing a peak between 24 and 72 hrs.

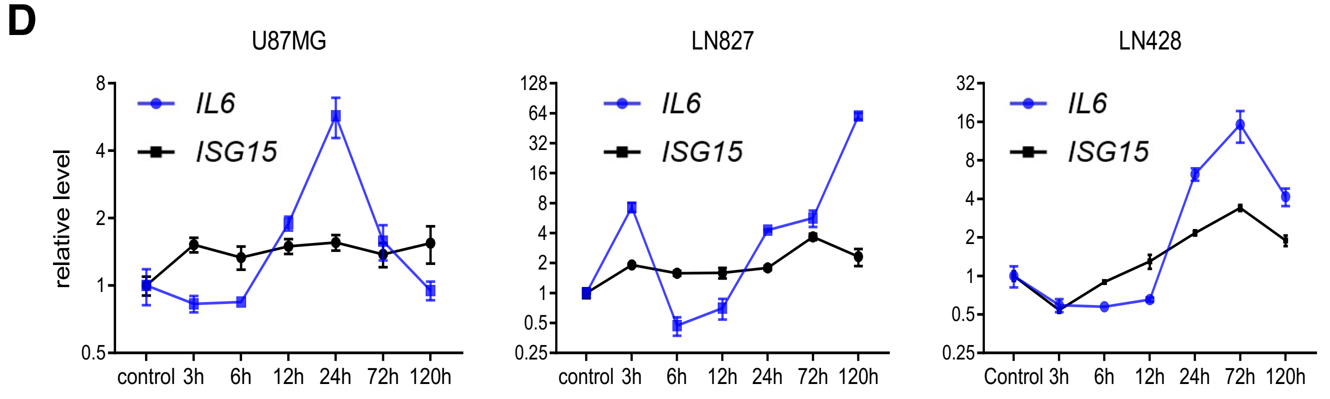

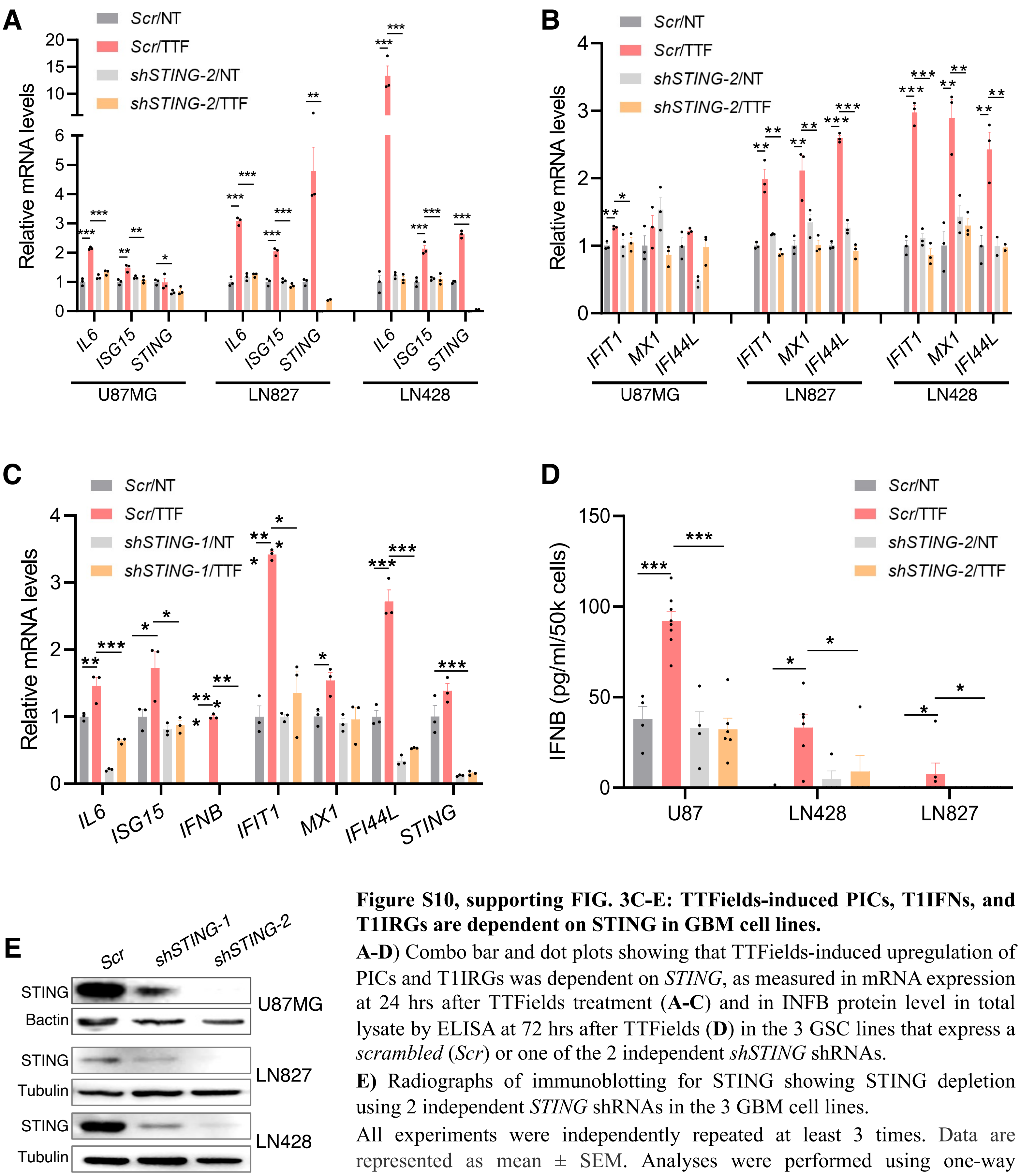


**Figure S10, supporting FIG. 3C-E: TTFields-induced PICs, T1IFNs, and T1IRGs are dependent on STING in GBM cell lines.**

**A-D**) Combo bar and dot plots showing that TTFields-induced upregulation of PICs and T1IRGs was dependent on *STING*, as measured in mRNA expression at 24 hrs after TTFields treatment (**A-C**) and in INFB protein level in total lysate by ELISA at 72 hrs after TTFields (**D**) in the 3 GSC lines that express a *scrambled* (*Scr*) or one of the 2 independent *shSTING* shRNAs.

**E)** Radiographs of immunoblotting for STING showing STING depletion using 2 independent *STING* shRNAs in the 3 GBM cell lines.

All experiments were independently repeated at least 3 times. Data are represented as mean ± SEM. Analyses were performed using one-way ANOVA. *, $P$ <0.05; **, $P$ <0.01; ***, $P$ <0.001.

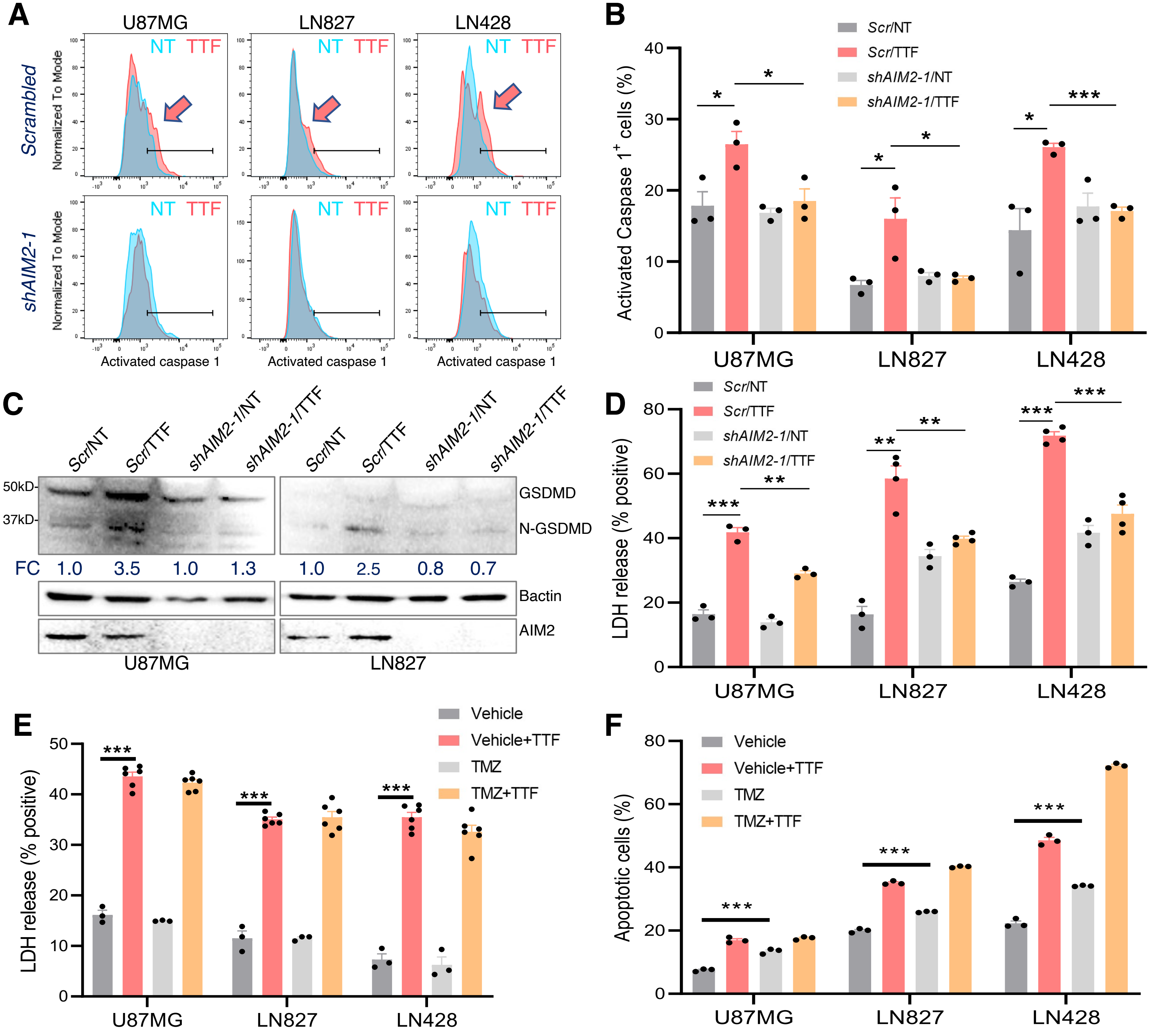


**Figure S11, supporting FIGS. 4-5: TTFields activate the AIM2/Caspase-1 inflammasome in GBM cell lines.**
**A-B**) Caspase-1 activation level following 24 hrs of TTFields treatment, as determined by FAM-YVAD-FMK in the 3 GBM cell lines that expressed a *Scrambled (Scr)* or *shAIM2-1* (**A**) and summarized in a bar and dot plot (**B**).
**C**) Radiographs of immunoblotting for GSDMD showing the Caspase-1 cleaved product (N-GSDMD) in total lysates from U87MG and LN827 GBM cell lines that expressed a *Scrambled (Scr)* or *shAIM2-2* shRNA and were either non-treated or treated with TTFields for 24 hrs. Shown is also fold change in density of the N-GSDMD relative to the full-length GSDMD and normalized against B-actin with values for the non-treated *Scrambled* set at 1.
**D**) A combo bar and dot plot of an LDH release assay showing TTFields-induced plasma membrane disruption in a *AIM2*-dependent manner following 24 hrs of TTFields treatment as determined by LDH release into the supernatants in the 3 GBM cell lines that expressed a *Scrambled (Scr)* or *shAIM2-2*.
**E**) A combo bar and dot plot of an LDH release assay showing that TTFields-induced membrane damaged cell death in the 3 GBM lines following 24 hrs of TTFields treatment is distinct from apoptosis caused by TMZ (150μM for 24 hrs.).
**F**) A combo bar and dot plot showing that the 3 GBM cell lines treated with TMZ (150μM for 24 hrs.) exhibited increased apoptosis measured by the fraction of cells that are Annexin V positive and viability dye negative.
All experiments were independently repeated at least 3 times. Data are represented as mean ± SEM. Analyses were performed using one-way ANOVA. *, $P$ <0.05; **, $P$ <0.01; ***, $P$ <0.001.

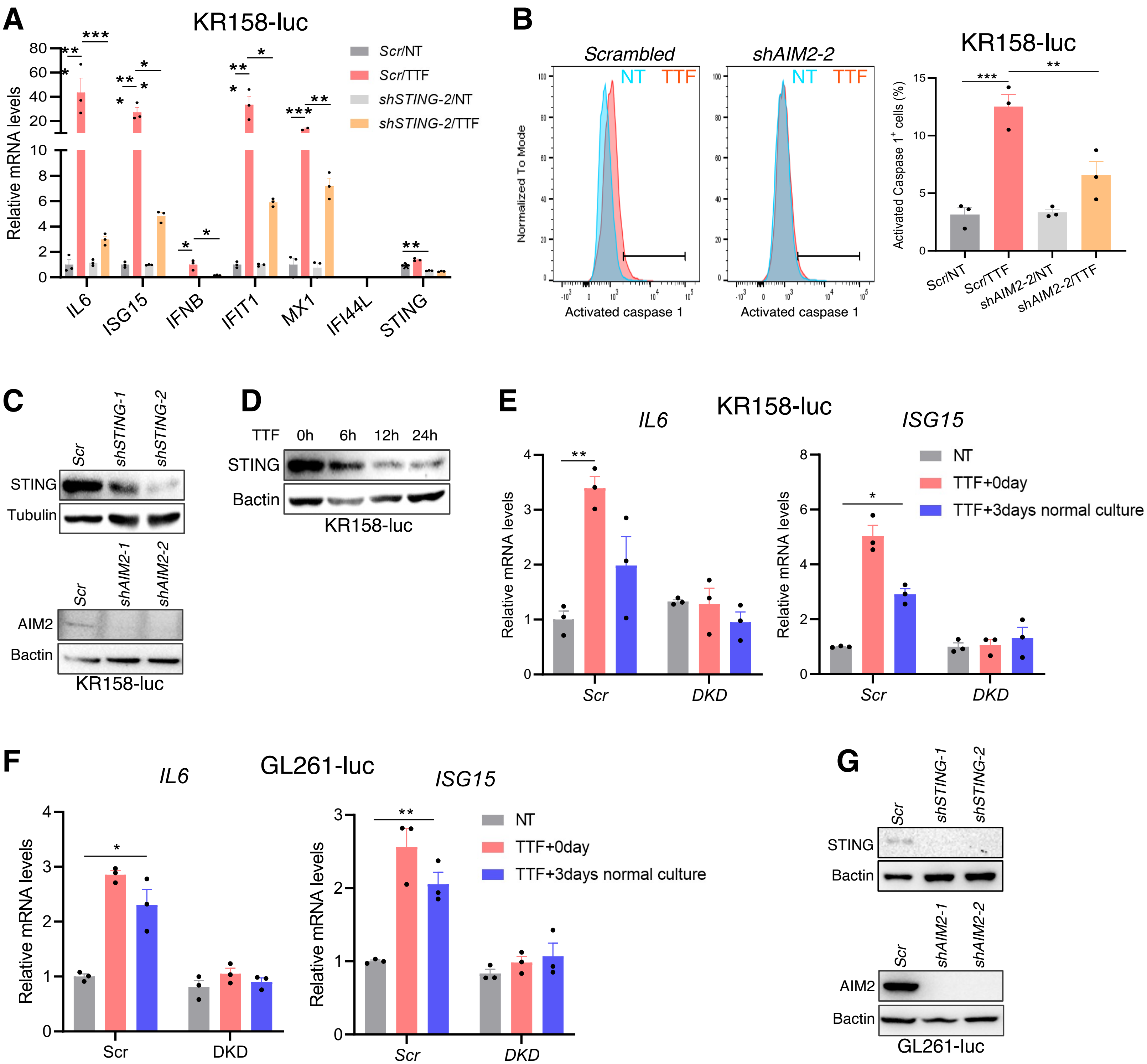


**Figure S12, supporting FIGS. 6-8: TTFields induce the cGAS/STING and AIM2/Caspase-1 inflammasomes in KR158-luc and GL261-luc mouse GBM cells.**

**A-C**) TTFields stimulate the cGAS/STING (**A**) and AIM2/Caspase-1 (**B**) inflammasomes in KR158-luc cells in a *STING* and *AIM2*-dependent manner.

**C**) Radiographs showing immunoblotting for STING and AIM2 in KR158-luc cells expressing the *Scrambled* shRNA (*Scr*) or one of 2 independent *STING* (*shSTING-1* and *2*) and *AIM2* (*shAIM2-1* or *2*) shRNAs.

**D**) Radiographs of immunoblotting for STING in KR158-luc cells show rapid degradation after TTFields exposure.

**E-F**) Combo bar and dot plots showing that the PIC *IL6* and T1IRG *ISG15* remained upregulated in KR158-luc (**E**) and GL261-luc (**F**) cells in a *STING*- and *AIM2*-dependent manner for at least 3 days after TTFields cessation.

**G**) Radiographs showing immunoblotting for STING and AIM2 in GL261-luc cells expressing the *Scrambled* shRNA (*Scr*) or one of 2 independent *STING* or *AIM2* shRNAs.

All experiments were repeated at least 3 times. Data are represented as mean ± SEM. Analyses were performed one-way ANOVA. *, $P$ <0.05; **, $P$ <0.01; ***, $P$ <0.001.

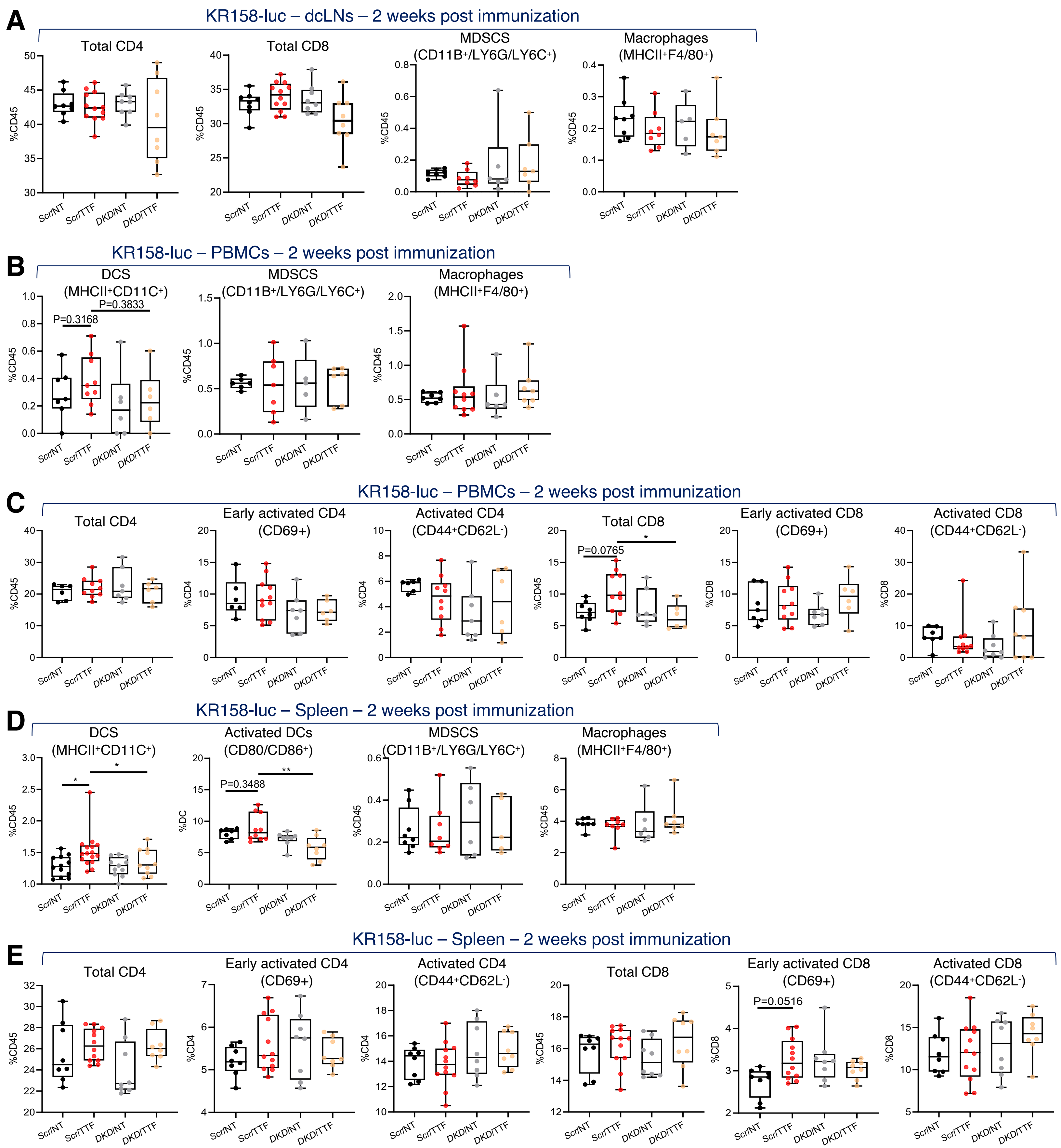


**Figure S13, supporting FIG. 9: Induction of anti-tumor immunity in the KR158-luc GBM model by TTFields requires *STING* and *AIM2*.**

Combo box and whisker and dot plots showing immunophenotyping of C57BL/6J mice at 2 weeks post immunization with KR158-luc in various conditions (n=7-12 mice for each cohort) as in **FIGURE 7B-F** for the fractions of total CD4, MDSCs and macrophages in dcLNs (**A**), total DCs, MDSCs, and macrophages in PBMCs (**B**), total, early and fully activated $CD4^+$ and $CD8^+$ T cells in PBMCs (**C**), total DCs, activated DCs, MDSCs and macrophages (**D**) and total, early and fully activated $CD4^+$ and $CD8^+$ T cells in splenocytes (**E**). Data are represented as mean ± SEM. The whiskers are the minimum and maximum values, the lower and upper box edges the 25th and 75th percentage values, respectively, and the lines within the boxes the median. Comparisons were performed using one-way ANOVA. *, p<0.05; **, p<0.01.

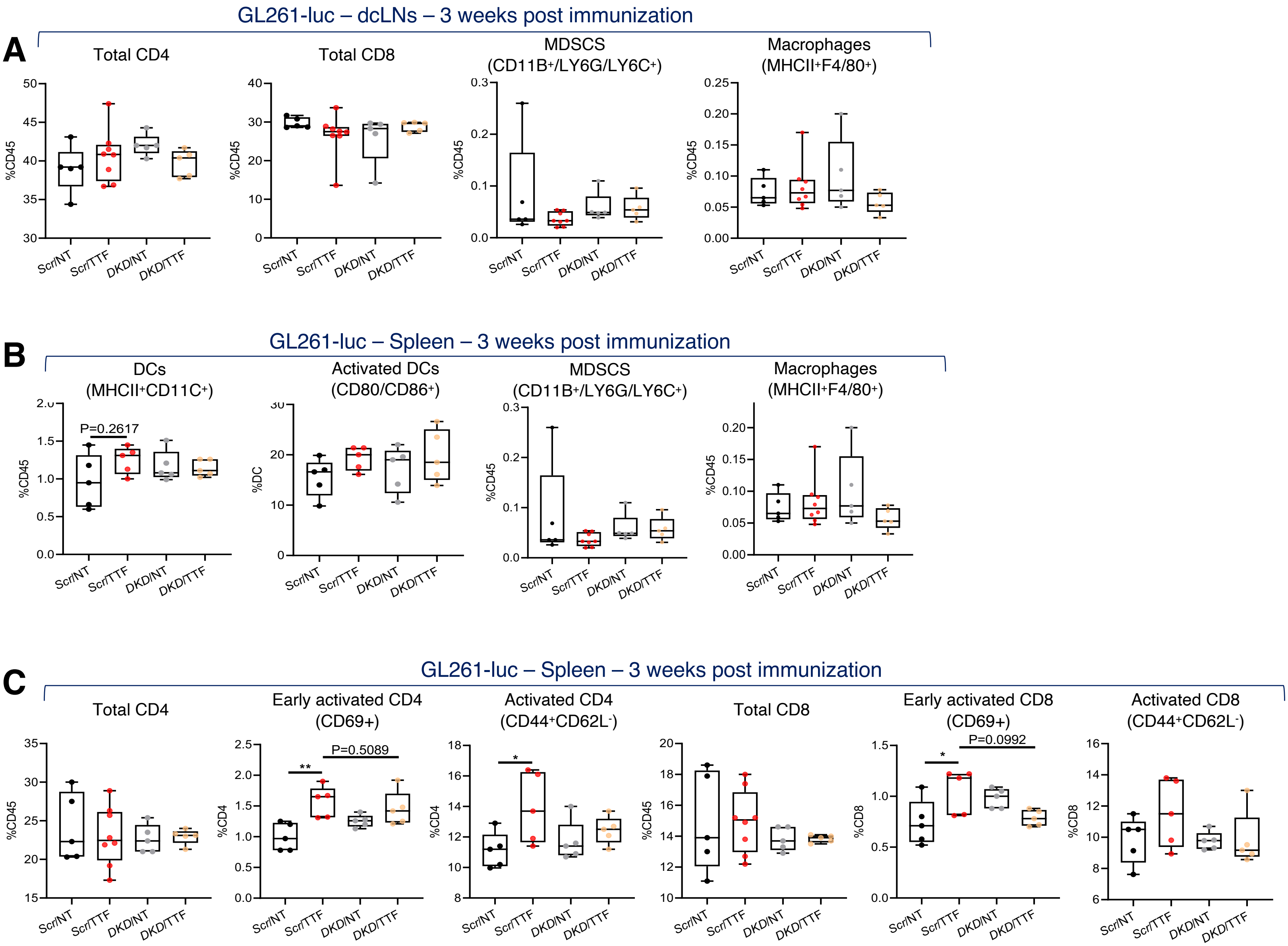


**Figure S14, supporting FIG. 10: Induction of anti-tumor immunity in the GL261-luc GBM model by TTFields requires *STING* and *AIM2*.**

Combo box and whisker and dot plots showing immunophenotyping of C57BL/6J mice at 3 weeks after being immunized with GL261-luc cells in various conditions (n=8 for *Scr*/TTF and n=5 for the other 3 cohorts) as in **FIGURE 8A-E** for the fractions of total CD4 and CD8 T cells, MDSCs and macrophages in dcLNs (**A**), total DCs and activated DCs, MDSCs and macrophages in splenocytes (**B**), and total, early and fully activated $CD4^+$ and $CD8^+$ T cells in splenocytes (**C**)

Data are represented as mean ± SEM. The whiskers are the minimum and maximum values, the lower and upper box edges the $25^{th}$ and $75^{th}$ percentage values, respectively, and the lines within the boxes the median. Comparisons were performed using one-way ANOVA. *, p<0.05; **, p<0.01.

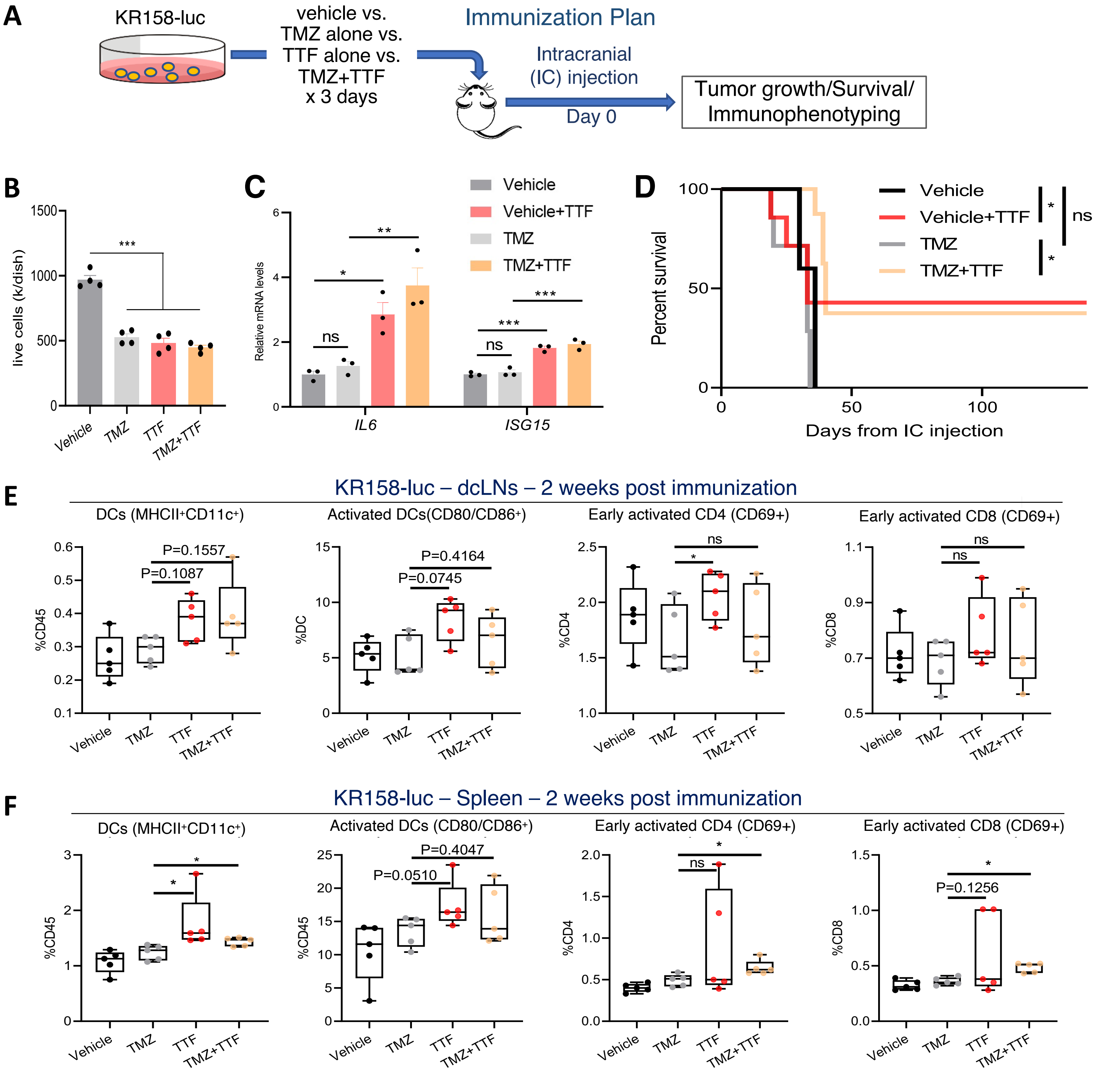


**Figure S15, supporting FIGS. 7-10: TMZ-induced cytotoxicity does not contribute to the induction of anti-tumor immunity in KR158-luc GBM by TTFields.**

**A)** A schema detailing the immunization protocol using KR158-luc cells pre-treated with the vehicle, TMZ alone, TTF alone, or a combination of TTF plus TMZ.

**B)** A combo bar and dot plot confirming the similar cytotoxic effects of TMZ (300 µM), TTFields (200kHz) and the combination treatment for 72 hrs.

**C)** A combo bar and dot plot showing that TMZ (300µM) treatment alone does not activate the PIC *IL6* and the T1IRG *ISG15* as compared to TTFields alone or the combination of TTFields plus TMZ.

**D**) Kaplan-Meier estimate showing survival rates of C57BL/6J mice injected with KR158-luc cells pre-treated with various treatments in **A-B** (n=10 per cohort). Log-rank test was used to compare survival rates. ***, $P$ <0.001.

**E-F**) Combo box and whisker and dot plots showing immunophenotyping of C57BL/6J mice at 2 weeks after being immunized with KR158-luc cells in various conditions in **B-D** (n=5 per cohort) for the fractions of total DCs, activated DCs, and early activated CD4 and CD8 T cells in dcLNs (**D**) and splenocytes (**E**). Data are represented as mean ± SEM. Comparisons were performed using one-way ANOVA. *, p<0.05; **, p<0.01.

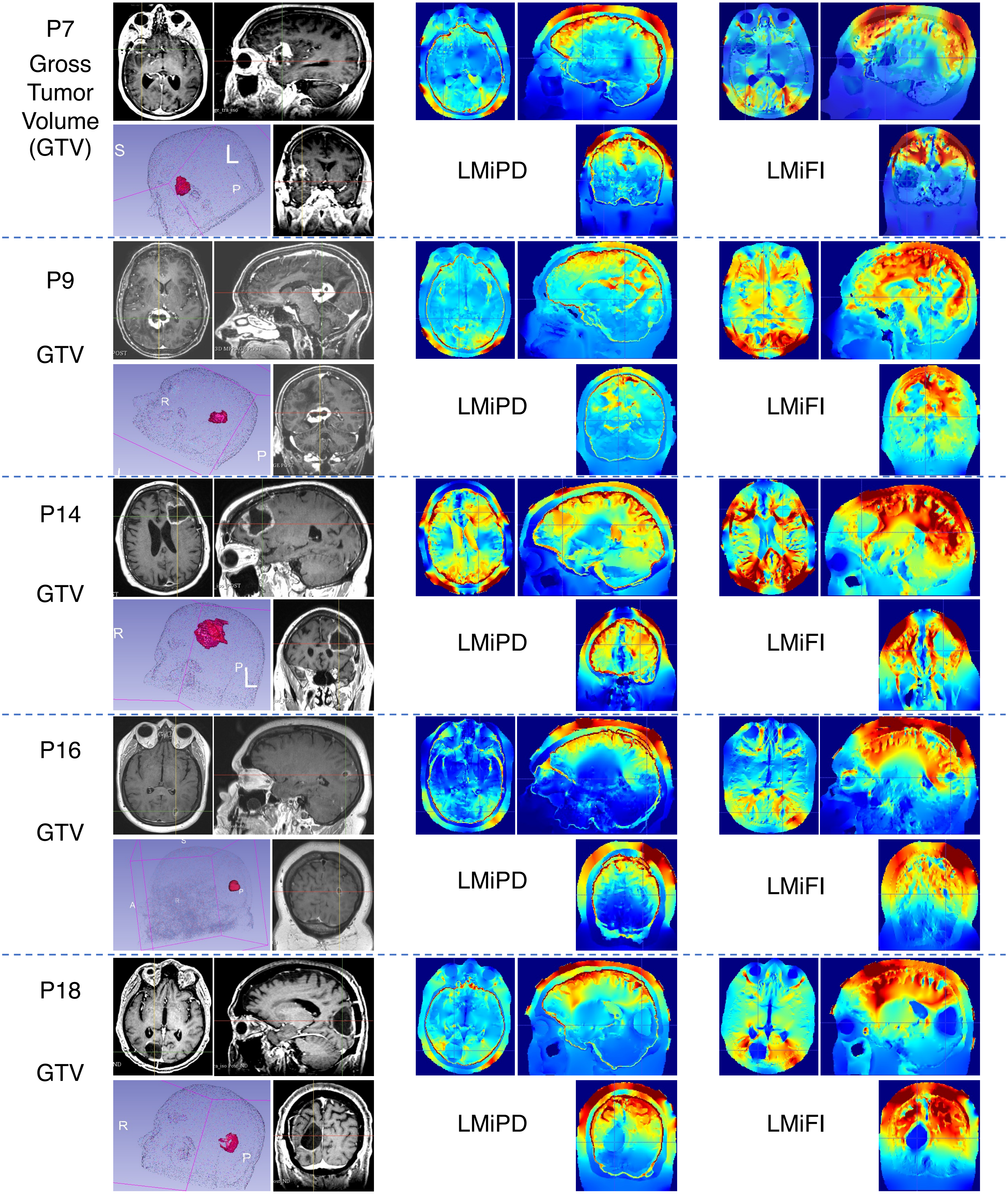


**Figure S16, supporting FIG. 11A: Field distribution for P7, P9, P14, P16 and P18**

GTV including the enhancing rim and the necrotic core or the resection cavity of the dominant lesion based on the brain MRI at the pre-TTFields planning stage (left panel) and representative scale-adjusted heatmap images of LMiPD and LMiFI models at the level of the lesion center at 1 month post TTFields (right 2 panels) of the indicated patients.

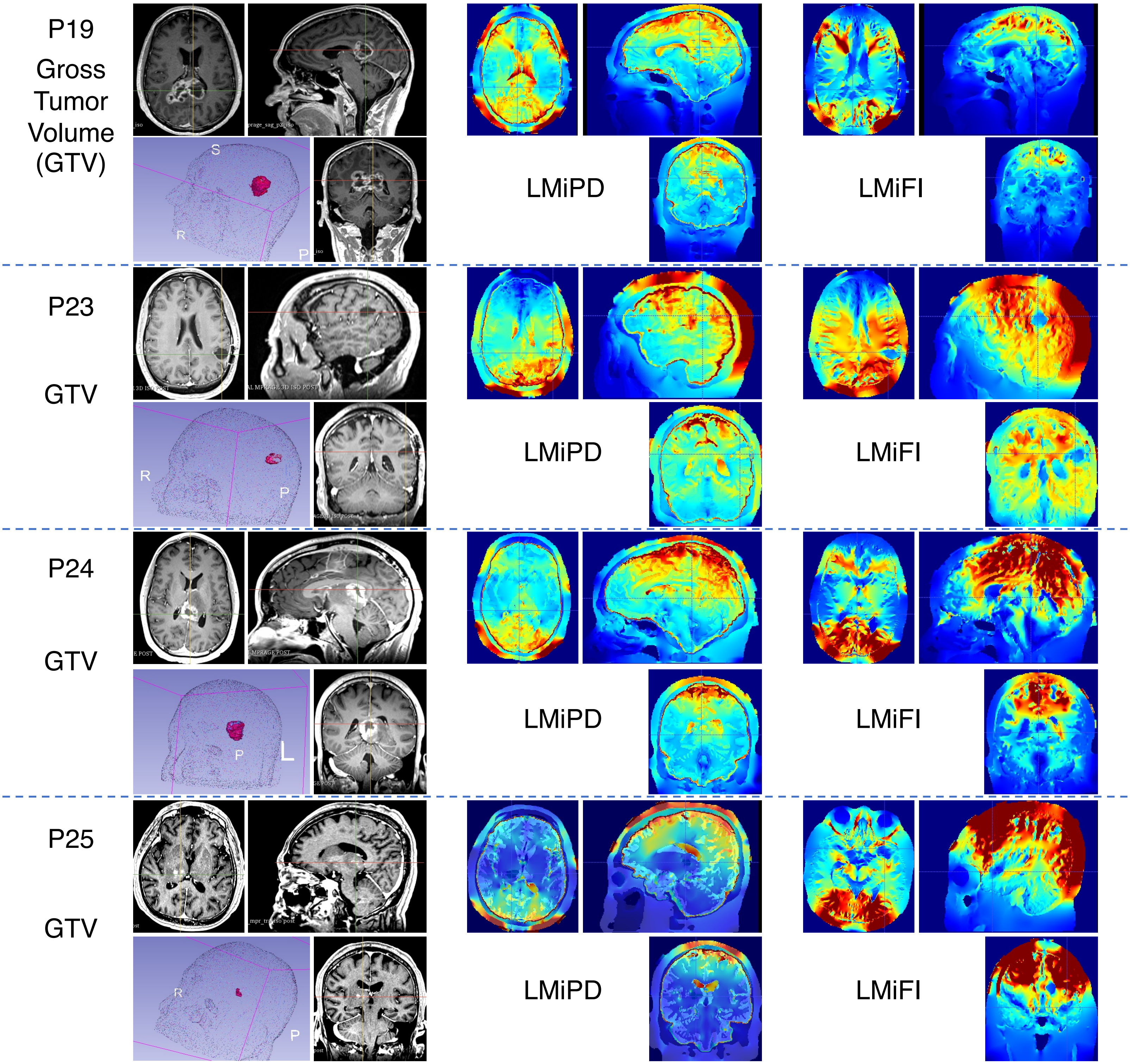


**Figure S17, supporting FIG. 11A: LMiPD and LMiFI for P19, P23, P24 and P25**

Gross tumor volume (GTV) including the enhancing rim and the necrotic core or the resection cavity of the dominant lesion based on the brain MRI at the pre-TTFields planning stage (left panel) and representative scale-adjusted heatmap images of LMiPD and LMiFI models at the level of the lesion center at 1 month post TTFields (right 2 panels) of the indicated patients.

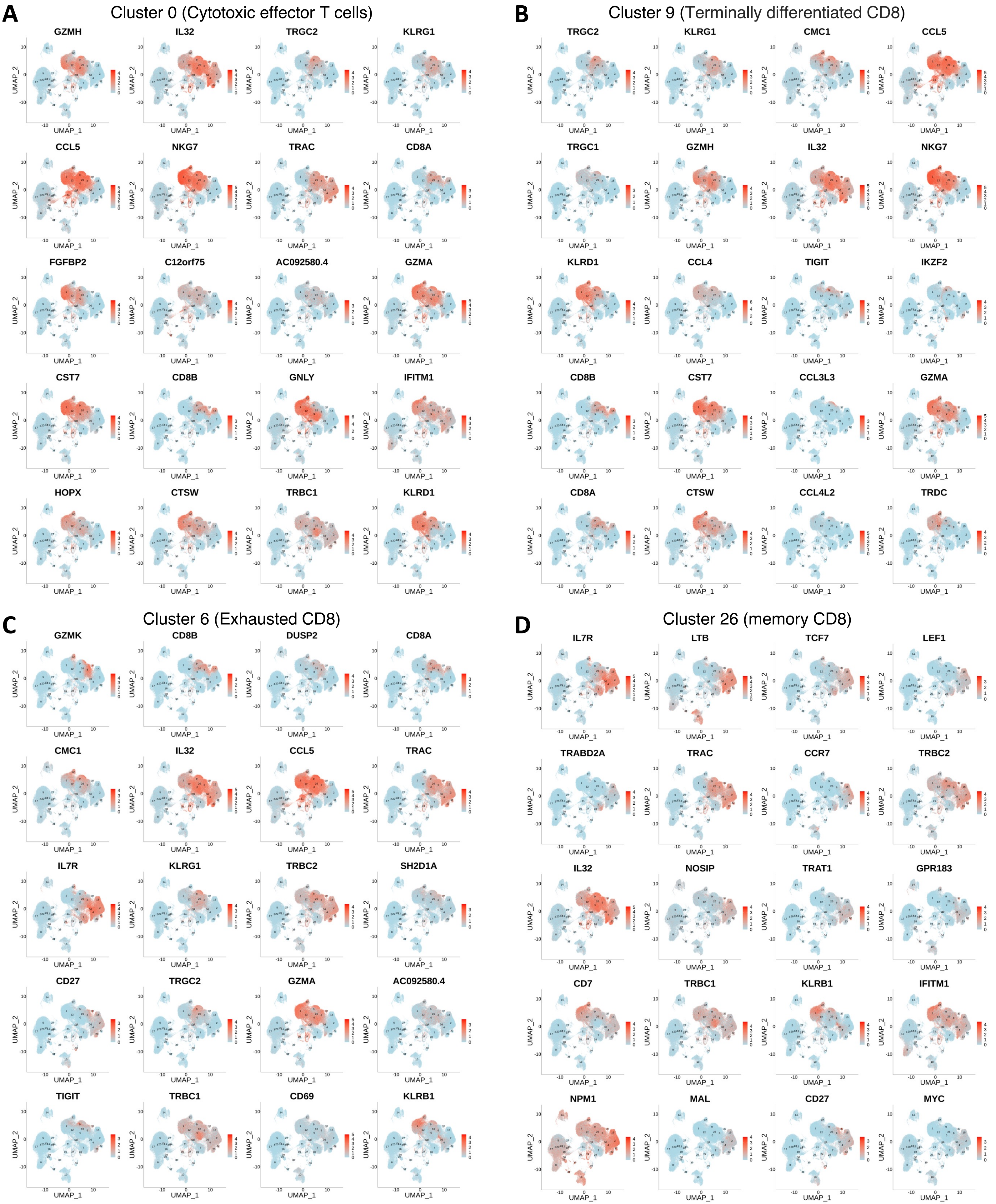


**Figure 18, supporting FIG. 11B-D: Markers for Clusters 0, 9, 6, and 26 by scRNA-seq in 12 GBM patients**

Heatmaps of expression of indicated marker genes for Cluster 0 (cytotoxic effectors) (**A**), Cluster 9 (terminally differentiated CD8 effectors) (**B**), Cluster 6 (exhausted effectors) (**C**), and Cluster 26 (memory CD8 T cells) (**D**) at the single cell level showing their expression distribution across all clusters in the UMAP graph at Resolution 1.

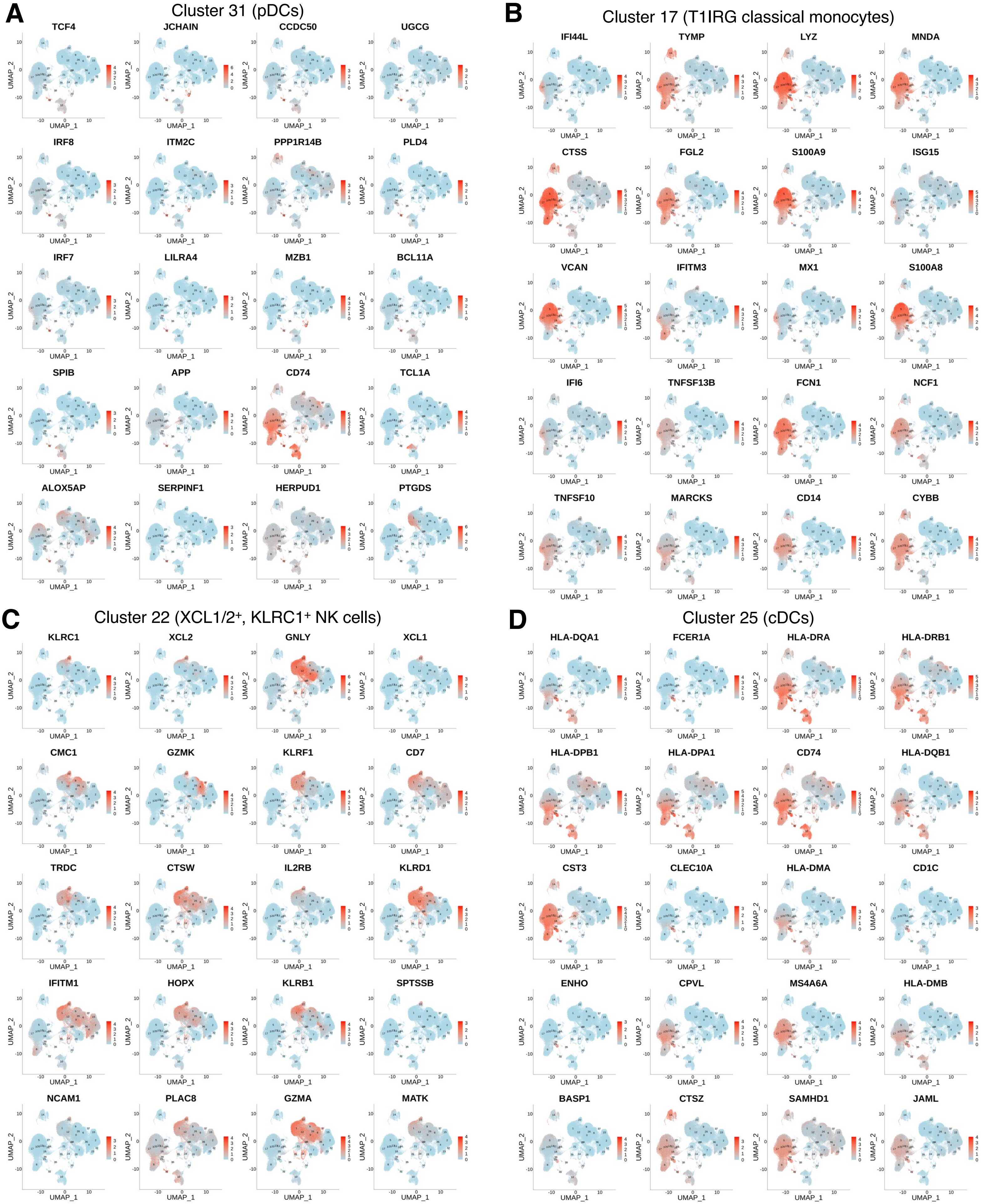


**Figure S19, supporting FIG. 11B: Markers for Clusters 31, 25, 17, and 22 by scRNA-seq in 12 GBM patients**

Heatmaps of expression of indicated marker genes for Cluster 31 (pDCs) (**A**), Cluster 17 (T1IRG classical monocytes) (**B**), Cluster 22 (XCL1/2+, KLRC1+ NK cells) (**C**), and Cluster 25 (cDCs) (**D**), at the single cell level showing their expression distribution across all clusters in the UMAP graph at Resolution 1.

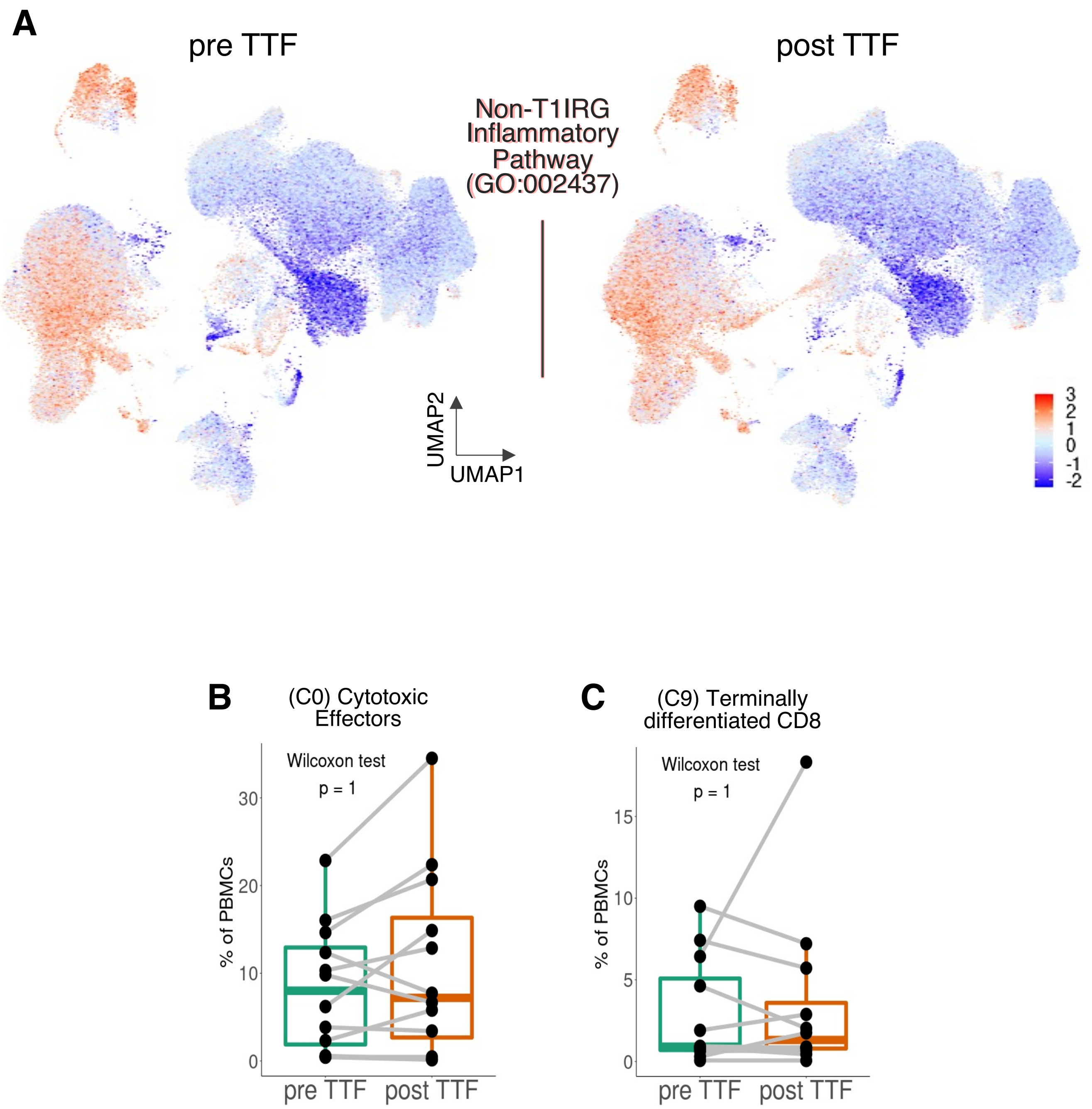


**Figure S20, supporting FIG. 12E-G: Distribution of a non-T1IRG pathway and proportions of C0 and C9 in PBMCs of GBM patients in response to TTFields.**

**A)** A heatmap of mean expression levels of the non-T1IRG inflammatory pathway GO:002437 at the single cell level in pre-TTF and post-TTF PBMCs.

**B-C**) Combo box and whisker and paired dot plots showing the proportions of cytotoxic effectors (C0) (**B**) and terminally differentiated CD8 effectors (C9) **(C)** as percent of total PBMCs in pre-TTF and post-TTF PBMCs. Analysis was performed using the Wilcoxon test.

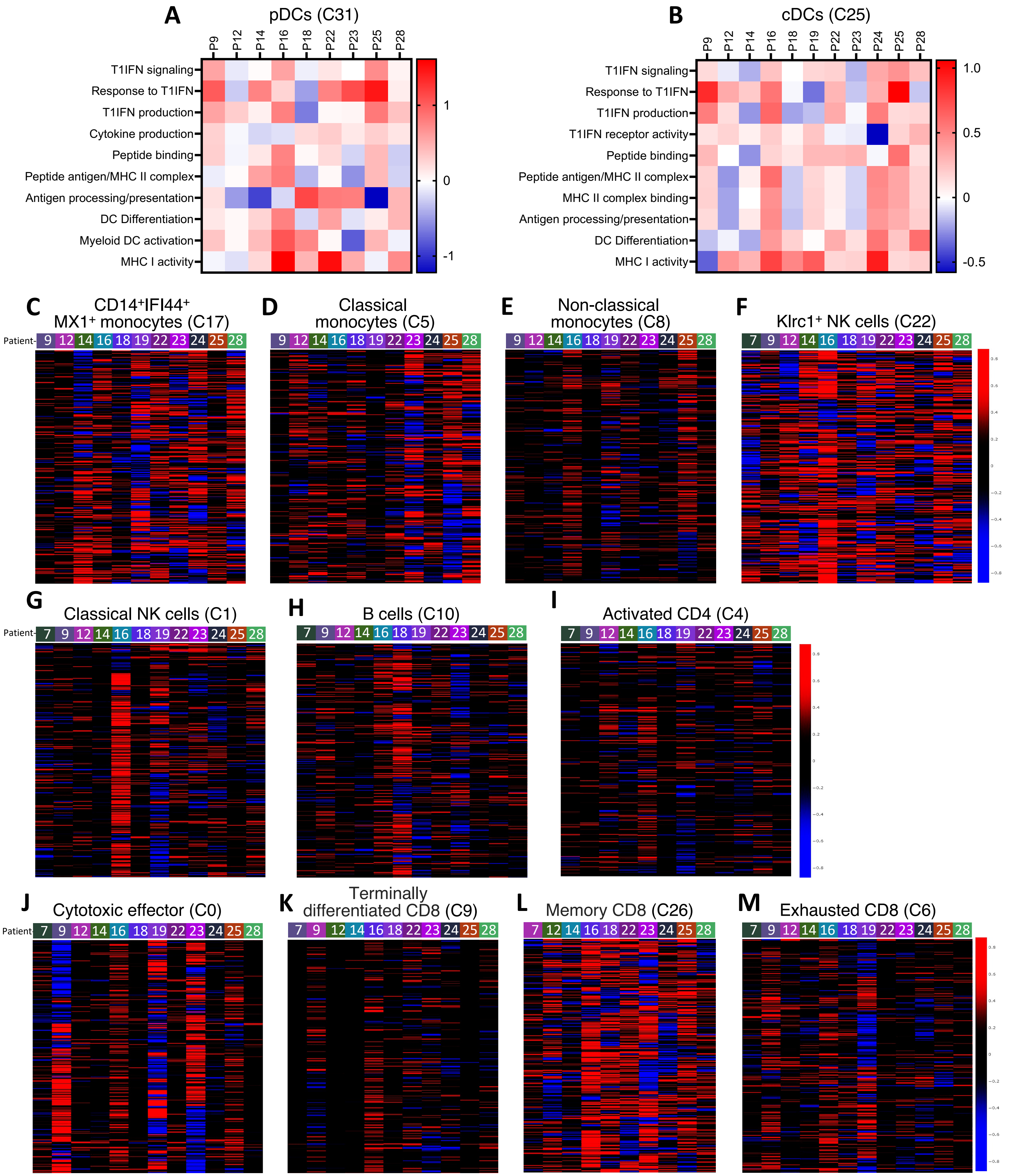


**Figure S21, supporting FIG. 12B-K: TTFields effects at all genes in various PBMC clusters in GBM patients.**
**A-B**) Heatmaps of gene expression showing logFC of post-TTFields expression compared to pre-TTFields expression of 10 functionally critical pathways in pDCs (C31) (**A**) and cDCs (C25) (**B**) showing post-TTFields activation of T1IFN and T1IRG pathways and DC-critical pathways.
**C-M)** Heatmaps of gene expression showing logFC of post-TTF compared to pre-TTF expression of all genes in the indicated cell clusters in patients with detectable pre- and post TTFields counts in the respective cell clusters.

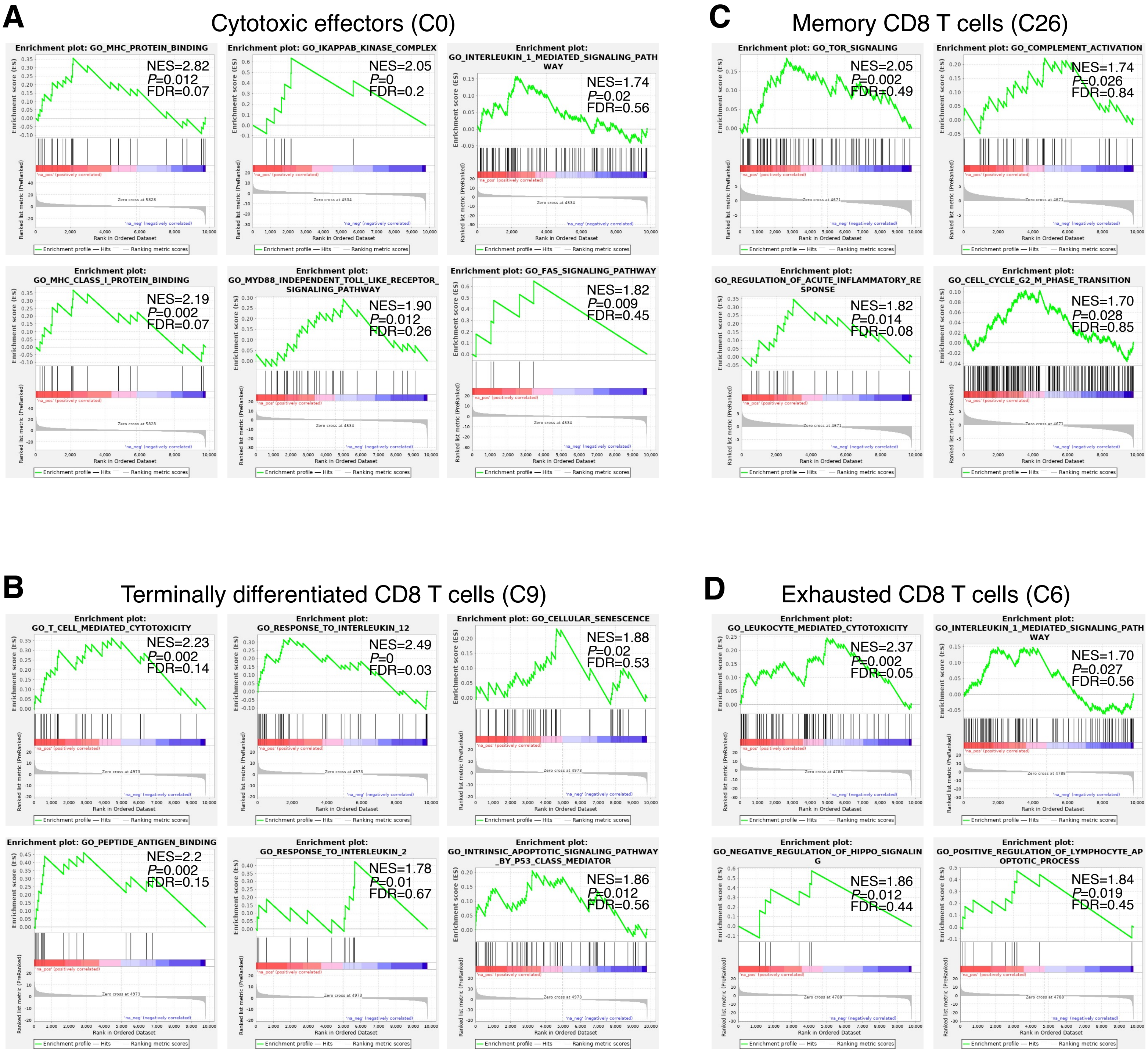


**Figure S22, supporting FIG. 12F-G: Activation of pathways critical for the cytotoxic T cell functions after TTFields treatment.**

Plots of GSEA of functionally critical pathways showing their post-TTFields enrichment or activation in Cytotoxic effectors (C0) (**A**), terminally differentiated CD8 effectors (C9) (**B**), memory CD8 T cells (C26) (**C**), and exhausted CD8 effectors (C6) (**D**) in PBMCs of GBM patients.

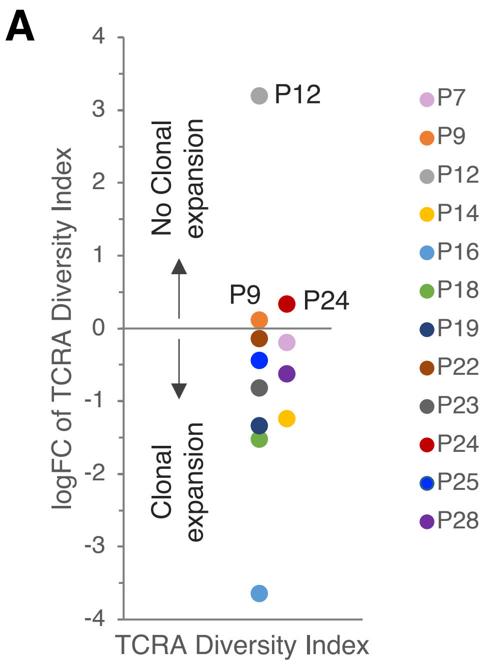


**Figure S23, supporting FIG. 13**: TCRA clonal expansion correlates with TTFields treatment in GBM patients

**A)** A dot plot of logFC of the Simpson Diversity Index (DI) of TCRA showing TCRA clonal expansion after TTFields treatment (negative DI logFC) in 9 of 12 patients.

**B)** 2D area charts of the 200 most abundant TCRA clones in post TTFields T cells as compared to their proportions in pre-TTFields T cells showing clonal expansion in all 12 patients.

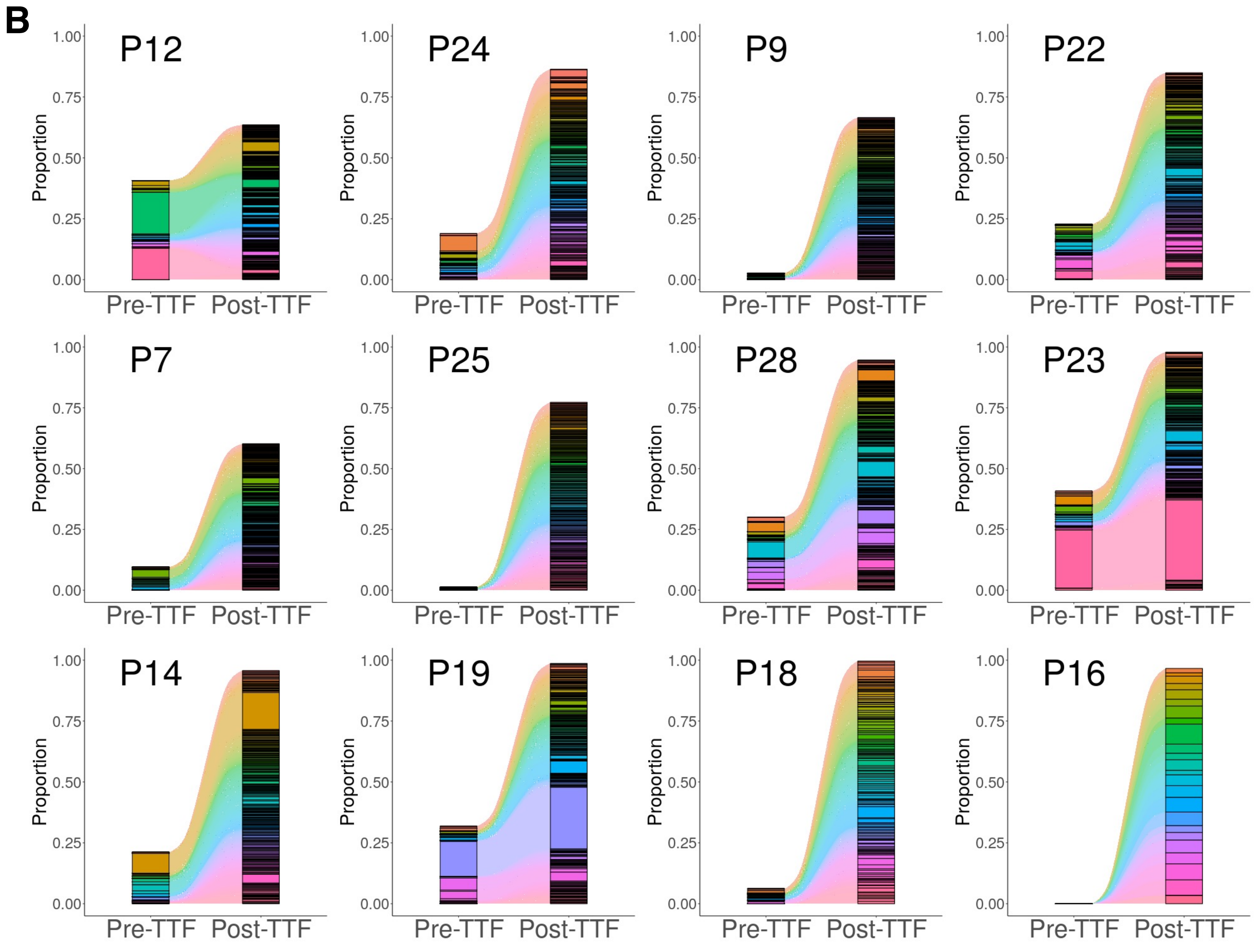

Supplement: Supplemental data [file jci-132-149258-s082.pdf]
